# Exon Junction Complexes can have distinct functional flavours to regulate specific splicing events

**DOI:** 10.1038/s41598-018-27826-y

**Published:** 2018-06-22

**Authors:** Zhen Wang, Lionel Ballut, Isabelle Barbosa, Hervé Le Hir

**Affiliations:** 1 Institut de biologie de l’Ecole normale supérieure (IBENS), Ecole normale supérieure, CNRS, INSERM, PSL Research University, Paris, F-75005 France; 2 Institut de Biologie et Chimie des Protéines, CNRS UMR5086, Université de Lyon 1, Lyon, France

## Abstract

The exon junction complex (EJC) deposited on spliced mRNAs, plays a central role in the post-transcriptional gene regulation and specific gene expression. The EJC core complex is associated with multiple peripheral factors involved in various post-splicing events. Here, using recombinant complex reconstitution and transcriptome-wide analysis, we showed that the EJC peripheral protein complexes ASAP and PSAP form distinct complexes with the EJC core and can confer to EJCs distinct alternative splicing regulatory activities. This study provides the first evidence that different EJCs can have distinct functions, illuminating EJC-dependent gene regulation.

## Introduction

The Exon Junction Complex (EJC) plays a central role in post-transcriptional gene expression control. EJCs tag mRNA exon junctions following intron removal by spliceosomes and accompany spliced mRNAs from the nucleus to the cytoplasm where they are displaced by the translating ribosomes^1,2^. The EJC is organized around a core complex made of the proteins eIF4A3, MAGOH, Y14 and MLN51, and this EJC core serves as platforms for multiple peripheral factors during different post-transcriptional steps^3,4^. Dismantled during translation, EJCs mark a very precise period in mRNA life between nuclear splicing and cytoplasmic translation. In this window, EJCs contribute to alternative splicing^5–7^, intra-cellular RNA localization^8^, translation efficiency^9–11^ and mRNA stability control by nonsense-mediated mRNA decay (NMD)^12–14^. At a physiological level, developmental defects and human pathological disorders due to altered expression of EJC proteins show that the EJC dosage is critical for specific cell fate determinations, such as specification of embryonic body axis in drosophila, or Neural Stem Cells division in the mouse^8,15,16^. These observations strongly suggest the implication of EJC in fine-tuning of specific gene expression in different cellular contexts.

Interestingly, EJCs are not loaded equally across all exon junctions of a mRNA. Functional studies and transcriptome-wide analyses revealed that EJC loading can vary between junctions within a given transcript^8,17–19^. In fly and human, EJCs participate to specific splicing choices notably by serving, once loaded, as a splicing enhancer of neighboring introns^5–7,20^. Another mean by which EJC could differentially modulate transcript destiny would be that each EJC has different composition, a possibility unexplored so far. During splicing, the core EJC is associated with peripheral proteins, including ACINUS, PININ, RNPS1 and SAP18 are known splicing factors^4,21–23^. ACINUS and PININ are scaffold proteins that form two alternative ternary complexes with RNPS1 and SAP18, named ASAP and PSAP, respectively^24^. However, how these complexes bind the EJC core and whether they function differently as part of EJC is still unknown. Here, we performed biochemical experiments to show that ACINUS bridges the ASAP complex to the EJC core. Transcriptome-wide RNA-seq studies revealed that ASAP and PSAP complexes regulate distinct alternative splicing events both in EJC-independent and EJC-dependent manner.

## Results and Discussion

### ACINUS bridges the ASAP complex to the EJC core

**Figure 1:**
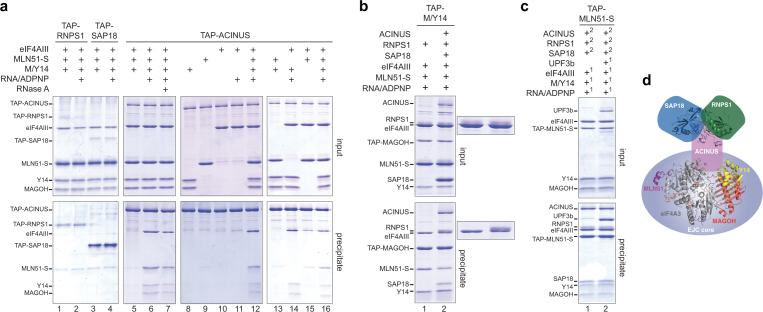
ACINUS bridges the ASAP complex to the EJC core. (**a**) Coprecipitations with TAP-RNPS1, TAP-SAP18 or TAP-ACINUS mixed with eIF4AIII, MLN51-S and/or MAGOH/Y14 with or without ADNP and 47-mer ssRNA. Protein mixtures before (input, 20% of total) or after precipitation (precipitate) were separated on 10%(w/v) acrylamide SDS-PAGE. Mixture was treated by RNaseA before precipitation when indicated. (**b**) Coprecipitations as in (a), except that TAP-MAGOH/Y14 was mixed with eIF4A3, MLN51-S, RNPS1, ADPNP and ssRNA with or without ACINUS and SAP18. (**c**) Coprecipitations as in (a), except that TAP-MLN51-S was first mixed (1) with eIF4A3, MAGOH/Y14, ADPNP, ssRNA with or without UPF3b. After washing, mixtures were then mixed (2) with ACINUS, RNPS1 and SAP18. (**d**) Schematic representation of ACINUS bridging ASAP (PDB 4A8X) and to EJC (PDB 2XB2) core complexes.

To determine how the ASAP complex interacts with the EJC core, we performed *in vitro* coprecipitation assays^3^ with Tandem Affinity Purification (TAP)-tagged recombinant proteins corresponding to full-length human SAP18, RNPS1 and ACINUS. The reconstitution of a stable EJC core requires single-stranded RNA (ssRNA) and ATP (or its non-hydrolysable form, ADPNP), and in the absence of either, the complex is not formed^3^. Individual ASAP proteins were incubated with recombinant EJC core proteins either in the presence or absence of ssRNA and ADPNP. Hence, we could test whether individual ASAP proteins interact with EJC core components individually or with the EJC core complex. Neither TAP-RNPS1 nor TAP-SAP18 co-precipitated EJC core proteins either individually or as a complex (Fig. 1a, lanes 1–4). In contrast, TAP-ACINUS co-precipitated the four EJC core proteins only in the presence of ssRNA and ADPNP (Fig. 1a, lanes 5–6). TAP-ACINUS interacted with the reconstituted EJC core via direct protein-protein interaction because the precipitation of the EJC core was not affected by RNase treatment after protein interaction (Fig. 1a, lanes 6–7). Interaction assays with EJC core proteins separately showed that TAP-ACINUS did not interact with individual EJC core proteins (Fig. 1a, lanes 9–11). Tests with combinations of two components revealed that TAP-ACINUS directly contacts a sub-complex formed by eIF4A3 and MAGOH/Y14 in the presence of ssRNA and ADPNP (Fig. 1a, lanes 8–16). Therefore, eIF4AIII and MAGOH/Y14 may constitute a composite binding site for ACINUS while neither RNPS1 nor SAP18 directly bind the EJC core.

To determine whether ACINUS binding to EJC is compatible with ASAP complex assembly, we mixed EJC core proteins including TAP-MAGOH/Y14 with RNPS1 with or without ACINUS and SAP18 (Fig. 1b). RNPS1 that does not interact with MAGOH/Y14 (Fig. 1a), was efficiently precipitated by TAP-MAGOH/Y14 only in the presence of ACINUS and SAP18, demonstrating that ACINUS bridges EJC core and ASAP complexes. The presence of RNPS1 and SAP18 also did not impact the binding ability of ACINUS to EJC core (Supplementary Fig. 1), indicating that a fully assembled ASAP complex is not required to interact with the EJC core. MAGOH/Y14 and eIF4AIII forms a composite binding site for the NMD factor UPF3b^25^. To test whether UPF3b binding is compatible with ASAP interaction with the EJC core, EJC cores with or without UPF3b bound were reconstituted with TAP-MLN51 before incubation with ASAP components. The co-precipitation of ASAP by EJC core was not altered by the presence of UPF3b (Fig. 1c). Taken together, ACINUS bridges the ASAP complex to EJC core (Fig. 1d) and this interaction is compatible with UPF3b uncovering that ACINUS and UPF3b contact distinct regions of the EJC core surface. We could not produce full-length recombinant PININ to test its binding to the EJC core but given that that neither RNPS1 nor SAP18 directly contact the EJC core the PSAP complex most likely binds to the EJC core via PININ. Whether the two complexes can simultaneously bind the EJC core or use the same binding mode and are therefore mutually exclusive remains an open question.

### ASAP and PSAP regulate different splicing

Since both ASAP and PSAP complexes can exist in cells, and all components are splicing regulators, we asked how depletion of each component of ASAP and PSAP complex can impact gene expression, in particular alternative splicing. To this end, we performed 2 biological replicates of mRNAseq experiments in HeLa cells depleted with ACINUS, PININ and RNPS1, respectively (Supplementary Fig. 2, Table 1). None of the KD significantly affected other proteins either at the mRNA or the protein level (Supplementary Fig. 2). The number of reads mapped per gene is highly correlated between the two replicate experiments (Supplementary Fig. 2). Both experiments generated a combined 29, 69, 79 and 70 million uniquely mapped reads for GFP, ACINUS, RNPS1 and PININ KD, respectively (Supplementary Table 1). We performed differential splice variant analysis as in previous study^7,26,27^. Briefly, we used two different programs to identify differentially spliced events, MISO^27^ and DiffSplice^26^. Only splicing events with changes more than 10% were considered, and significant splicing events identified either by MISO (changed in both replicates) or DiffSplice were considered. This identified 181, 231 and 232 cassette exons that were regulated by ACINUS, RNPS1 and PININ, respectively (Supplementary Fig. 3a, Table 2). Of these, 40% and 29% are significantly changed in both KDs for ACINUS and PININ, respectively (Supplementary Fig. 3), indicating that ASAP and PSAP complexes can regulate different genes on their own. Only a small portion is also regulated by EJC core proteins, in agreement with previous study showing that most of the EJC core-dependent splicing events are not regulated by ACINUS^7^. ACINUS knockdown also caused splicing changes in both direction (exon inclusion and exon skipping) in contrary with previous *in vitro* studies^23^, indicating that *in vivo*, ACINUS can regulate splicing in various ways, as recently observed^22^.

**Figure 2:**
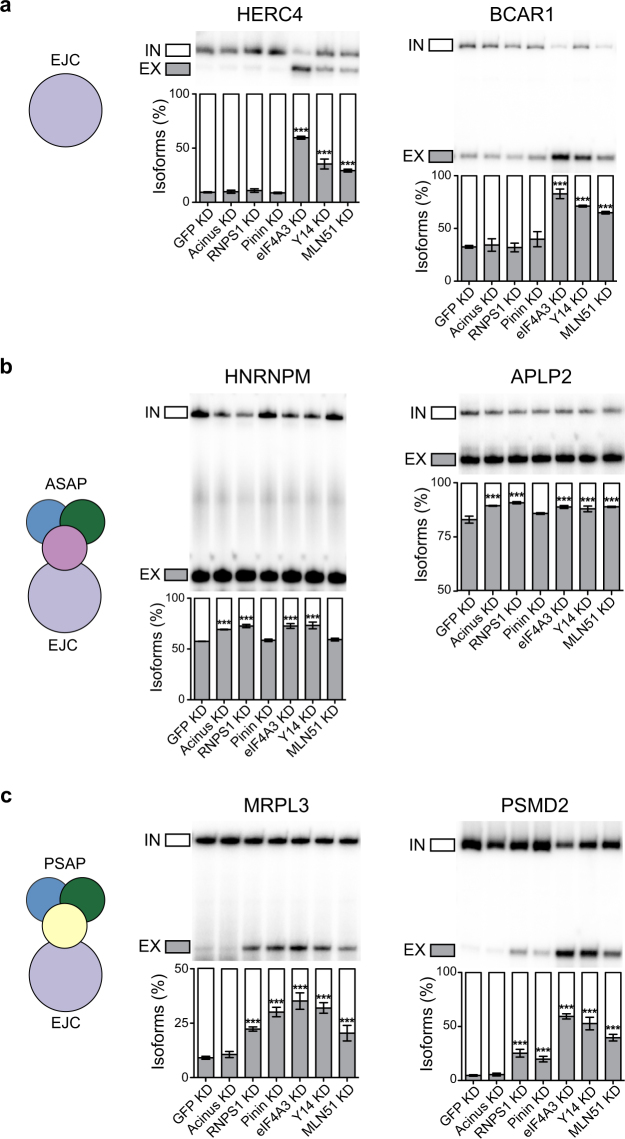
The splicing changes are dependent on different components. RT-PCR validation of alternative splicing events depending on only EJC (**a**), ASAP and EJC (**b**) or PSAP and EJC (**c**). Quantifications of triplicate experiments are shown as mean ± SD. *p < 0.05; **p < 0.01; ***p < 0.001, one-way ANOVA.

We focused our attention on splicing events that are potentially EJC-dependent and also PSAP- or ASAP-dependent or not. Using RT-PCR, we have validated and quantified 18 predicted EJC-dependent splicing changes, most of those corresponding to cassette exons (Fig. 2, Supplementary Fig. 4). Three distinct classes of splicing events could be distinguished. The first class corresponds to events depending on EJC core proteins but not on ASAP or PSAP complex components (HERC4, BCAR1, HNRNDL, SRSF2, GLRX3 and SPATA5L1; Fig. 2a, Supplementary Fig. 4). The second class corresponds to splicing events both EJC- and ASAP-dependent (HNRNPM and APLP2, Fig. 2b). The third class corresponds to splicing events both EJC- and PSAP-dependent (MRPL3, PSMD2, C20orf7, KPNA1, TPCN1, SMARD1, SACM1L, DDX27, ROBO1, AARSD1; Fig. 2c, Supplementary Fig. 4). The fact that we detected and validated much more splicing events from the third class than from the second class suggests that at least in HeLa cells, EJC cores are more commonly associated with PSAP than ASAP complex. For splicing events that are co-regulated by the EJC and the ASAP or the PSAP complexes, the ASAP or PSAP components most likely regulate splicing as part of the EJC complex. However, given that ASAP and PSAP complexes also exist as independent splicing regulators, we cannot exclude that the three complexes act independently. Moreover, the observation of EJC-dependent splicing events also requiring the ASAP or the PSAP complex strongly suggest that these two complexes bind the EJC core in a mutually exclusive manner and regulate different splicing events.

### PININ and ACINUS are functionally distinct as EJC peripheral components

**Figure 3:**
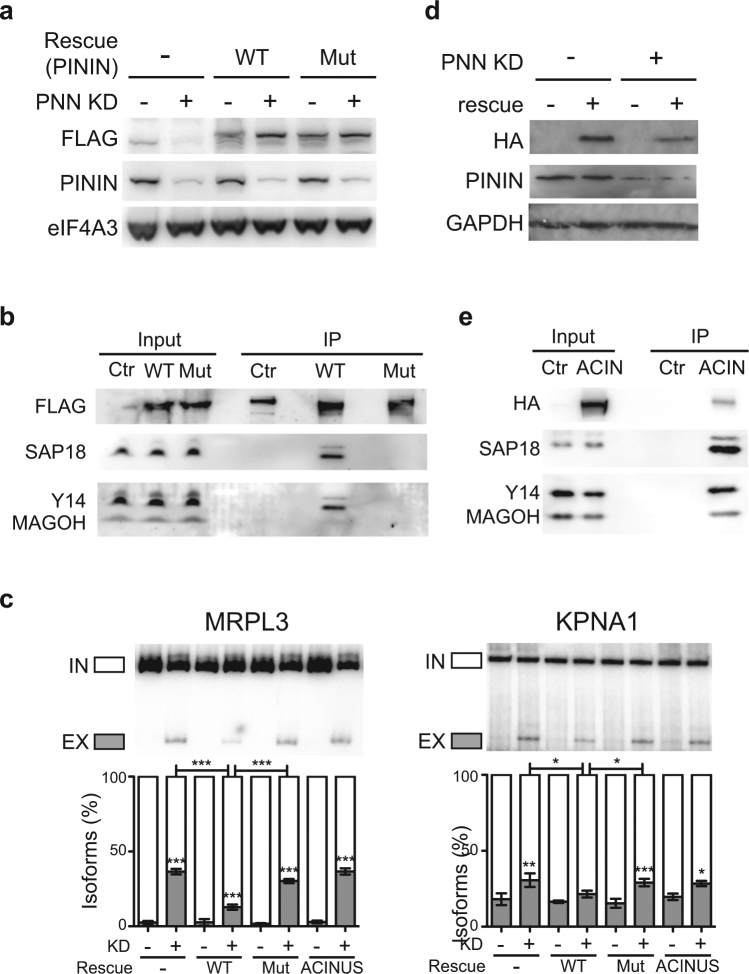
PININ and ACINUS are functionally distinct as EJC components. Western blot showing the expression level of siRNA-resistant FLAG-PININ (WT), FLAG-PININ-Mut (**a**) or HA-ACINUS (**d**) in the background of control and endogenous PININ KD. GAPDH is shown as a loading control. Immunoprecipitation of FLAG-PININ and FLAG-PININ-Mut (**b**) or HA-ACINUS (**e**) expressed in HeLa cells. (**c**) RT-PCR detection of MRPL3 and KPNA1 alternative splicing events in control and PININ KD cells expressing FLAG-PININ, FLAG-PININ-Mut or HA-ACINUS. Quantifications of triplicate experiments are shown below as mean ± SD. *p < 0.05; **p < 0.01; ***p < 0.001, one-way ANOVA.

Since ASAP and PSAP complexes together with EJC core can regulate different splicing events, we asked whether they are interchangeable *in vivo*. We focused on PININ-dependent events as they are more abundant. We performed rescue experiments with a siRNA-resistant form of FLAG-PININ (FLAG-PININ-WT) in the background of PININ KD cells, as well as a mutant lacking the RSB domain that interact with RNPS1 and SAP18^24^ (delta aa 237–247, FLAG-PININ-Mut) (Fig. 3a). Since RSB domain is important for forming the PSAP complex, deletion mutant should not form the PSAP complex. Co-immunoprecipitations showed that the FLAG-PININ-WT can be incorporated into PSAP complex and can pull down both SAP18 and Y14 (Fig. 3b). FLAG-PININ-Mut, however, failed to interact with SAP18 and Y14 (Fig. 3b), indicating that integrity of PSAP complex *in vivo* is required to interact with EJC core. Splicing validation showed that PININ KD caused expected splicing changes and that FLAG-PININ-WT can partially rescue the effect whereas FLAG-PININ-Mut cannot (Fig. 3c, Supplementary Fig. 5). Therefore, an assembled PSAP complex is required for the splicing regulation. To assess whether the ASAP-containing EJCs can replace PSAP-containing EJCs when the amount of PSAP is low, in parallel, we overexpressed HA-ACINUS in the background of PININ KD cells (Fig. 3d). HA-ACINUS can be incorporated into the ASAP complex and coprecipitate eIF4A3 (Fig. 3e). HA-ACINUS, however, did not rescue the splicing changes caused by PININ KD (Fig. 3c, Supplementary Fig. 5), indicating that ASAP-containing EJCs cannot replace the PSAP-containing EJCs in regulating these specific splicing events *in vivo*.

In summary, we have found that EJC peripheral factors PSAP and ASAP complexes can regulate different splicing events but that they also can, in certain cases, act together with the EJC core to modulate splicing choices. In addition, we found that the integrity of the PSAP complex is necessary for splicing regulation, which suggest that for at least some splicing events, an EJC composed of the EJC core and either the PSAP or the ASAP complexes is the entity that regulates these splicing events. In these cases, the fully assembled EJC complex could regulate the splicing of flanking introns as observed in the case for *piwi* mRNA in drosophila^5,6^. However, we cannot exclude some other indirect mechanisms such as interference with transcription rate as previously observed in human cells^7^. In the case of splicing events depending on the EJC core but not on the ASAP or PSAP complexes, we can suppose that other splicing factors associated with the EJC core participate to splicing regulation. SR proteins are good candidates as they are known to bind to EJCs^18,19^.

We have identified only a small number of splicing events depending on the EJC core and even less events, requiring both the EJC core and the ASAP or the PSAP complexes. Whether such small proportions reflect experimental limitations or the reality remains to be determined. However, this study provides the first evidence that EJCs can be functionally distinct and so, have different functional flavours shedding new light on the complexity of EJCs and more generally of mRNPs. Such functional diversity could explain why the excision of different introns have different EJC-related consequences on mRNA destiny as in the case of *oskar* for which only splicing of the first intron has consequence on mRNA localization^8,28^. Even though this study concentrates on splicing regulators, it is perceivable that different EJC complexes exist to regulate other EJC functions such as translation activation and NMD implying that the simple presence of an EJC is not inevitably associated to its known generic functions. Future work should clarify the mechanisms dictating EJC composition and extend the role of EJC diversity to finely tune post-transcriptional gene expression.

## Methods

### Antibodies and plasmids

Rabbit polyclonal anti-eIF4A3, anti-Y14, and anti-MLN51 are gifts from C. Tomasetto. Rabbit polyclonal anti-Acinus is from Bethyl (A300-999A) and goat polyclonal Pinin is from Santa Cruz. Rabbit polyclonal SAP18 is from Santa Cruz Biotechnology (sc-25377). Rabbit polyclonal GAPDH is from Cell Signalling.

Plasmids for recombinant protein expression were previously described^3,24^.

The original p3xFLAG-CMV-eIF4A3 was a gift from M. Moore. p3XFLAG-CMV-PininWT (siRNA-resistant) was created by site-directed mutagenesis (Invitrogen) for regions targeted by siRNA Pinin. The mutant Pinin with RBS deletion (aa 237–246) was generated from the p3xFLAG-CMV-PininWT. The HA-Acinus was generated by cloning the Acinus S’ cDNA into pcDNA 3.1 vector.

### Recombinant proteins and *in vitro* binding assays

TAP-RNPS1-His and TAP-ACINUS S’-His were expressed in Hi5 insect cells as previously described^24^. The other recombinant proteins were expressed in E. coli as previously described^3,24^. Interaction assays were performed as previously described^3,24^.

### Cell culture and transfections

HeLa cells were maintained in DMEM (GIBCO) with 10% FCS (Life Technologies) and penicillin/streptomycin (Life Technologies). For siRNA knockdown, cells were transfected with 10 nM of siRNA using RNAiMax (Invitrogen) according to manufacturer’s protocol. Cells were harvested 48 h later. The following siRNA duplexes (Eurogentec) were used:

siGFP, 5′-UGAAUUAGAUGGCGAUGUU-3′;

siACINUS, 5′-GCUCGCUGCCCAAAUCAUU-3′;

siRNPS1, 5′-CCAAGGACAUUGAGGACGU-3′;

siPININ, 5′-AAUCCAGGUCGCGAUCGAA-3′.

For co-transfection of siRNA and plasmids, Lipofection 2000 (Invitrogen) was used. 10 nM of siRNA was mixed together with 1ug of p3xFLAG-CMV-PininWT, p3xFLAG-CMV-PininMut or HA-Acinus to obtain similar level of expression. Cells were collected 48 h after.

### mRNA-seq and data analysis

For mRNA-seq, polyA+ mRNAs were extracted from HeLa cells treated with siRNA against GFP, ACINUS, RNPS1, or PININ using Illumina TruSeq RNA sample preparation kit (Illumina) according to manufacturer’s protocol. The fragmented mRNAs were sequenced using Illumina Hi-Seq. 2000 single end sequencing with 51nt length.

### Mapping of reads to the human genome

Raw reads that do not pass the Illumina quality filter were firstly discarded. The remaining mRNA-seq reads were mapped to the human genome (hg19) using TopHat v.2.08^29^. Ensembl75 annotations were provided to TopHat (−G option). Alignments with reads that matches multiple positions on the genome were removed.

### Analysis of differential gene expression

From the mapped reads and the GTF annotation files (Ensembl75), we counted the number of reads for each gene using htseq-count. To normalise the read counts and perform differential gene expression analysis, we used the DESeq package^30^. We chose a read coverage of 10 as a threshold in any of the KD conditions to ensure transcripts are reasonably expressed. The coverage was computed as the read length (51 bases) multiplied by the number of reads divided by the transcript length. This filter selected 7701 genes. Then we filtered transcripts with significant expression changes using the cut-off of adjusted p value 0.05.

### Identification of differential exon usage

We use two different software to perform differential exon usage analysis: MISO^27^ and DiffSplice^26^. For MISO, we run each replicate as separate experiments, and only considered events that are present in both replicates. The following parameter was used: ∆Ψ > 0.1, bayes-factor > 5, number of skipping reads >10, number of inclusion reads >10, sum of inclusion and skipping reads >20. For DiffSplice, the following parameters were used: 5 for thresh_average_read_coverage_exon and 10 for thresh_average_read_coverage_intron. The thresholds for splice junction filtering were increased to 5 and thresh_junction_filter_num_samples_presence was set to 1. For the differential tests, the minimum value square root of JSD for significant differential transcription (thresh_sqrtJSD) was set to 0.1 and the false discovery rate was set to 1.

### RNA extraction and RT-PCR analysis

Total RNA was extracted from cells using TRI reagent (Ambion) according to manufacturer’s protocol. The RNA was digested with 2U RNase-free DNase I (Ambion) for 30 min at 37 °C before phenol extraction and precipitation. Reverse transcription was performed using 500 ng RNA with random primers and RevertAid reverse transcriptase (Fermentas) according manufacturer’s protocol. For radioactive PCR analysis of alternative splicing, primer sets were designed across the constitutive exons. The list of primer sequences is shown in Additional file 8. To radioactively label the primers, 2.5 µM of each primer were incubated with ^32^P-γ-ATP and PNK (Fermentas) at 37 °C for 30 min before purifying through a G6 column (Biorad). PCR reaction was performed with DreamTaq polymerase (Fermentas) using 1 µl of hot primers and 1 µl of cDNA for 25 cycles at 60 °C annealing temperature. The 2x RNA Loading Dye (Ambion) were added to the PCR products, and were denatured at 95 °C before resolving on the 8% denaturing polyacrylamide gel. Results were quantified with the Typhoon Imager (GE healthcare) and ImageJ software.

### Quantitative real-time PCR

Real-time PCR was performed using SYBR Select Master Mix (Life Technologies) on LightCycler (Roch). Primers for qPCR can be found in Additional file 14. The relatively amount of each RNA was calculated by the threshold cycle for each PCR product (Ct) in control and knockdown conditions, and compared with the house-keeping gene *GAPDH* (Glyceraldehyde-3-phosphate dehydrogenase).

### Immunoprecipitation and western blot analysis

HeLa cells were transfected with empty vectors or FLAG-tagged PININ for 48 h before collection. The cells were lysed in PXL buffer (1xPBS, 0.1% SDS, 0.5% NP-40, 0.5% Na deoxycholate) supplemented with protease inhibitor (Calbiochem), and incubated with RNAse A (Fermentas) and Turbo DNase (Ambion) for 10 min at 37 °C. After centrifugation, the cell lysates were incubated with anit-FLAG beads (Sigma) for 2 h. After washing three times with 10 mM Tris-HCL, pH7.5, 150 mM NaCl, 2.5 mM MgCl_2_, 1% NP-40, the proteins were eluted by adding 1x elution buffer and heating at 50 °C. The eluate was subjected to western blot analysis, and 1/15 of input was used.

For western blot analysis, cells were lysed in PXL buffer as described above. Equal amounts of total protein were used, and separated by 6%, 10% or 12% of SDS-PAGE depending on the protein probed. The proteins were transferred to nitrocellulose membrane (Amersham), and blocked in 5% milk. The membrane was incubated with primary antibody (1:1000 dilution except for Pinin, 1:500 dilution) for 2 h at room temperature before adding peroxidase-conjugated secondary antibodies (Thermo Scientific) for 1 h. The antibodies were detected by Femto ELC (Thermo Scientific) on a LAS400 machine (GE healthcare).

## Electronic supplementary material

Supplementary info
