# Supplementary material for: Exon Junction Complexes can have distinct functional flavours to regulate specific splicing events
Source: Sci Rep. 2018 Jun 22;8:9509. doi: 10.1038/s41598-018-27826-y (PMC6015020; doi:10.1038/s41598-018-27826-y)
Supplement: Supplementary file 1 — Supplementary info [file 41598_2018_27826_MOESM1_ESM.pdf]

Exon Junction Complexes can have distinct functional flavours to regulate specific splicing events

Zhen Wang, Lionel Ballut, Isabelle Barbosa, Hervé Le Hir

Supplementary Figure Legends:

Supplementary Figure 1 RNPS1 and SAP18 do not interfere with ACINUS Binding to the EJC core. Co-precipitations with TAP-ACINUS mixed with no protein (lane 2) or with eIF4AIII, MLN51-S and MAGOH/Y14 with ADNP and 47-mer ssRNA and this, in the absence (lane 1) or in the presence of RNPS1 and SAP18 (lane 3). Protein mixtures before (input, 20% of total) or after precipitation (precipitate) were separated on 10%(w/v) acrylamide SDS-PAGE. The asterisk indicates degraded products also observed in the control lane 2.

Supplementary Figure 2 mRNAseq of EJC depleted cells.
a) Western blot of HeLa cells with siRNA of different ASAP components. GAPDH is used as a control. b) qPCR quantification of the KD efficiency. The expression level is normalised against GAPDH gene. c) Pie chart for the uniquely mapped mRNAseq read in different KD conditions used in this study. d) Correlation between the two replicate experiments for each KD condition.

Supplementary Figure 3: Venn diagram of splicing changes identified for each KD.
a) Venn Diagram showing the overlap between significantly changed cassette exons in the two replicates identified by MISO. b) Cassette exons that have been identified to be significantly increased (left) or decreased (right) upon each gene KD identified by MISO and Diffsplice. c) Intron retention events that are significantly increased (left) or decreased (right) upon gene KD. d) Overlapping of significantly changed cassette exons between EJC KD, ASAP KD and PSAP KD.

Supplementary Figure 4: EJC-dependent splicing events that are both PSAP-dependent and independent.
RT-PCR validation of cassette exon events that were EJC-dependent. Those exons can be both PSAP- and EJC-dependent (A) or EJC-dependent only (B). The quantifications of triplicate experiments are shown below as mean ± SD. *p<0.05; **p<0.01; ***p<0.001, one-way ANOVA

Supplementary Figure 5: Cassette exon in rescue experiments.
RT-PCR validation of candidate alternative splicing events in control and Pinin KD cells overexpressed with siRNA-resistant Pinin (WT), a mutant that does not form EJC (Mut) or HA-Acinus. The quantifications of triplicate experiments are shown below as mean ± SD. *p<0.05; **p<0.01; ***p<0.001, one-way ANOVA.

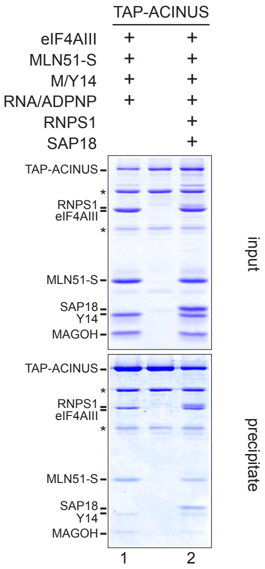

Supplementary Figure 1

Supplementary Figure 2

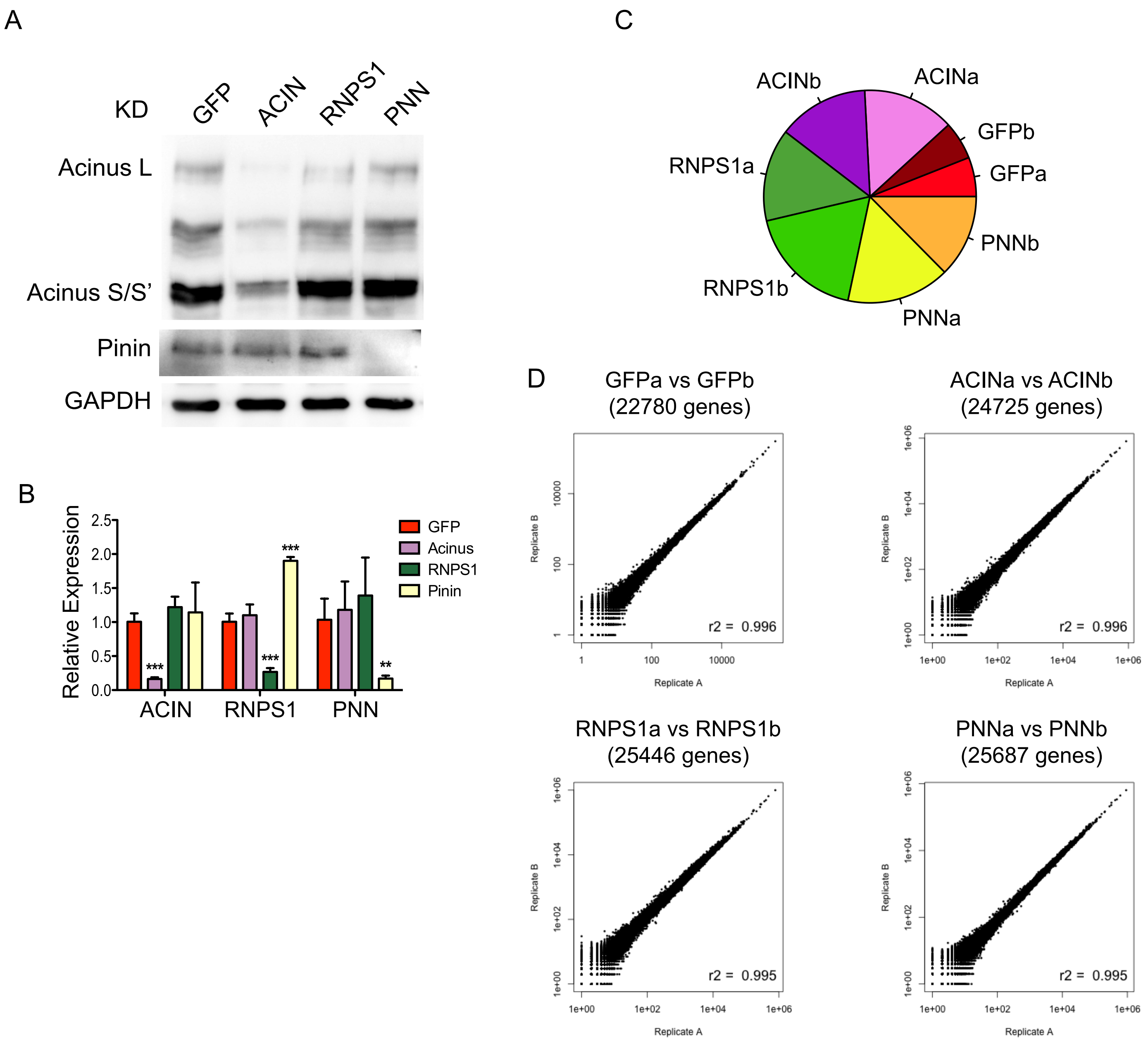

Supplementary Figure 3

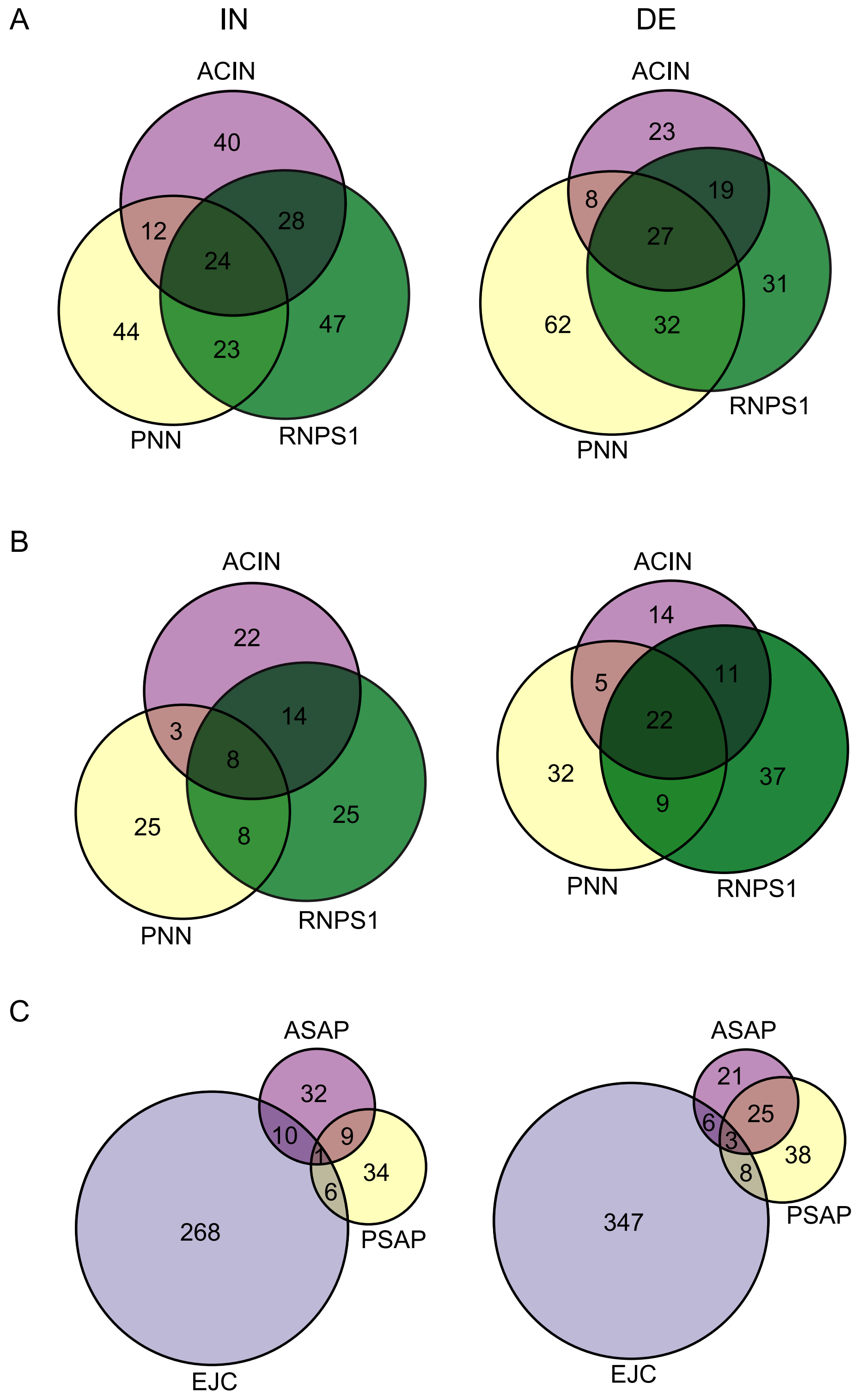

Supplementary Figure 4

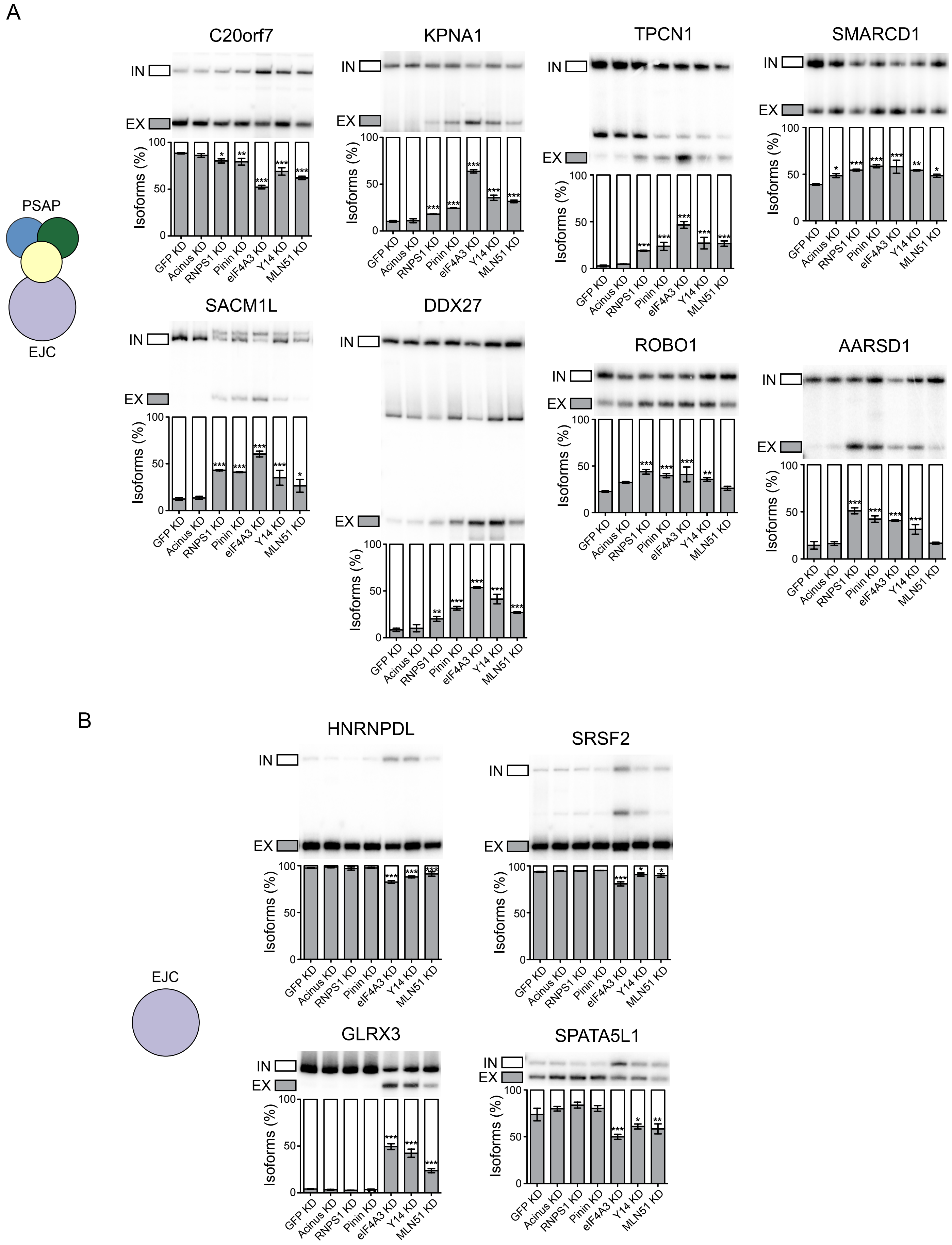

Supplementary Figure 5

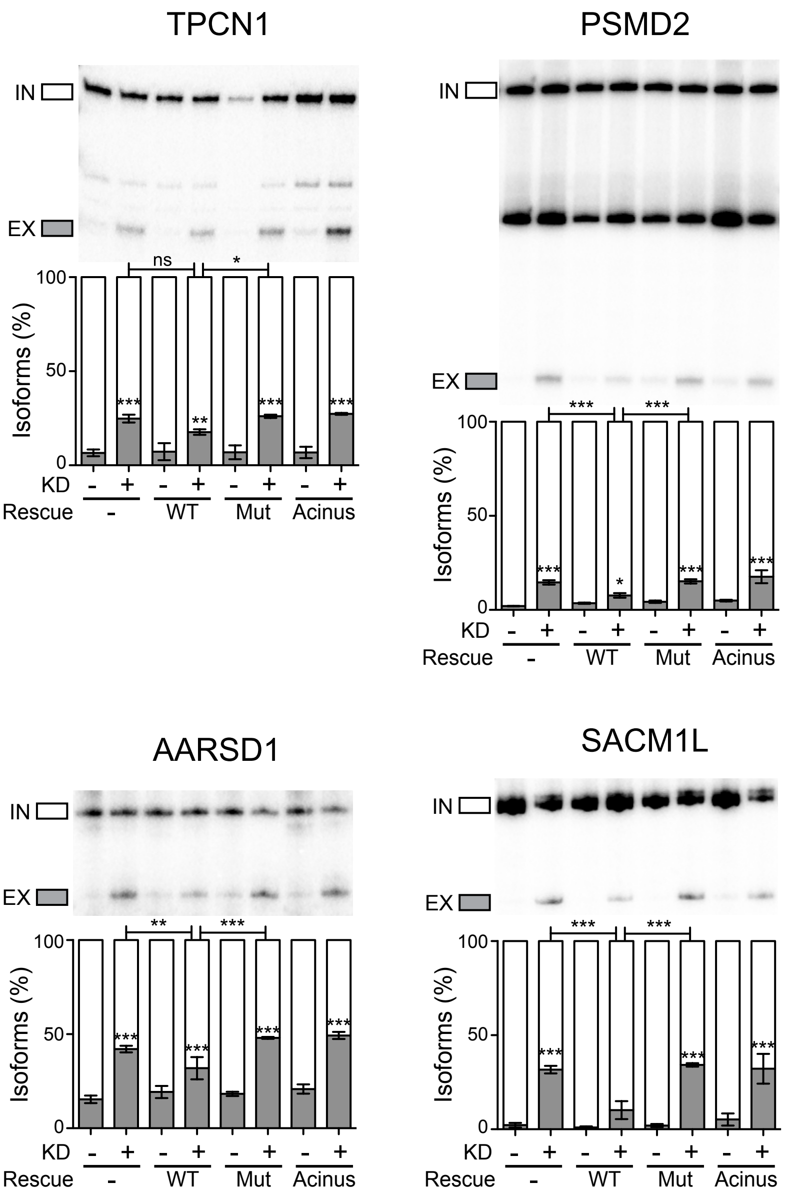

Supplementary Table 1. Mapping Information for mRNAseq data.

Dataset	Raw reads	Quality filtered reads	Tophat unique reads
GFPa	18046126	15871594 (88%)	14854728 (93.6%)
GFPb	17353993	15262567 (87.9%)	14373121 (94.2%)
ACINa	42899256	36482548 (85.0%)	34780298 (95.3%)
ACINb	42452405	35939736 (84.7%)	34261174 (95.3%)
RNPS1a	43474148	36739604 (84.5%)	34932944 (95.1%)
RNPS1b	55617537	46820958 (84.2%)	44622497 (95.3%)
PNNa	48420650	41054871 (84.8%)	39046469 (95.1%)
PNNb	38439995	32792300 (85.3%)	31233937 (95.2%)

Supplementary Table 2. List of cassette exon events identified by MISO and DiffSplice.

event	miso_chr	miso_start	miso_stop	misoGFP	misoACIN	misoRNPS1
chr11:130005610:130010293	chr11	130005458	130010378	0,335	0,195	0,155
chr11:35211519:35236399	chr11	35211382	35236461	0,165	NA	0,31
chr11:35211612:35229652	chr11	35211382	35229753	0,04	NA	0,15
chr11:57529518:57561482	chr11	57529234	57561553	0,83	NA	0,595
chr12:56554104:56555171	chr12	56554027	56555366	0,39	0,235	NA
chr14:21702388:21737457	chr14	21702112	21737638	0,84	NA	0,735
chr14:23535217:23538685	chr14	23535175	23538826	0,1	0,245	NA
chr17:16251999:16253245	chr17	16251930	16253343	0,46	NA	NA
chr17:16342728:16343499	chr17	16342641	16343567	0,405	NA	NA
chr17:16342728:16343499	chr17	16342641	16343567	0,41	NA	0,515
chr17:79977257:79977734	chr17	79977169	79977785	0,625	0,365	NA
chr19:11031803:11032291	chr19	11031726	11033448	0,265	NA	NA
chr19:1358463:1360135	chr19	1358393	1361031	0,63	NA	0,28
chr19:50413141:50432583	chr19	50410084	50432988	0,38	NA	NA
chr19:50413141:50432583	chr19	50410084	50432988	0,345	NA	NA
chr1:207959027:207966864	chr1	207958964	207968861	0,375	NA	0,525
chr1:234534299:234536927	chr1	234534128	234537008	0,145	NA	0,46
chr1:46072263:46078841	chr1	46072154	46078920	0,21	NA	NA
chr1:54722859:54747111	chr1	54722800	54747200	0,28	0,625	NA
chr1:89237562:89251787	chr1	89237346	89251896	0,93	NA	NA
chr20:34326939:34328447	chr20	34326890	34328519	0,305	0,605	NA
chr21:27354790:27394156	chr21	27354657	27394358	0,91	NA	NA
chr21:44317157:44328974	chr21	44317037	44329773	0,69	0,53	NA
chr22:29925228:29927820	chr22	29925151	29927952	0,15	NA	0,425
chr3:172473164:172474773	chr3	172473085	172474955	0,84	NA	0,55
chr3:183962509:183963256	chr3	183962389	183963403	0,985	NA	0,86
chr3:186502485:186503672	chr3	186502353	186503840	0,78	0,655	NA
chr3:186505671:186506914	chr3	186505592	186507685	0,565	0,76	NA
chr3:186505671:186506914	chr3	186505592	186507685	0,52	0,715	NA
chr3:33437772:33438537	chr3	33437654	33438627	0,145	NA	0,325
chr3:47852201:47859512	chr3	47852147	47859607	0,63	NA	NA
chr5:179044912:179047893	chr5	179044800	179048036	0,885	NA	0,5
chr5:304336:311408	chr5	304292	311517	0,94	NA	NA
chr5:304336:311408	chr5	304292	311517	0,955	NA	NA
chr5:70900295:70927948	chr5	70900183	70928012	0,785	NA	NA
chr6:30675231:30679189	chr6	30675161	30679281	0,68	NA	0,305
chr6:31611971:31612302	chr6	31611859	31612379	0,285	NA	0,515
chr7:134618141:134625843	chr7	134617739	134625988	0,15	NA	NA
chr7:23561459:23571408	chr7	23561326	23571656	0,285	0,535	0,6
chr7:23561459:23571408	chr7	23561326	23571656	0,27	NA	0,59
chr7:6431672:6439757	chr7	6431555	6439819	0,18	0,06	NA
chr9:131030803:131036129	chr9	131030699	131036251	0,42	0,165	NA
chr9:131353904:131356454	chr9	131353756	131356652	0,97	NA	NA
chr9:34635855:34637217	chr9	34634719	34637417	0,73	0,535	NA
chrX:119565317:119575585	chrX	119560003	119575749	0,46	0,78	0,7
chr10:104828479:104835843	NA	NA	NA	NA	NA	NA
chr10:104861083:104865463	NA	NA	NA	NA	NA	NA
chr10:120920481:120921852	NA	NA	NA	NA	NA	NA
chr10:126449072:126453961	NA	NA	NA	NA	NA	NA
chr10:129911866:129914755	NA	NA	NA	NA	NA	NA
chr10:27040712:27047991	NA	NA	NA	NA	NA	NA
chr10:27815818:27822857	NA	NA	NA	NA	NA	NA
chr10:32120728:32128565	NA	NA	NA	NA	NA	NA
chr10:34690845:34759013	NA	NA	NA	NA	NA	NA
chr10:35468204:35495823	NA	NA	NA	NA	NA	NA
chr10:70097090:70098260	NA	NA	NA	NA	NA	NA
chr10:75198178:75204483	NA	NA	NA	NA	NA	NA
chr10:75577312:75581440	NA	NA	NA	NA	NA	NA
chr10:88930731:88939832	NA	NA	NA	NA	NA	NA
chr10:95099921:95103613	NA	NA	NA	NA	NA	NA
chr10:95148911:95155899	NA	NA	NA	NA	NA	NA
chr10:97444381:97446252	NA	NA	NA	NA	NA	NA
chr11:102033302:102076624	NA	NA	NA	NA	NA	NA

chr11:102076805:102094353	NA	NA	NA	NA	NA	NA
chr11:10827557:10828802	NA	NA	NA	NA	NA	NA
chr11:118128028:118133217	NA	NA	NA	NA	NA	NA
chr11:12261132:12278332	NA	NA	NA	NA	NA	NA
chr11:122928536:122929475	NA	NA	NA	NA	NA	NA
chr11:33369559:33370068	NA	NA	NA	NA	NA	NA
chr11:36296322:36298639	NA	NA	NA	NA	NA	NA
chr11:47587538:47593023	NA	NA	NA	NA	NA	NA
chr11:502181:506609	NA	NA	NA	NA	NA	NA
chr11:62445057:62445399	NA	NA	NA	NA	NA	NA
chr11:64532990:64534664	NA	NA	NA	NA	NA	NA
chr11:66612708:66613331	NA	NA	NA	NA	NA	NA
chr11:71721900:71723941	NA	NA	NA	NA	NA	NA
chr11:72983511:73007530	NA	NA	NA	NA	NA	NA
chr11:82985783:82991184	NA	NA	NA	NA	NA	NA
chr11:85685855:85692172	NA	NA	NA	NA	NA	NA
chr11:85687725:85692172	NA	NA	NA	NA	NA	NA
chr11:8933218:8936373	NA	NA	NA	NA	NA	NA
chr11:95598840:95621320	NA	NA	NA	NA	NA	NA
chr12:104680887:104705068	NA	NA	NA	NA	NA	NA
chr12:10856747:10865810	NA	NA	NA	NA	NA	NA
chr12:109526317:109530312	NA	NA	NA	NA	NA	NA
chr12:111160500:111168456	NA	NA	NA	NA	NA	NA
chr12:111890644:111893833	NA	NA	NA	NA	NA	NA
chr12:112304035:112306557	NA	NA	NA	NA	NA	NA
chr12:113716640:113722386	NA	NA	NA	NA	NA	NA
chr12:121461940:121469245	NA	NA	NA	NA	NA	NA
chr12:121683043:121687590	NA	NA	NA	NA	NA	NA
chr12:1219513:1225032	NA	NA	NA	NA	NA	NA
chr12:124824989:124826369	NA	NA	NA	NA	NA	NA
chr12:2973918:2975559	NA	NA	NA	NA	NA	NA
chr12:31236810:31255385	NA	NA	NA	NA	NA	NA
chr12:32890095:32892998	NA	NA	NA	NA	NA	NA
chr12:394828:401925	NA	NA	NA	NA	NA	NA
chr12:42768876:42781258	NA	NA	NA	NA	NA	NA
chr12:49065745:49073438	NA	NA	NA	NA	NA	NA
chr12:50480661:50482304	NA	NA	NA	NA	NA	NA
chr12:51442968:51450133	NA	NA	NA	NA	NA	NA
chr12:53898599:53899433	NA	NA	NA	NA	NA	NA
chr12:54741823:54744260	NA	NA	NA	NA	NA	NA
chr12:56362732:56364828	NA	NA	NA	NA	NA	NA
chr12:57065593:57066736	NA	NA	NA	NA	NA	NA
chr12:57501097:57501946	NA	NA	NA	NA	NA	NA
chr12:58112965:58114189	NA	NA	NA	NA	NA	NA
chr12:6495610:6497966	NA	NA	NA	NA	NA	NA
chr12:6690300:6700704	NA	NA	NA	NA	NA	NA
chr12:6780004:6782382	NA	NA	NA	NA	NA	NA
chr12:6787626:6788112	NA	NA	NA	NA	NA	NA
chr12:6833984:6837389	NA	NA	NA	NA	NA	NA
chr12:7084310:7084858	NA	NA	NA	NA	NA	NA
chr12:7354437:7355208	NA	NA	NA	NA	NA	NA
chr12:95645847:95650926	NA	NA	NA	NA	NA	NA
chr12:987527:990858	NA	NA	NA	NA	NA	NA
chr13:114193822:114202618	NA	NA	NA	NA	NA	NA
chr13:114291015:114294435	NA	NA	NA	NA	NA	NA
chr13:115057267:115067206	NA	NA	NA	NA	NA	NA
chr13:37563692:37569126	NA	NA	NA	NA	NA	NA
chr14:102695994:102698872	NA	NA	NA	NA	NA	NA
chr14:105181193:105185132	NA	NA	NA	NA	NA	NA
chr14:105911848:105916395	NA	NA	NA	NA	NA	NA
chr14:21460364:21465044	NA	NA	NA	NA	NA	NA
chr14:23396823:23397706	NA	NA	NA	NA	NA	NA
chr14:56128330:56133959	NA	NA	NA	NA	NA	NA
chr14:65560533:65569022	NA	NA	NA	NA	NA	NA
chr14:89063152:89073587	NA	NA	NA	NA	NA	NA
chr14:93170592:93182530	NA	NA	NA	NA	NA	NA

chr14:96991728:96994290	NA	NA	NA	NA	NA	NA
chr15:101866684:101872057	NA	NA	NA	NA	NA	NA
chr15:41102955:41105536	NA	NA	NA	NA	NA	NA
chr15:66786890:66789980	NA	NA	NA	NA	NA	NA
chr15:69629840:69672222	NA	NA	NA	NA	NA	NA
chr15:72542433:72543548	NA	NA	NA	NA	NA	NA
chr15:76206546:76225026	NA	NA	NA	NA	NA	NA
chr15:91510432:91512677	NA	NA	NA	NA	NA	NA
chr16:15180115:15185172	NA	NA	NA	NA	NA	NA
chr16:2809662:2810302	NA	NA	NA	NA	NA	NA
chr16:29842394:29851724	NA	NA	NA	NA	NA	NA
chr16:29982899:29984271	NA	NA	NA	NA	NA	NA
chr16:46836982:46858298	NA	NA	NA	NA	NA	NA
chr16:47117712:47143394	NA	NA	NA	NA	NA	NA
chr16:4873908:4882775	NA	NA	NA	NA	NA	NA
chr16:53501018:53515626	NA	NA	NA	NA	NA	NA
chr16:54954239:54957497	NA	NA	NA	NA	NA	NA
chr16:57792821:57793640	NA	NA	NA	NA	NA	NA
chr16:68380183:68386151	NA	NA	NA	NA	NA	NA
chr16:69154552:69155339	NA	NA	NA	NA	NA	NA
chr16:70582345:70588349	NA	NA	NA	NA	NA	NA
chr16:71799487:71803526	NA	NA	NA	NA	NA	NA
chr16:720557:720892	NA	NA	NA	NA	NA	NA
chr16:87760530:87782279	NA	NA	NA	NA	NA	NA
chr16:87788898:87795555	NA	NA	NA	NA	NA	NA
chr16:88653092:88664586	NA	NA	NA	NA	NA	NA
chr16:88783619:88785982	NA	NA	NA	NA	NA	NA
chr16:89818630:89828357	NA	NA	NA	NA	NA	NA
chr17:15989756:15995177	NA	NA	NA	NA	NA	NA
chr17:16052831:16055260	NA	NA	NA	NA	NA	NA
chr17:17046079:17049350	NA	NA	NA	NA	NA	NA
chr17:18148559:18152441	NA	NA	NA	NA	NA	NA
chr17:18152035:18152441	NA	NA	NA	NA	NA	NA
chr17:20906155:20924519	NA	NA	NA	NA	NA	NA
chr17:27409456:27413456	NA	NA	NA	NA	NA	NA
chr17:2861088:2866724	NA	NA	NA	NA	NA	NA
chr17:29576137:29585362	NA	NA	NA	NA	NA	NA
chr17:30678902:30687746	NA	NA	NA	NA	NA	NA
chr17:30692506:30694791	NA	NA	NA	NA	NA	NA
chr17:35564714:35578645	NA	NA	NA	NA	NA	NA
chr17:41103911:41106893	NA	NA	NA	NA	NA	NA
chr17:41107989:41108421	NA	NA	NA	NA	NA	NA
chr17:46133960:46134706	NA	NA	NA	NA	NA	NA
chr17:47482404:47486497	NA	NA	NA	NA	NA	NA
chr17:48156290:48167554	NA	NA	NA	NA	NA	NA
chr17:5326149:5329556	NA	NA	NA	NA	NA	NA
chr17:5326149:5335862	NA	NA	NA	NA	NA	NA
chr17:5329619:5335862	NA	NA	NA	NA	NA	NA
chr17:62496891:62498128	NA	NA	NA	NA	NA	NA
chr17:65871136:65887960	NA	NA	NA	NA	NA	NA
chr17:66247319:66252955	NA	NA	NA	NA	NA	NA
chr17:71205907:71223304	NA	NA	NA	NA	NA	NA
chr17:7123845:7124264	NA	NA	NA	NA	NA	NA
chr17:74085401:74090495	NA	NA	NA	NA	NA	NA
chr17:7490064:7491413	NA	NA	NA	NA	NA	NA
chr17:74922812:74934070	NA	NA	NA	NA	NA	NA
chr17:75398577:75483564	NA	NA	NA	NA	NA	NA
chr17:7810806:7811212	NA	NA	NA	NA	NA	NA
chr17:79478604:79478953	NA	NA	NA	NA	NA	NA
chr17:79891252:79892802	NA	NA	NA	NA	NA	NA
chr17:8473130:8480554	NA	NA	NA	NA	NA	NA
chr18:675402:683246	NA	NA	NA	NA	NA	NA
chr19:10381902:10394805	NA	NA	NA	NA	NA	NA
chr19:1251900:1254166	NA	NA	NA	NA	NA	NA
chr19:13220652:13220972	NA	NA	NA	NA	NA	NA
chr19:14501891:14508470	NA	NA	NA	NA	NA	NA

chr19:1627425:1646354	NA	NA	NA	NA	NA	NA
chr19:17338008:17339065	NA	NA	NA	NA	NA	NA
chr19:18541740:18542457	NA	NA	NA	NA	NA	NA
chr19:19307855:19308330	NA	NA	NA	NA	NA	NA
chr19:30101364:30103024	NA	NA	NA	NA	NA	NA
chr19:34699956:34706501	NA	NA	NA	NA	NA	NA
chr19:3546166:3547270	NA	NA	NA	NA	NA	NA
chr19:36122307:36124000	NA	NA	NA	NA	NA	NA
chr19:41120352:41125252	NA	NA	NA	NA	NA	NA
chr19:41125398:41129509	NA	NA	NA	NA	NA	NA
chr19:41938313:41939442	NA	NA	NA	NA	NA	NA
chr19:41939578:41944123	NA	NA	NA	NA	NA	NA
chr19:45161178:45164559	NA	NA	NA	NA	NA	NA
chr19:47111584:47112181	NA	NA	NA	NA	NA	NA
chr19:50168103:50168929	NA	NA	NA	NA	NA	NA
chr19:51848500:51856407	NA	NA	NA	NA	NA	NA
chr19:5218543:5219321	NA	NA	NA	NA	NA	NA
chr19:55966697:55967716	NA	NA	NA	NA	NA	NA
chr19:6222695:6230581	NA	NA	NA	NA	NA	NA
chr19:805187:806408	NA	NA	NA	NA	NA	NA
chr1:117146647:117150564	NA	NA	NA	NA	NA	NA
chr1:12027148:12035340	NA	NA	NA	NA	NA	NA
chr1:148561115:148568736	NA	NA	NA	NA	NA	NA
chr1:150201477:150208081	NA	NA	NA	NA	NA	NA
chr1:150464965:150470006	NA	NA	NA	NA	NA	NA
chr1:151733375:151734581	NA	NA	NA	NA	NA	NA
chr1:154185100:154186933	NA	NA	NA	NA	NA	NA
chr1:154187050:154192818	NA	NA	NA	NA	NA	NA
chr1:154241430:154242676	NA	NA	NA	NA	NA	NA
chr1:155108852:155110037	NA	NA	NA	NA	NA	NA
chr1:155230450:155231877	NA	NA	NA	NA	NA	NA
chr1:15752514:15755089	NA	NA	NA	NA	NA	NA
chr1:160186698:160192446	NA	NA	NA	NA	NA	NA
chr1:162560301:162567582	NA	NA	NA	NA	NA	NA
chr1:164781386:164790774	NA	NA	NA	NA	NA	NA
chr1:167742605:167757057	NA	NA	NA	NA	NA	NA
chr1:176104222:176118142	NA	NA	NA	NA	NA	NA
chr1:181019422:181022709	NA	NA	NA	NA	NA	NA
chr1:184023997:184041961	NA	NA	NA	NA	NA	NA
chr1:201453186:201458001	NA	NA	NA	NA	NA	NA
chr1:211477482:211486062	NA	NA	NA	NA	NA	NA
chr1:225594534:225599039	NA	NA	NA	NA	NA	NA
chr1:225688772:225695653	NA	NA	NA	NA	NA	NA
chr1:228296019:228296850	NA	NA	NA	NA	NA	NA
chr1:2328659:2332296	NA	NA	NA	NA	NA	NA
chr1:23357127:23376880	NA	NA	NA	NA	NA	NA
chr1:241761299:241803184	NA	NA	NA	NA	NA	NA
chr1:242013888:242016660	NA	NA	NA	NA	NA	NA
chr1:24294213:24298063	NA	NA	NA	NA	NA	NA
chr1:24989295:24993306	NA	NA	NA	NA	NA	NA
chr1:36639079:36641800	NA	NA	NA	NA	NA	NA
chr1:37959415:37967405	NA	NA	NA	NA	NA	NA
chr1:37961519:37967405	NA	NA	NA	NA	NA	NA
chr1:38450451:38455220	NA	NA	NA	NA	NA	NA
chr1:39945681:39950273	NA	NA	NA	NA	NA	NA
chr1:41471766:41474331	NA	NA	NA	NA	NA	NA
chr1:42664901:42693554	NA	NA	NA	NA	NA	NA
chr1:47152542:47155259	NA	NA	NA	NA	NA	NA
chr1:52215867:52226362	NA	NA	NA	NA	NA	NA
chr1:53720857:53723991	NA	NA	NA	NA	NA	NA
chr1:53737018:53741303	NA	NA	NA	NA	NA	NA
chr1:54254869:54262362	NA	NA	NA	NA	NA	NA
chr1:54412222:54417659	NA	NA	NA	NA	NA	NA
chr1:61872399:61920975	NA	NA	NA	NA	NA	NA
chr1:63009416:63018403	NA	NA	NA	NA	NA	NA
chr1:70694238:70696778	NA	NA	NA	NA	NA	NA

chr1:75172678:75175782	NA	NA	NA	NA	NA	NA
chr1:75190518:75198640	NA	NA	NA	NA	NA	NA
chr1:85031695:85036265	NA	NA	NA	NA	NA	NA
chr1:85724405:85725041	NA	NA	NA	NA	NA	NA
chr1:8617582:8684369	NA	NA	NA	NA	NA	NA
chr1:87329288:87346345	NA	NA	NA	NA	NA	NA
chr1:9796100:9801152	NA	NA	NA	NA	NA	NA
chr20:11899249:11900366	NA	NA	NA	NA	NA	NA
chr20:1435777:1438845	NA	NA	NA	NA	NA	NA
chr20:30149539:30156923	NA	NA	NA	NA	NA	NA
chr20:32661441:32663680	NA	NA	NA	NA	NA	NA
chr20:34219947:34220233	NA	NA	NA	NA	NA	NA
chr20:34326939:34328746	NA	NA	NA	NA	NA	NA
chr20:34785963:34800194	NA	NA	NA	NA	NA	NA
chr20:34800298:34806798	NA	NA	NA	NA	NA	NA
chr20:37055146:37059684	NA	NA	NA	NA	NA	NA
chr20:43249788:43251470	NA	NA	NA	NA	NA	NA
chr20:43251571:43257688	NA	NA	NA	NA	NA	NA
chr20:45839542:45848909	NA	NA	NA	NA	NA	NA
chr20:5564968:5573973	NA	NA	NA	NA	NA	NA
chr20:61299262:61299829	NA	NA	NA	NA	NA	NA
chr21:34900641:34903065	NA	NA	NA	NA	NA	NA
chr22:24379450:24384120	NA	NA	NA	NA	NA	NA
chr22:24936941:24943876	NA	NA	NA	NA	NA	NA
chr22:29668420:29674120	NA	NA	NA	NA	NA	NA
chr22:29684744:29688489	NA	NA	NA	NA	NA	NA
chr22:29724884:29726367	NA	NA	NA	NA	NA	NA
chr22:46751485:46752739	NA	NA	NA	NA	NA	NA
chr22:50895102:50897684	NA	NA	NA	NA	NA	NA
chr2:100010861:100015211	NA	NA	NA	NA	NA	NA
chr2:101565942:101584743	NA	NA	NA	NA	NA	NA
chr2:102476326:102481392	NA	NA	NA	NA	NA	NA
chr2:102486877:102490109	NA	NA	NA	NA	NA	NA
chr2:127808488:127816587	NA	NA	NA	NA	NA	NA
chr2:1687923:1695700	NA	NA	NA	NA	NA	NA
chr2:188368497:188418927	NA	NA	NA	NA	NA	NA
chr2:192265194:192267358	NA	NA	NA	NA	NA	NA
chr2:201284219:201305372	NA	NA	NA	NA	NA	NA
chr2:201724469:201725961	NA	NA	NA	NA	NA	NA
chr2:228205096:228211942	NA	NA	NA	NA	NA	NA
chr2:230664045:230723501	NA	NA	NA	NA	NA	NA
chr2:230724290:230744698	NA	NA	NA	NA	NA	NA
chr2:234181698:234183322	NA	NA	NA	NA	NA	NA
chr2:236817550:236877106	NA	NA	NA	NA	NA	NA
chr2:27549085:27550044	NA	NA	NA	NA	NA	NA
chr2:27995559:28002300	NA	NA	NA	NA	NA	NA
chr2:28521358:28561317	NA	NA	NA	NA	NA	NA
chr2:36583766:36668401	NA	NA	NA	NA	NA	NA
chr2:36749456:36771519	NA	NA	NA	NA	NA	NA
chr2:61408540:61411847	NA	NA	NA	NA	NA	NA
chr2:70515324:70520750	NA	NA	NA	NA	NA	NA
chr2:74759052:74759947	NA	NA	NA	NA	NA	NA
chr2:9547034:9548242	NA	NA	NA	NA	NA	NA
chr3:100211772:100274053	NA	NA	NA	NA	NA	NA
chr3:107497366:107510087	NA	NA	NA	NA	NA	NA
chr3:107766139:107769425	NA	NA	NA	NA	NA	NA
chr3:111639266:111658322	NA	NA	NA	NA	NA	NA
chr3:113440663:113442828	NA	NA	NA	NA	NA	NA
chr3:124732834:124738106	NA	NA	NA	NA	NA	NA
chr3:127294348:127294783	NA	NA	NA	NA	NA	NA
chr3:128890381:128890498	NA	NA	NA	NA	NA	NA
chr3:130678185:130682815	NA	NA	NA	NA	NA	NA
chr3:131208924:131219274	NA	NA	NA	NA	NA	NA
chr3:133305566:133306740	NA	NA	NA	NA	NA	NA
chr3:133305566:133307197	NA	NA	NA	NA	NA	NA
chr3:135969310:135980806	NA	NA	NA	NA	NA	NA

chr3:145794682:145796903	NA	NA	NA	NA	NA	NA
chr3:154858081:154861232	NA	NA	NA	NA	NA	NA
chr3:183558428:183562019	NA	NA	NA	NA	NA	NA
chr3:184035285:184035538	NA	NA	NA	NA	NA	NA
chr3:196876667:196921296	NA	NA	NA	NA	NA	NA
chr3:27446454:27453133	NA	NA	NA	NA	NA	NA
chr3:33444396:33450728	NA	NA	NA	NA	NA	NA
chr3:37125297:37136283	NA	NA	NA	NA	NA	NA
chr3:38167372:38168001	NA	NA	NA	NA	NA	NA
chr3:38170879:38173417	NA	NA	NA	NA	NA	NA
chr3:44970928:44974610	NA	NA	NA	NA	NA	NA
chr3:47958664:47963255	NA	NA	NA	NA	NA	NA
chr3:50012825:50085678	NA	NA	NA	NA	NA	NA
chr3:50211783:50214201	NA	NA	NA	NA	NA	NA
chr3:50312484:50312991	NA	NA	NA	NA	NA	NA
chr3:50338231:50339488	NA	NA	NA	NA	NA	NA
chr3:50388991:50390672	NA	NA	NA	NA	NA	NA
chr3:52110001:52156395	NA	NA	NA	NA	NA	NA
chr3:52436857:52437817	NA	NA	NA	NA	NA	NA
chr3:58116635:58118535	NA	NA	NA	NA	NA	NA
chr3:78695340:78700882	NA	NA	NA	NA	NA	NA
chr3:9471735:9475529	NA	NA	NA	NA	NA	NA
chr4:122735202:122737524	NA	NA	NA	NA	NA	NA
chr4:152065202:152069074	NA	NA	NA	NA	NA	NA
chr4:39306548:39313065	NA	NA	NA	NA	NA	NA
chr4:41621457:41646517	NA	NA	NA	NA	NA	NA
chr4:48385801:48422142	NA	NA	NA	NA	NA	NA
chr4:48850463:48859230	NA	NA	NA	NA	NA	NA
chr4:54257306:54265897	NA	NA	NA	NA	NA	NA
chr4:54266006:54292039	NA	NA	NA	NA	NA	NA
chr4:54294350:54308820	NA	NA	NA	NA	NA	NA
chr4:83346036:83347190	NA	NA	NA	NA	NA	NA
chr4:84378111:84380893	NA	NA	NA	NA	NA	NA
chr5:10227759:10236590	NA	NA	NA	NA	NA	NA
chr5:102296933:102325976	NA	NA	NA	NA	NA	NA
chr5:137492956:137495758	NA	NA	NA	NA	NA	NA
chr5:139844361:139862165	NA	NA	NA	NA	NA	NA
chr5:139866645:139884406	NA	NA	NA	NA	NA	NA
chr5:149769586:149771520	NA	NA	NA	NA	NA	NA
chr5:150489407:150496688	NA	NA	NA	NA	NA	NA
chr5:150506007:150518295	NA	NA	NA	NA	NA	NA
chr5:150512748:150516836	NA	NA	NA	NA	NA	NA
chr5:173036437:173040134	NA	NA	NA	NA	NA	NA
chr5:179291128:179318412	NA	NA	NA	NA	NA	NA
chr5:31515326:31521230	NA	NA	NA	NA	NA	NA
chr5:36606837:36608431	NA	NA	NA	NA	NA	NA
chr5:40767792:40771821	NA	NA	NA	NA	NA	NA
chr5:43299077:43313458	NA	NA	NA	NA	NA	NA
chr5:54619987:54640414	NA	NA	NA	NA	NA	NA
chr5:56510020:56526673	NA	NA	NA	NA	NA	NA
chr5:56531859:56542127	NA	NA	NA	NA	NA	NA
chr5:58284419:58334686	NA	NA	NA	NA	NA	NA
chr5:64948372:64954195	NA	NA	NA	NA	NA	NA
chr5:7871036:7875371	NA	NA	NA	NA	NA	NA
chr5:95765021:95769770	NA	NA	NA	NA	NA	NA
chr6:125569529:125583980	NA	NA	NA	NA	NA	NA
chr6:125574901:125583980	NA	NA	NA	NA	NA	NA
chr6:125614055:125621684	NA	NA	NA	NA	NA	NA
chr6:131191266:131206236	NA	NA	NA	NA	NA	NA
chr6:168312169:168314848	NA	NA	NA	NA	NA	NA
chr6:24409721:24416639	NA	NA	NA	NA	NA	NA
chr6:3012908:3017118	NA	NA	NA	NA	NA	NA
chr6:30865923:30867825	NA	NA	NA	NA	NA	NA
chr6:31856524:31857005	NA	NA	NA	NA	NA	NA
chr6:32942542:32943161	NA	NA	NA	NA	NA	NA
chr6:41035176:41037815	NA	NA	NA	NA	NA	NA

chr6:42902336:42903312	NA	NA	NA	NA	NA	NA
chr6:43746655:43749693	NA	NA	NA	NA	NA	NA
chr6:43746655:43752278	NA	NA	NA	NA	NA	NA
chr6:74227541:74228704	NA	NA	NA	NA	NA	NA
chr6:76344527:76357447	NA	NA	NA	NA	NA	NA
chr7:141438303:141438924	NA	NA	NA	NA	NA	NA
chr7:142982538:142983517	NA	NA	NA	NA	NA	NA
chr7:150773919:150774397	NA	NA	NA	NA	NA	NA
chr7:158542414:158552177	NA	NA	NA	NA	NA	NA
chr7:26230080:26232115	NA	NA	NA	NA	NA	NA
chr7:30538554:30544185	NA	NA	NA	NA	NA	NA
chr7:35922157:35937913	NA	NA	NA	NA	NA	NA
chr7:36450264:36464154	NA	NA	NA	NA	NA	NA
chr7:45143042:45145040	NA	NA	NA	NA	NA	NA
chr7:48134424:48146470	NA	NA	NA	NA	NA	NA
chr7:65439691:65441002	NA	NA	NA	NA	NA	NA
chr7:889240:891021	NA	NA	NA	NA	NA	NA
chr7:99950893:99952766	NA	NA	NA	NA	NA	NA
chr8:124195576:124206264	NA	NA	NA	NA	NA	NA
chr8:128903244:129001408	NA	NA	NA	NA	NA	NA
chr8:130883742:130915558	NA	NA	NA	NA	NA	NA
chr8:134258906:134296507	NA	NA	NA	NA	NA	NA
chr8:144889183:144890779	NA	NA	NA	NA	NA	NA
chr8:17830196:17842956	NA	NA	NA	NA	NA	NA
chr8:23112945:23115589	NA	NA	NA	NA	NA	NA
chr8:42196203:42202471	NA	NA	NA	NA	NA	NA
chr8:87492561:87498713	NA	NA	NA	NA	NA	NA
chr9:101907170:101909936	NA	NA	NA	NA	NA	NA
chr9:111849622:111855755	NA	NA	NA	NA	NA	NA
chr9:112182880:112189230	NA	NA	NA	NA	NA	NA
chr9:128246862:128305338	NA	NA	NA	NA	NA	NA
chr9:132869818:132887194	NA	NA	NA	NA	NA	NA
chr9:134367647:134371127	NA	NA	NA	NA	NA	NA
chr9:139935189:139935514	NA	NA	NA	NA	NA	NA
chr9:5801328:5810011	NA	NA	NA	NA	NA	NA
chr9:6460791:6475391	NA	NA	NA	NA	NA	NA
chr9:80880459:80881358	NA	NA	NA	NA	NA	NA
chr9:86588314:86589432	NA	NA	NA	NA	NA	NA
chr9:99404168:99413672	NA	NA	NA	NA	NA	NA
chrX:102933528:102940099	NA	NA	NA	NA	NA	NA
chrX:106332069:106358582	NA	NA	NA	NA	NA	NA
chrX:123224614:123227868	NA	NA	NA	NA	NA	NA
chrX:128710529:128720979	NA	NA	NA	NA	NA	NA
chrX:134023222:134030847	NA	NA	NA	NA	NA	NA
chrX:13707407:13726840	NA	NA	NA	NA	NA	NA
chrX:13780563:13785246	NA	NA	NA	NA	NA	NA
chrX:147024846:147026464	NA	NA	NA	NA	NA	NA
chrX:153129004:153129777	NA	NA	NA	NA	NA	NA
chrX:23726061:23739998	NA	NA	NA	NA	NA	NA
chrX:23751334:23754036	NA	NA	NA	NA	NA	NA
chrX:31191721:31196786	NA	NA	NA	NA	NA	NA
chrX:39930412:39931602	NA	NA	NA	NA	NA	NA
chrX:48934409:48935496	NA	NA	NA	NA	NA	NA
chrX:9608400:9622255	NA	NA	NA	NA	NA	NA

misoPNN	miso_dACIN	miso_dRNPS1	miso_dPNN	ds_chr	ds_start	ds_stop	dsGFP
NA	**0,14**	**0,18**	**NA**	chr11	130005610	130010293	213,312
NA	**NA**	**-0,145**	**NA**	NA	NA	NA	NA
NA	**NA**	**-0,11**	**NA**	NA	NA	NA	NA
NA	**NA**	**0,235**	**NA**	NA	NA	NA	NA
NA	**0,155**	**NA**	**NA**	chr12	56554104	56555171	676,583
NA	**NA**	**0,105**	**NA**	NA	NA	NA	NA
NA	**-0,145**	**NA**	**NA**	NA	NA	NA	NA
0,195	**NA**	**NA**	**0,265**	NA	NA	NA	NA
0,57	**NA**	**NA**	**-0,165**	chr17	16342728	16343499	1168,09
0,595	**NA**	**-0,105**	**-0,185**	chr17	16342728	16343499	1168,09
NA	**0,26**	**NA**	**NA**	chr17	79977257	79977734	99,3282
0,53	**NA**	**NA**	**-0,265**	chr19	11031803	11032291	64,5904
NA	**NA**	**0,35**	**NA**	chr19	1358463	1360135	36,3472
0,645	**NA**	**NA**	**-0,265**	chr19	50413141	50432583	71,0031
0,63	**NA**	**NA**	**-0,285**	chr19	50413141	50432583	71,0031
0,655	**NA**	**-0,15**	**-0,28**	chr1	207959027	207966864	272,517
NA	**NA**	**-0,315**	**NA**	chr1	234534299	234536927	64,3875
0,34	**NA**	**NA**	**-0,13**	chr1	46072263	46078841	157,671
NA	**-0,345**	**NA**	**NA**	NA	NA	NA	NA
0,65	**NA**	**NA**	**0,28**	chr1	89237562	89251787	85,3754
NA	**-0,3**	**NA**	**NA**	NA	NA	NA	NA
0,755	**NA**	**NA**	**0,155**	NA	NA	NA	NA
NA	**0,16**	**NA**	**NA**	chr21	44317157	44328974	77,1687
NA	**NA**	**-0,275**	**NA**	chr22	29925228	29927820	75,1048
NA	**NA**	**0,29**	**NA**	chr3	172473164	172474773	42,6941
NA	**NA**	**0,125**	**NA**	chr3	183962509	183963256	254,409
NA	**0,125**	**NA**	**NA**	chr3	186502485	186503672	244,28
NA	**-0,195**	**NA**	**NA**	chr3	186505671	186506914	358,036
NA	**-0,195**	**NA**	**NA**	chr3	186505671	186506914	358,036
NA	**NA**	**-0,18**	**NA**	NA	NA	NA	NA
0,325	**NA**	**NA**	**0,305**	NA	NA	NA	NA
0,445	**NA**	**0,385**	**0,44**	NA	NA	NA	NA
0,74	**NA**	**NA**	**0,2**	chr5	304336	311408	538,57
0,785	**NA**	**NA**	**0,17**	chr5	304336	311408	538,57
0,58	**NA**	**NA**	**0,205**	chr5	70900295	70927948	140,122
0,33	**NA**	**0,375**	**0,35**	NA	NA	NA	NA
NA	**NA**	**-0,23**	**NA**	chr6	31611971	31612302	165,184
0,485	**NA**	**NA**	**-0,335**	chr7	134618141	134625843	53,792
0,52	**-0,25**	**-0,315**	**-0,235**	NA	NA	NA	NA
0,515	**NA**	**-0,32**	**-0,245**	NA	NA	NA	NA
NA	**0,12**	**NA**	**NA**	chr7	6431672	6439757	262,673
NA	**0,255**	**NA**	**NA**	chr9	131030803	131036129	55,3462
0,7	**NA**	**NA**	**0,27**	chr9	131353904	131356454	75,1003
0,57	**0,195**	**NA**	**0,16**	NA	NA	NA	NA
0,765	**-0,32**	**-0,24**	**-0,305**	NA	NA	NA	NA
NA	**NA**	**NA**	**NA**	chr10	104828479	104835843	35,9577
NA	**NA**	**NA**	**NA**	chr10	104861083	104865463	53,0689
NA	**NA**	**NA**	**NA**	chr10	120920481	120921852	54,5347
NA	**NA**	**NA**	**NA**	chr10	126449072	126453961	34,4141
NA	**NA**	**NA**	**NA**	chr10	129911866	129914755	40,6616
NA	**NA**	**NA**	**NA**	chr10	27040712	27047991	23,9796
NA	**NA**	**NA**	**NA**	chr10	27815818	27822857	83,7264
NA	**NA**	**NA**	**NA**	chr10	32120728	32128565	17,538
NA	**NA**	**NA**	**NA**	chr10	34690845	34759013	42,0716
NA	**NA**	**NA**	**NA**	chr10	35468204	35495823	35,7698
NA	**NA**	**NA**	**NA**	chr10	70097090	70098260	137,951
NA	**NA**	**NA**	**NA**	chr10	75198178	75204483	26,6713
NA	**NA**	**NA**	**NA**	chr10	75577312	75581440	77,871
NA	**NA**	**NA**	**NA**	chr10	88930731	88939832	32,4838
NA	**NA**	**NA**	**NA**	chr10	95099921	95103613	86,9447
NA	**NA**	**NA**	**NA**	chr10	95148911	95155899	71,2479
NA	**NA**	**NA**	**NA**	chr10	97444381	97446252	68,1435
NA	**NA**	**NA**	**NA**	chr11	102033302	102076624	77,8692

NA	**NA**	**NA**	**NA**	chr11	102076805	102094353	71,7516
NA	**NA**	**NA**	**NA**	chr11	10827557	10828802	313,434
NA	**NA**	**NA**	**NA**	chr11	118128028	118133217	43,771
NA	**NA**	**NA**	**NA**	chr11	12261132	12278332	37,3575
NA	**NA**	**NA**	**NA**	chr11	122928536	122929475	2437,89
NA	**NA**	**NA**	**NA**	chr11	33369559	33370068	15,0305
NA	**NA**	**NA**	**NA**	chr11	36296322	36298639	110,207
NA	**NA**	**NA**	**NA**	chr11	47587538	47593023	61,5333
NA	**NA**	**NA**	**NA**	chr11	502181	506609	20,0978
NA	**NA**	**NA**	**NA**	chr11	62445057	62445399	112,285
NA	**NA**	**NA**	**NA**	chr11	64532990	64534664	103,752
NA	**NA**	**NA**	**NA**	chr11	66612708	66613331	36,6633
NA	**NA**	**NA**	**NA**	chr11	71721900	71723941	37,6948
NA	**NA**	**NA**	**NA**	chr11	72983511	73007530	81,8629
NA	**NA**	**NA**	**NA**	chr11	82985783	82991184	30,247
NA	**NA**	**NA**	**NA**	chr11	85685855	85692172	128,41
NA	**NA**	**NA**	**NA**	chr11	85687725	85692172	95,8786
NA	**NA**	**NA**	**NA**	chr11	8933218	8936373	30,0532
NA	**NA**	**NA**	**NA**	chr11	95598840	95621320	40,9445
NA	**NA**	**NA**	**NA**	chr12	104680887	104705068	94,0591
NA	**NA**	**NA**	**NA**	chr12	10856747	10865810	488,766
NA	**NA**	**NA**	**NA**	chr12	109526317	109530312	70,0486
NA	**NA**	**NA**	**NA**	chr12	111160500	111168456	186,409
NA	**NA**	**NA**	**NA**	chr12	111890644	111893833	31,8605
NA	**NA**	**NA**	**NA**	chr12	112304035	112306557	62,4899
NA	**NA**	**NA**	**NA**	chr12	113716640	113722386	31,145
NA	**NA**	**NA**	**NA**	chr12	121461940	121469245	42,1311
NA	**NA**	**NA**	**NA**	chr12	121683043	121687590	39,1195
NA	**NA**	**NA**	**NA**	chr12	1219513	1225032	22,779
NA	**NA**	**NA**	**NA**	chr12	124824989	124826369	42,9221
NA	**NA**	**NA**	**NA**	chr12	2973918	2975559	102,625
NA	**NA**	**NA**	**NA**	chr12	31236810	31255385	74,9555
NA	**NA**	**NA**	**NA**	chr12	32890095	32892998	75,4067
NA	**NA**	**NA**	**NA**	chr12	394828	401925	24,632
NA	**NA**	**NA**	**NA**	chr12	42768876	42781258	94,8816
NA	**NA**	**NA**	**NA**	chr12	49065745	49073438	44,9948
NA	**NA**	**NA**	**NA**	chr12	50480661	50482304	57,1681
NA	**NA**	**NA**	**NA**	chr12	51442968	51450133	63,7319
NA	**NA**	**NA**	**NA**	chr12	53898599	53899433	59,6072
NA	**NA**	**NA**	**NA**	chr12	54741823	54744260	419,281
NA	**NA**	**NA**	**NA**	chr12	56362732	56364828	113,193
NA	**NA**	**NA**	**NA**	chr12	57065593	57066736	484,354
NA	**NA**	**NA**	**NA**	chr12	57501097	57501946	52,6871
NA	**NA**	**NA**	**NA**	chr12	58112965	58114189	108,019
NA	**NA**	**NA**	**NA**	chr12	6495610	6497966	221,4
NA	**NA**	**NA**	**NA**	chr12	6690300	6700704	166,287
NA	**NA**	**NA**	**NA**	chr12	6780004	6782382	65,9586
NA	**NA**	**NA**	**NA**	chr12	6787626	6788112	49,898
NA	**NA**	**NA**	**NA**	chr12	6833984	6837389	123,5
NA	**NA**	**NA**	**NA**	chr12	7084310	7084858	254,018
NA	**NA**	**NA**	**NA**	chr12	7354437	7355208	48,1547
NA	**NA**	**NA**	**NA**	chr12	95645847	95650926	27,3276
NA	**NA**	**NA**	**NA**	chr12	987527	990858	53,9223
NA	**NA**	**NA**	**NA**	chr13	114193822	114202618	37,8278
NA	**NA**	**NA**	**NA**	chr13	114291015	114294435	170,68
NA	**NA**	**NA**	**NA**	chr13	115057267	115067206	61,7902
NA	**NA**	**NA**	**NA**	chr13	37563692	37569126	84,204
NA	**NA**	**NA**	**NA**	chr14	102695994	102698872	21,2827
NA	**NA**	**NA**	**NA**	chr14	105181193	105185132	49,8963
NA	**NA**	**NA**	**NA**	chr14	105911848	105916395	21,7566
NA	**NA**	**NA**	**NA**	chr14	21460364	21465044	67,5971
NA	**NA**	**NA**	**NA**	chr14	23396823	23397706	113,822
NA	**NA**	**NA**	**NA**	chr14	56128330	56133959	131,535
NA	**NA**	**NA**	**NA**	chr14	65560533	65569022	32,7717
NA	**NA**	**NA**	**NA**	chr14	89063152	89073587	41,0756
NA	**NA**	**NA**	**NA**	chr14	93170592	93182530	58,9245

NA	**NA**	**NA**	**NA**	chr14	96991728	96994290	81,2184
NA	**NA**	**NA**	**NA**	chr15	101866684	101872057	13,1998
NA	**NA**	**NA**	**NA**	chr15	41102955	41105536	31,0992
NA	**NA**	**NA**	**NA**	chr15	66786890	66789980	41,7685
NA	**NA**	**NA**	**NA**	chr15	69629840	69672222	11,5413
NA	**NA**	**NA**	**NA**	chr15	72542433	72543548	28,9844
NA	**NA**	**NA**	**NA**	chr15	76206546	76225026	69,779
NA	**NA**	**NA**	**NA**	chr15	91510432	91512677	51,2436
NA	**NA**	**NA**	**NA**	chr16	15180115	15185172	23,3787
NA	**NA**	**NA**	**NA**	chr16	2809662	2810302	194,328
NA	**NA**	**NA**	**NA**	chr16	29842394	29851724	77,6168
NA	**NA**	**NA**	**NA**	chr16	29982899	29984271	55,3536
NA	**NA**	**NA**	**NA**	chr16	46836982	46858298	26,3045
NA	**NA**	**NA**	**NA**	chr16	47117712	47143394	44,6984
NA	**NA**	**NA**	**NA**	chr16	4873908	4882775	89,5087
NA	**NA**	**NA**	**NA**	chr16	53501018	53515626	20,2667
NA	**NA**	**NA**	**NA**	chr16	54954239	54957497	101,194
NA	**NA**	**NA**	**NA**	chr16	57792821	57793640	98,2481
NA	**NA**	**NA**	**NA**	chr16	68380183	68386151	51,8352
NA	**NA**	**NA**	**NA**	chr16	69154552	69155339	53,1111
NA	**NA**	**NA**	**NA**	chr16	70582345	70588349	203,55
NA	**NA**	**NA**	**NA**	chr16	71799487	71803526	31,526
NA	**NA**	**NA**	**NA**	chr16	720557	720892	42,0736
NA	**NA**	**NA**	**NA**	chr16	87760530	87782279	101,596
NA	**NA**	**NA**	**NA**	chr16	87788898	87795555	72,3142
NA	**NA**	**NA**	**NA**	chr16	88653092	88664586	40,3679
NA	**NA**	**NA**	**NA**	chr16	88783619	88785982	144,553
NA	**NA**	**NA**	**NA**	chr16	89818630	89828357	22,2765
NA	**NA**	**NA**	**NA**	chr17	15989756	15995177	38,1259
NA	**NA**	**NA**	**NA**	chr17	16052831	16055260	41,0608
NA	**NA**	**NA**	**NA**	chr17	17046079	17049350	68,3229
NA	**NA**	**NA**	**NA**	chr17	18148559	18152441	108,474
NA	**NA**	**NA**	**NA**	chr17	18152035	18152441	95,6668
NA	**NA**	**NA**	**NA**	chr17	20906155	20924519	104,584
NA	**NA**	**NA**	**NA**	chr17	27409456	27413456	46,5052
NA	**NA**	**NA**	**NA**	chr17	2861088	2866724	13,9993
NA	**NA**	**NA**	**NA**	chr17	29576137	29585362	16,7057
NA	**NA**	**NA**	**NA**	chr17	30678902	30687746	139,246
NA	**NA**	**NA**	**NA**	chr17	30692506	30694791	121,926
NA	**NA**	**NA**	**NA**	chr17	35564714	35578645	46,636
NA	**NA**	**NA**	**NA**	chr17	41103911	41106893	76,5044
NA	**NA**	**NA**	**NA**	chr17	41107989	41108421	76,8311
NA	**NA**	**NA**	**NA**	chr17	46133960	46134706	95,138
NA	**NA**	**NA**	**NA**	chr17	47482404	47486497	654,815
NA	**NA**	**NA**	**NA**	chr17	48156290	48167554	89,4431
NA	**NA**	**NA**	**NA**	chr17	5326149	5329556	36,0736
NA	**NA**	**NA**	**NA**	chr17	5326149	5335862	197,262
NA	**NA**	**NA**	**NA**	chr17	5329619	5335862	31,2845
NA	**NA**	**NA**	**NA**	chr17	62496891	62498128	528,862
NA	**NA**	**NA**	**NA**	chr17	65871136	65887960	18,2687
NA	**NA**	**NA**	**NA**	chr17	66247319	66252955	76,5946
NA	**NA**	**NA**	**NA**	chr17	71205907	71223304	34,9795
NA	**NA**	**NA**	**NA**	chr17	7123845	7124264	141,533
NA	**NA**	**NA**	**NA**	chr17	74085401	74090495	60,4599
NA	**NA**	**NA**	**NA**	chr17	7490064	7491413	124,779
NA	**NA**	**NA**	**NA**	chr17	74922812	74934070	35,8821
NA	**NA**	**NA**	**NA**	chr17	75398577	75483564	201,438
NA	**NA**	**NA**	**NA**	chr17	7810806	7811212	147,686
NA	**NA**	**NA**	**NA**	chr17	79478604	79478953	2208,12
NA	**NA**	**NA**	**NA**	chr17	79891252	79892802	291,552
NA	**NA**	**NA**	**NA**	chr17	8473130	8480554	43,3371
NA	**NA**	**NA**	**NA**	chr18	675402	683246	77,1415
NA	**NA**	**NA**	**NA**	chr19	10381902	10394805	93,063
NA	**NA**	**NA**	**NA**	chr19	1251900	1254166	39,352
NA	**NA**	**NA**	**NA**	chr19	13220652	13220972	61,6484
NA	**NA**	**NA**	**NA**	chr19	14501891	14508470	74,8159

NA	**NA**	**NA**	**NA**	chr19	1627425	1646354	40,5279
NA	**NA**	**NA**	**NA**	chr19	17338008	17339065	36,3351
NA	**NA**	**NA**	**NA**	chr19	18541740	18542457	68,2191
NA	**NA**	**NA**	**NA**	chr19	19307855	19308330	80,7648
NA	**NA**	**NA**	**NA**	chr19	30101364	30103024	45,2161
NA	**NA**	**NA**	**NA**	chr19	34699956	34706501	122,855
NA	**NA**	**NA**	**NA**	chr19	3546166	3547270	107,002
NA	**NA**	**NA**	**NA**	chr19	36122307	36124000	39,1916
NA	**NA**	**NA**	**NA**	chr19	41120352	41125252	25,2798
NA	**NA**	**NA**	**NA**	chr19	41125398	41129509	39,337
NA	**NA**	**NA**	**NA**	chr19	41938313	41939442	35,5414
NA	**NA**	**NA**	**NA**	chr19	41939578	41944123	39,0987
NA	**NA**	**NA**	**NA**	chr19	45161178	45164559	54,5613
NA	**NA**	**NA**	**NA**	chr19	47111584	47112181	219,185
NA	**NA**	**NA**	**NA**	chr19	50168103	50168929	29,8293
NA	**NA**	**NA**	**NA**	chr19	51848500	51856407	206,13
NA	**NA**	**NA**	**NA**	chr19	5218543	5219321	43,1977
NA	**NA**	**NA**	**NA**	chr19	55966697	55967716	133,921
NA	**NA**	**NA**	**NA**	chr19	6222695	6230581	77,1682
NA	**NA**	**NA**	**NA**	chr19	805187	806408	267,314
NA	**NA**	**NA**	**NA**	chr1	117146647	117150564	14,9518
NA	**NA**	**NA**	**NA**	chr1	12027148	12035340	170,244
NA	**NA**	**NA**	**NA**	chr1	148561115	148568736	134,858
NA	**NA**	**NA**	**NA**	chr1	150201477	150208081	109,091
NA	**NA**	**NA**	**NA**	chr1	150464965	150470006	65,9334
NA	**NA**	**NA**	**NA**	chr1	151733375	151734581	125,708
NA	**NA**	**NA**	**NA**	chr1	154185100	154186933	372,58
NA	**NA**	**NA**	**NA**	chr1	154187050	154192818	279,915
NA	**NA**	**NA**	**NA**	chr1	154241430	154242676	23,0448
NA	**NA**	**NA**	**NA**	chr1	155108852	155110037	67,2206
NA	**NA**	**NA**	**NA**	chr1	155230450	155231877	113,525
NA	**NA**	**NA**	**NA**	chr1	15752514	15755089	90,1843
NA	**NA**	**NA**	**NA**	chr1	160186698	160192446	94,9303
NA	**NA**	**NA**	**NA**	chr1	162560301	162567582	107,517
NA	**NA**	**NA**	**NA**	chr1	164781386	164790774	33,7255
NA	**NA**	**NA**	**NA**	chr1	167742605	167757057	154,826
NA	**NA**	**NA**	**NA**	chr1	176104222	176118142	35,1901
NA	**NA**	**NA**	**NA**	chr1	181019422	181022709	11,3411
NA	**NA**	**NA**	**NA**	chr1	184023997	184041961	20,886
NA	**NA**	**NA**	**NA**	chr1	201453186	201458001	232,058
NA	**NA**	**NA**	**NA**	chr1	211477482	211486062	30,558
NA	**NA**	**NA**	**NA**	chr1	225594534	225599039	78,1706
NA	**NA**	**NA**	**NA**	chr1	225688772	225695653	148,167
NA	**NA**	**NA**	**NA**	chr1	228296019	228296850	49,9345
NA	**NA**	**NA**	**NA**	chr1	2328659	2332296	109,066
NA	**NA**	**NA**	**NA**	chr1	23357127	23376880	57,0127
NA	**NA**	**NA**	**NA**	chr1	241761299	241803184	49,1149
NA	**NA**	**NA**	**NA**	chr1	242013888	242016660	75,9629
NA	**NA**	**NA**	**NA**	chr1	24294213	24298063	61,657
NA	**NA**	**NA**	**NA**	chr1	24989295	24993306	36,1418
NA	**NA**	**NA**	**NA**	chr1	36639079	36641800	62,1212
NA	**NA**	**NA**	**NA**	chr1	37959415	37967405	79,2117
NA	**NA**	**NA**	**NA**	chr1	37961519	37967405	67,569
NA	**NA**	**NA**	**NA**	chr1	38450451	38455220	144,365
NA	**NA**	**NA**	**NA**	chr1	39945681	39950273	45,8362
NA	**NA**	**NA**	**NA**	chr1	41471766	41474331	126,336
NA	**NA**	**NA**	**NA**	chr1	42664901	42693554	32,6387
NA	**NA**	**NA**	**NA**	chr1	47152542	47155259	38,1379
NA	**NA**	**NA**	**NA**	chr1	52215867	52226362	48,1157
NA	**NA**	**NA**	**NA**	chr1	53720857	53723991	56,107
NA	**NA**	**NA**	**NA**	chr1	53737018	53741303	27,841
NA	**NA**	**NA**	**NA**	chr1	54254869	54262362	102,625
NA	**NA**	**NA**	**NA**	chr1	54412222	54417659	44,4542
NA	**NA**	**NA**	**NA**	chr1	61872399	61920975	43,7384
NA	**NA**	**NA**	**NA**	chr1	63009416	63018403	48,6383
NA	**NA**	**NA**	**NA**	chr1	70694238	70696778	131,017

NA	**NA**	**NA**	**NA**	chr1	75172678	75175782	65,4477
NA	**NA**	**NA**	**NA**	chr1	75190518	75198640	28,4606
NA	**NA**	**NA**	**NA**	chr1	85031695	85036265	25,8556
NA	**NA**	**NA**	**NA**	chr1	85724405	85725041	55,5977
NA	**NA**	**NA**	**NA**	chr1	8617582	8684369	19,7975
NA	**NA**	**NA**	**NA**	chr1	87329288	87346345	388,335
NA	**NA**	**NA**	**NA**	chr1	9796100	9801152	70,8609
NA	**NA**	**NA**	**NA**	chr20	11899249	11900366	54,9255
NA	**NA**	**NA**	**NA**	chr20	1435777	1438845	57,1273
NA	**NA**	**NA**	**NA**	chr20	30149539	30156923	156,391
NA	**NA**	**NA**	**NA**	chr20	32661441	32663680	144,993
NA	**NA**	**NA**	**NA**	chr20	34219947	34220233	47,1273
NA	**NA**	**NA**	**NA**	chr20	34326939	34328746	83,8022
NA	**NA**	**NA**	**NA**	chr20	34785963	34800194	23,2018
NA	**NA**	**NA**	**NA**	chr20	34800298	34806798	32,4425
NA	**NA**	**NA**	**NA**	chr20	37055146	37059684	49,0553
NA	**NA**	**NA**	**NA**	chr20	43249788	43251470	81,0441
NA	**NA**	**NA**	**NA**	chr20	43251571	43257688	50,8978
NA	**NA**	**NA**	**NA**	chr20	45839542	45848909	30,155
NA	**NA**	**NA**	**NA**	chr20	5564968	5573973	9,30208
NA	**NA**	**NA**	**NA**	chr20	61299262	61299829	151,499
NA	**NA**	**NA**	**NA**	chr21	34900641	34903065	167,683
NA	**NA**	**NA**	**NA**	chr22	24379450	24384120	50,7202
NA	**NA**	**NA**	**NA**	chr22	24936941	24943876	57,0719
NA	**NA**	**NA**	**NA**	chr22	29668420	29674120	279,609
NA	**NA**	**NA**	**NA**	chr22	29684744	29688489	237,428
NA	**NA**	**NA**	**NA**	chr22	29724884	29726367	70,8466
NA	**NA**	**NA**	**NA**	chr22	46751485	46752739	47,809
NA	**NA**	**NA**	**NA**	chr22	50895102	50897684	31,2046
NA	**NA**	**NA**	**NA**	chr2	100010861	100015211	142,777
NA	**NA**	**NA**	**NA**	chr2	101565942	101584743	25,8321
NA	**NA**	**NA**	**NA**	chr2	102476326	102481392	43,8104
NA	**NA**	**NA**	**NA**	chr2	102486877	102490109	101,716
NA	**NA**	**NA**	**NA**	chr2	127808488	127816587	40,8738
NA	**NA**	**NA**	**NA**	chr2	1687923	1695700	67,4284
NA	**NA**	**NA**	**NA**	chr2	188368497	188418927	27,3023
NA	**NA**	**NA**	**NA**	chr2	192265194	192267358	14,6775
NA	**NA**	**NA**	**NA**	chr2	201284219	201305372	25,8574
NA	**NA**	**NA**	**NA**	chr2	201724469	201725961	34,6467
NA	**NA**	**NA**	**NA**	chr2	228205096	228211942	39,5144
NA	**NA**	**NA**	**NA**	chr2	230664045	230723501	30,9826
NA	**NA**	**NA**	**NA**	chr2	230724290	230744698	30,6498
NA	**NA**	**NA**	**NA**	chr2	234181698	234183322	14,3991
NA	**NA**	**NA**	**NA**	chr2	236817550	236877106	17,5481
NA	**NA**	**NA**	**NA**	chr2	27549085	27550044	95,9466
NA	**NA**	**NA**	**NA**	chr2	27995559	28002300	117,313
NA	**NA**	**NA**	**NA**	chr2	28521358	28561317	74,3678
NA	**NA**	**NA**	**NA**	chr2	36583766	36668401	56,1261
NA	**NA**	**NA**	**NA**	chr2	36749456	36771519	53,1838
NA	**NA**	**NA**	**NA**	chr2	61408540	61411847	33,3401
NA	**NA**	**NA**	**NA**	chr2	70515324	70520750	212,402
NA	**NA**	**NA**	**NA**	chr2	74759052	74759947	77,601
NA	**NA**	**NA**	**NA**	chr2	9547034	9548242	94,656
NA	**NA**	**NA**	**NA**	chr3	100211772	100274053	99,1771
NA	**NA**	**NA**	**NA**	chr3	107497366	107510087	25,9386
NA	**NA**	**NA**	**NA**	chr3	107766139	107769425	28,2012
NA	**NA**	**NA**	**NA**	chr3	111639266	111658322	53,3786
NA	**NA**	**NA**	**NA**	chr3	113440663	113442828	105,406
NA	**NA**	**NA**	**NA**	chr3	124732834	124738106	48,1617
NA	**NA**	**NA**	**NA**	chr3	127294348	127294783	65,3733
NA	**NA**	**NA**	**NA**	chr3	128890381	128890498	140,378
NA	**NA**	**NA**	**NA**	chr3	130678185	130682815	68,1126
NA	**NA**	**NA**	**NA**	chr3	131208924	131219274	152,506
NA	**NA**	**NA**	**NA**	chr3	133305566	133306740	107,141
NA	**NA**	**NA**	**NA**	chr3	133305566	133307197	137,395
NA	**NA**	**NA**	**NA**	chr3	135969310	135980806	85,2922

NA	**NA**	**NA**	**NA**	chr3	145794682	145796903	93,7789
NA	**NA**	**NA**	**NA**	chr3	154858081	154861232	97,0732
NA	**NA**	**NA**	**NA**	chr3	183558428	183562019	82,6648
NA	**NA**	**NA**	**NA**	chr3	184035285	184035538	244,632
NA	**NA**	**NA**	**NA**	chr3	196876667	196921296	36,7725
NA	**NA**	**NA**	**NA**	chr3	27446454	27453133	14,604
NA	**NA**	**NA**	**NA**	chr3	33444396	33450728	92,9875
NA	**NA**	**NA**	**NA**	chr3	37125297	37136283	31,1679
NA	**NA**	**NA**	**NA**	chr3	38167372	38168001	43,5706
NA	**NA**	**NA**	**NA**	chr3	38170879	38173417	30,6588
NA	**NA**	**NA**	**NA**	chr3	44970928	44974610	75,0384
NA	**NA**	**NA**	**NA**	chr3	47958664	47963255	174,276
NA	**NA**	**NA**	**NA**	chr3	50012825	50085678	30,3424
NA	**NA**	**NA**	**NA**	chr3	50211783	50214201	66,6475
NA	**NA**	**NA**	**NA**	chr3	50312484	50312991	76,1813
NA	**NA**	**NA**	**NA**	chr3	50338231	50339488	66,9917
NA	**NA**	**NA**	**NA**	chr3	50388991	50390672	49,5531
NA	**NA**	**NA**	**NA**	chr3	52110001	52156395	31,5627
NA	**NA**	**NA**	**NA**	chr3	52436857	52437817	93,95
NA	**NA**	**NA**	**NA**	chr3	58116635	58118535	33,8641
NA	**NA**	**NA**	**NA**	chr3	78695340	78700882	29,7725
NA	**NA**	**NA**	**NA**	chr3	9471735	9475529	26,5167
NA	**NA**	**NA**	**NA**	chr4	122735202	122737524	37,912
NA	**NA**	**NA**	**NA**	chr4	152065202	152069074	25,3864
NA	**NA**	**NA**	**NA**	chr4	39306548	39313065	45,3827
NA	**NA**	**NA**	**NA**	chr4	41621457	41646517	40,0472
NA	**NA**	**NA**	**NA**	chr4	48385801	48422142	26,0029
NA	**NA**	**NA**	**NA**	chr4	48850463	48859230	82,1073
NA	**NA**	**NA**	**NA**	chr4	54257306	54265897	41,2958
NA	**NA**	**NA**	**NA**	chr4	54266006	54292039	54,4004
NA	**NA**	**NA**	**NA**	chr4	54294350	54308820	40,6934
NA	**NA**	**NA**	**NA**	chr4	83346036	83347190	322,567
NA	**NA**	**NA**	**NA**	chr4	84378111	84380893	76,4567
NA	**NA**	**NA**	**NA**	chr5	10227759	10236590	23,6201
NA	**NA**	**NA**	**NA**	chr5	102296933	102325976	78,2647
NA	**NA**	**NA**	**NA**	chr5	137492956	137495758	57,0202
NA	**NA**	**NA**	**NA**	chr5	139844361	139862165	37,5386
NA	**NA**	**NA**	**NA**	chr5	139866645	139884406	53,0374
NA	**NA**	**NA**	**NA**	chr5	149769586	149771520	100,51
NA	**NA**	**NA**	**NA**	chr5	150489407	150496688	147,263
NA	**NA**	**NA**	**NA**	chr5	150506007	150518295	119,502
NA	**NA**	**NA**	**NA**	chr5	150512748	150516836	122,931
NA	**NA**	**NA**	**NA**	chr5	173036437	173040134	78,1306
NA	**NA**	**NA**	**NA**	chr5	179291128	179318412	58,5781
NA	**NA**	**NA**	**NA**	chr5	31515326	31521230	50,5597
NA	**NA**	**NA**	**NA**	chr5	36606837	36608431	99,314
NA	**NA**	**NA**	**NA**	chr5	40767792	40771821	49,9807
NA	**NA**	**NA**	**NA**	chr5	43299077	43313458	37,4249
NA	**NA**	**NA**	**NA**	chr5	54619987	54640414	66,3772
NA	**NA**	**NA**	**NA**	chr5	56510020	56526673	34,8389
NA	**NA**	**NA**	**NA**	chr5	56531859	56542127	36,1763
NA	**NA**	**NA**	**NA**	chr5	58284419	58334686	150,807
NA	**NA**	**NA**	**NA**	chr5	64948372	64954195	22,1994
NA	**NA**	**NA**	**NA**	chr5	7871036	7875371	43,5888
NA	**NA**	**NA**	**NA**	chr5	95765021	95769770	56,4229
NA	**NA**	**NA**	**NA**	chr6	125569529	125583980	93,2454
NA	**NA**	**NA**	**NA**	chr6	125574901	125583980	47,4191
NA	**NA**	**NA**	**NA**	chr6	125614055	125621684	63,2948
NA	**NA**	**NA**	**NA**	chr6	131191266	131206236	39,2477
NA	**NA**	**NA**	**NA**	chr6	168312169	168314848	37,5216
NA	**NA**	**NA**	**NA**	chr6	24409721	24416639	37,7825
NA	**NA**	**NA**	**NA**	chr6	3012908	3017118	257,075
NA	**NA**	**NA**	**NA**	chr6	30865923	30867825	63,5414
NA	**NA**	**NA**	**NA**	chr6	31856524	31857005	47,6665
NA	**NA**	**NA**	**NA**	chr6	32942542	32943161	126,432
NA	**NA**	**NA**	**NA**	chr6	41035176	41037815	30,469

NA	**NA**	**NA**	**NA**	chr6	42902336	42903312	51,2942
NA	**NA**	**NA**	**NA**	chr6	43746655	43749693	32,3812
NA	**NA**	**NA**	**NA**	chr6	43746655	43752278	64,5495
NA	**NA**	**NA**	**NA**	chr6	74227541	74228704	8891,39
NA	**NA**	**NA**	**NA**	chr6	76344527	76357447	39,5292
NA	**NA**	**NA**	**NA**	chr7	141438303	141438924	68,3527
NA	**NA**	**NA**	**NA**	chr7	142982538	142983517	21,2298
NA	**NA**	**NA**	**NA**	chr7	150773919	150774397	68,2891
NA	**NA**	**NA**	**NA**	chr7	158542414	158552177	99,7672
NA	**NA**	**NA**	**NA**	chr7	26230080	26232115	123,942
NA	**NA**	**NA**	**NA**	chr7	30538554	30544185	106,378
NA	**NA**	**NA**	**NA**	chr7	35922157	35937913	95,6291
NA	**NA**	**NA**	**NA**	chr7	36450264	36464154	87,1117
NA	**NA**	**NA**	**NA**	chr7	45143042	45145040	169,481
NA	**NA**	**NA**	**NA**	chr7	48134424	48146470	30,7469
NA	**NA**	**NA**	**NA**	chr7	65439691	65441002	36,7532
NA	**NA**	**NA**	**NA**	chr7	889240	891021	40,4583
NA	**NA**	**NA**	**NA**	chr7	99950893	99952766	49,0736
NA	**NA**	**NA**	**NA**	chr8	124195576	124206264	33,7696
NA	**NA**	**NA**	**NA**	chr8	128903244	129001408	30,3707
NA	**NA**	**NA**	**NA**	chr8	130883742	130915558	39,4276
NA	**NA**	**NA**	**NA**	chr8	134258906	134296507	32,5381
NA	**NA**	**NA**	**NA**	chr8	144889183	144890779	64,2054
NA	**NA**	**NA**	**NA**	chr8	17830196	17842956	19,4403
NA	**NA**	**NA**	**NA**	chr8	23112945	23115589	37,0411
NA	**NA**	**NA**	**NA**	chr8	42196203	42202471	25,6627
NA	**NA**	**NA**	**NA**	chr8	87492561	87498713	36,1958
NA	**NA**	**NA**	**NA**	chr9	101907170	101909936	196,726
NA	**NA**	**NA**	**NA**	chr9	111849622	111855755	47,0678
NA	**NA**	**NA**	**NA**	chr9	112182880	112189230	21,9941
NA	**NA**	**NA**	**NA**	chr9	128246862	128305338	78,2459
NA	**NA**	**NA**	**NA**	chr9	132869818	132887194	36,9799
NA	**NA**	**NA**	**NA**	chr9	134367647	134371127	49,7603
NA	**NA**	**NA**	**NA**	chr9	139935189	139935514	34,9012
NA	**NA**	**NA**	**NA**	chr9	5801328	5810011	66,3169
NA	**NA**	**NA**	**NA**	chr9	6460791	6475391	23,2682
NA	**NA**	**NA**	**NA**	chr9	80880459	80881358	27,0779
NA	**NA**	**NA**	**NA**	chr9	86588314	86589432	735,509
NA	**NA**	**NA**	**NA**	chr9	99404168	99413672	30,2332
NA	**NA**	**NA**	**NA**	chrX	102933528	102940099	475,198
NA	**NA**	**NA**	**NA**	chrX	106332069	106358582	16,0223
NA	**NA**	**NA**	**NA**	chrX	123224614	123227868	83,869
NA	**NA**	**NA**	**NA**	chrX	128710529	128720979	28,2369
NA	**NA**	**NA**	**NA**	chrX	134023222	134030847	29,3408
NA	**NA**	**NA**	**NA**	chrX	13707407	13726840	62,7126
NA	**NA**	**NA**	**NA**	chrX	13780563	13785246	19,8912
NA	**NA**	**NA**	**NA**	chrX	147024846	147026464	27,4735
NA	**NA**	**NA**	**NA**	chrX	153129004	153129777	708,903
NA	**NA**	**NA**	**NA**	chrX	23726061	23739998	72,6532
NA	**NA**	**NA**	**NA**	chrX	23751334	23754036	63,689
NA	**NA**	**NA**	**NA**	chrX	31191721	31196786	83,2472
NA	**NA**	**NA**	**NA**	chrX	39930412	39931602	25,0872
NA	**NA**	**NA**	**NA**	chrX	48934409	48935496	30,2608
NA	**NA**	**NA**	**NA**	chrX	9608400	9622255	39,0331

dsACIN	dsRNPS1	dsPNN	**ds_dACIN**	**ds_dRNPS1**	**ds_dPNN**	sJSD_ACIN	sJSD_RNPS1
206,451	299,216	NA	**0,151454**	**0,2001095**	**NA**	0,143403	0,195962
NA	NA	NA	**NA**	**NA**	**NA**	NA	NA
NA	NA	NA	**NA**	**NA**	**NA**	NA	NA
NA	NA	NA	**NA**	**NA**	**NA**	NA	NA
1097,25	NA	NA	**0,1834185**	**NA**	**NA**	0,157958	NA
NA	NA	NA	**NA**	**NA**	**NA**	NA	NA
NA	NA	NA	**NA**	**NA**	**NA**	NA	NA
NA	NA	NA	**NA**	**NA**	**NA**	NA	NA
NA	590,525	474,28	**NA**	**-0,1636445**	**-0,2151725**	NA	0,144763
NA	590,525	474,28	**NA**	**-0,1636445**	**-0,2151725**	NA	0,144763
83,6477	100,748	61,0322	**0,2426515**	**0,2157585**	**0,168136**	0,207524	0,184081
NA	87,6201	67,9488	**NA**	**-0,12893**	**-0,258648**	NA	0,121094
NA	34,023	31,9345	**NA**	**0,3221495**	**0,2181545**	NA	0,283846
NA	NA	64,5851	**NA**	**NA**	**-0,2434035**	NA	NA
NA	NA	64,5851	**NA**	**NA**	**-0,2434035**	NA	NA
NA	NA	622,489	**NA**	**NA**	**-0,250241**	NA	NA
NA	58,1087	36,1557	**NA**	**-0,3661005**	**-0,410366**	NA	0,328005
NA	NA	209,213	**NA**	**NA**	**-0,1614735**	NA	NA
NA	NA	NA	**NA**	**NA**	**NA**	NA	NA
NA	64,2446	81,1496	**NA**	**0,270765**	**0,379611**	NA	0,316026
NA	NA	NA	**NA**	**NA**	**NA**	NA	NA
NA	NA	NA	**NA**	**NA**	**NA**	NA	NA
102,697	NA	NA	**0,1152765**	**NA**	**NA**	0,110812	NA
NA	60,2032	73,213	**NA**	**-0,2575555**	**-0,1787215**	NA	0,246739
59,9693	88,958	81,5514	**0,297429**	**0,380551**	**0,2822115**	0,306821	0,374291
NA	127,12	NA	**NA**	**0,118788**	**NA**	NA	0,249201
238,897	NA	NA	**0,105149**	**NA**	**NA**	0,103502	NA
313,902	NA	NA	**-0,192333**	**NA**	**NA**	0,179934	NA
313,902	NA	NA	**-0,192333**	**NA**	**NA**	0,179934	NA
NA	NA	NA	**NA**	**NA**	**NA**	NA	NA
NA	NA	NA	**NA**	**NA**	**NA**	NA	NA
NA	NA	NA	**NA**	**NA**	**NA**	NA	NA
NA	NA	516,084	**NA**	**NA**	**0,01022**	NA	NA
NA	NA	516,084	**NA**	**NA**	**0,01022**	NA	NA
NA	134,94	128,541	**NA**	**0,0970245**	**0,1632485**	NA	0,103569
NA	NA	NA	**NA**	**NA**	**NA**	NA	NA
NA	113,476	NA	**NA**	**-0,228758**	**NA**	NA	0,199079
NA	NA	62,732	**NA**	**NA**	**-0,385137**	NA	NA
NA	NA	NA	**NA**	**NA**	**NA**	NA	NA
NA	NA	NA	**NA**	**NA**	**NA**	NA	NA
252,504	352,608	301,497	**0,1569467**	**0,1088595**	**0,1084435**	0,192912	0,123862
57,9533	58,3413	47,864	**0,300461**	**0,149935**	**0,128752**	0,283739	0,131949
92,5947	NA	84,5184	**0,120356**	**NA**	**0,337989**	0,250917	NA
NA	NA	NA	**NA**	**NA**	**NA**	NA	NA
NA	NA	NA	**NA**	**NA**	**NA**	NA	NA
24,5915	NA	NA	**0,129729**	**NA**	**NA**	0,121536	NA
NA	79,7176	62,7243	**NA**	**-0,09139845**	**-0,10972895**	NA	0,115964
49,2406	55,9493	67,7638	**0,142043**	**0,2585705**	**0,276915**	0,149365	0,251965
NA	NA	43,793	**NA**	**NA**	**-0,146012**	NA	NA
NA	NA	23,2111	**NA**	**NA**	**0,130986**	NA	NA
24,6466	34,2449	32,1586	**0,2246625**	**0,3196615**	**0,277768**	0,193293	0,274061
NA	NA	138,447	**NA**	**NA**	**0,2192195**	NA	NA
27,4135	32,3109	24,7849	**0,236141**	**0,1577065**	**0,1516265**	0,210774	0,144384
39,3341	40,2324	NA	**-0,189048**	**-0,192908**	**NA**	0,172	0,175832
NA	NA	27,3088	**NA**	**NA**	**-0,1728545**	NA	NA
147,489	NA	NA	**0,101237**	**NA**	**NA**	0,134755	NA
NA	NA	47,014	**NA**	**NA**	**0,1536335**	NA	NA
53,0986	NA	NA	**-0,0888286**	**NA**	**NA**	0,108965	NA
48,9244	NA	NA	**0,183828**	**NA**	**NA**	0,173882	NA
67,8037	NA	NA	**-0,1089345**	**NA**	**NA**	0,111062	NA
49,362	NA	96,0649	**0,1976845**	**NA**	**0,1183155**	0,188398	NA
NA	NA	63,6899	**NA**	**NA**	**0,119602**	NA	NA
NA	NA	75,37	**NA**	**NA**	**-0,134053**	NA	NA

NA	NA	65,3273	**NA**	**NA**	**-0,169157**	NA	NA
301,494	457,09	NA	**-0,0750915**	**-0,0638963**	**NA**	0,140868	0,125051
NA	91,8192	NA	**NA**	**0,2345585**	**NA**	NA	0,358542
NA	30,5965	NA	**NA**	**0,1332795**	**NA**	NA	0,127592
NA	NA	2304,59	**NA**	**NA**	**-0,06265575**	NA	NA
NA	26,9539	NA	**NA**	**-0,148921**	**NA**	NA	0,128727
NA	92,4962	101,565	**NA**	**-0,0725495**	**-0,07304**	NA	0,104543
49,5944	NA	NA	**0,1124665**	**NA**	**NA**	0,102967	NA
18,9495	NA	NA	**0,101751**	**NA**	**NA**	0,139922	NA
NA	106,97	166,616	**NA**	**-0,11387635**	**-0,11885085**	NA	0,132484
NA	113,705	NA	**NA**	**0,159012**	**NA**	NA	0,29063
NA	NA	37,4087	**NA**	**NA**	**0,122275**	NA	NA
40,1884	38,5659	44,0842	**0,336451**	**0,1453025**	**0,1520475**	0,288984	0,124882
74,1974	NA	NA	**0,1754775**	**NA**	**NA**	0,162634	NA
NA	NA	36,2465	**NA**	**NA**	**0,126731**	NA	NA
NA	NA	145,913	**NA**	**NA**	**0,076315**	NA	NA
108,493	NA	86,336	**0,096933**	**NA**	**0,1663945**	0,116256	NA
31,6082	50,1541	NA	**-0,288698**	**-0,247751**	**NA**	0,25998	0,21948
NA	NA	71,954	**NA**	**NA**	**-0,10834305**	NA	NA
NA	131,918	NA	**NA**	**-0,075894**	**NA**	NA	0,111971
385,237	NA	NA	**-0,106438**	**NA**	**NA**	0,105988	NA
NA	NA	83,5218	**NA**	**NA**	**-0,1752655**	NA	NA
208,344	242,529	242,048	**-0,0843115**	**-0,073877**	**-0,079815**	0,119094	0,101689
NA	42,4872	NA	**NA**	**-0,1406535**	**NA**	NA	0,119801
NA	50,248	51,0513	**NA**	**0,277136**	**0,289621**	NA	0,303947
NA	49,3147	NA	**NA**	**0,3415655**	**NA**	NA	0,4431
NA	NA	420,262	**NA**	**NA**	**-0,16358**	NA	NA
NA	26,4423	31,087	**NA**	**0,173881**	**0,1449925**	NA	0,15259
28,0017	NA	NA	**0,1430035**	**NA**	**NA**	0,124636	NA
NA	52,8767	45,3227	**NA**	**0,129186**	**0,098561**	NA	0,131707
71,8719	NA	101,015	**0,222776**	**NA**	**0,130409**	0,204698	NA
NA	NA	68,3608	**NA**	**NA**	**-0,187394**	NA	NA
NA	NA	51,2026	**NA**	**NA**	**0,1854755**	NA	NA
22,2346	NA	NA	**-0,2627305**	**NA**	**NA**	0,237191	NA
NA	NA	113,819	**NA**	**NA**	**0,122304**	NA	NA
NA	NA	42,7234	**NA**	**NA**	**0,1002395**	NA	NA
49,1474	45,0039	56,9731	**0,1523705**	**0,1162865**	**0,1812585**	0,139371	0,107953
NA	NA	56,9017	**NA**	**NA**	**-0,179793**	NA	NA
42,8765	55,7797	NA	**0,162225**	**0,1992405**	**NA**	0,1423	0,173482
336,403	434,271	538,73	**-0,1515145**	**-0,169951**	**-0,2302015**	0,137868	0,15365
NA	77,4942	NA	**NA**	**0,1585075**	**NA**	NA	0,206613
404,831	544,201	531,273	**0,24362195**	**0,24965785**	**0,2366495**	0,268923	0,27809
NA	NA	57,0245	**NA**	**NA**	**-0,232693**	NA	NA
98,3675	79,9207	NA	**0,1896005**	**0,119981**	**NA**	0,162277	0,103454
250,427	254,144	NA	**0,0622605**	**0,0565345**	**NA**	0,110124	0,101943
143,089	168,798	148,626	**-0,1229916**	**-0,1528126**	**-0,0982811**	0,181991	0,213716
61,5642	NA	NA	**0,123269**	**NA**	**NA**	0,116562	NA
41,6968	43,8354	54,1453	**0,1293835**	**0,165807**	**0,244659**	0,121151	0,15287
NA	68,1187	83,5452	**NA**	**-0,088909**	**-0,0969145**	NA	0,110065
NA	204,874	187,576	**NA**	**0,116182**	**0,0957955**	NA	0,160582
43,3938	NA	NA	**0,128756**	**NA**	**NA**	0,109748	NA
NA	31,864	NA	**NA**	**0,1402555**	**NA**	NA	0,119662
95,8979	NA	100,676	**-0,1155495**	**NA**	**-0,155006**	0,102255	NA
NA	51,3969	NA	**NA**	**0,1585815**	**NA**	NA	0,191999
NA	70,1582	68,2828	**NA**	**0,0973785**	**0,150895**	NA	0,131635
NA	51,3817	57,7961	**NA**	**-0,140175**	**-0,1309245**	NA	0,16804
NA	73,7357	87,9693	**NA**	**-0,0993147**	**-0,1088132**	NA	0,112838
NA	NA	25,805	**NA**	**NA**	**-0,14946**	NA	NA
45,1692	NA	NA	**0,1583955**	**NA**	**NA**	0,149858	NA
21,6537	20,4968	NA	**-0,2650695**	**-0,1608875**	**NA**	0,230925	0,143407
NA	70,2164	NA	**NA**	**0,116023**	**NA**	NA	0,168834
NA	83,4299	NA	**NA**	**-0,159998**	**NA**	NA	0,291587
NA	NA	89,254	**NA**	**NA**	**0,1863765**	NA	NA
NA	25,1857	NA	**NA**	**0,2033575**	**NA**	NA	0,173326
NA	37,8776	NA	**NA**	**-0,104602**	**NA**	NA	0,100714
61,7002	NA	NA	**-0,07688315**	**NA**	**NA**	0,100958	NA

NA	NA	99,8508	**NA**	**NA**	**0,097543**	NA	NA
NA	25,8556	NA	**NA**	**0,22334**	**NA**	NA	0,190498
NA	28,079	NA	**NA**	**0,1609835**	**NA**	NA	0,151243
39,656	NA	NA	**-0,120122**	**NA**	**NA**	0,116228	NA
NA	NA	30,6323	**NA**	**NA**	**-0,130462**	NA	NA
NA	33,9068	27,0712	**NA**	**0,1624955**	**0,3907**	NA	0,146362
NA	NA	49,2074	**NA**	**NA**	**0,22507**	NA	NA
34,5687	NA	NA	**0,038223**	**NA**	**NA**	0,126433	NA
NA	36,3285	NA	**NA**	**-0,170475**	**NA**	NA	0,147708
NA	161,123	NA	**NA**	**0,0935071**	**NA**	NA	0,116375
57,0094	71,1223	NA	**-0,1065565**	**-0,083307**	**NA**	0,146468	0,108634
NA	NA	68,3169	**NA**	**NA**	**0,26005**	NA	NA
NA	23,5768	27,2473	**NA**	**-0,265395**	**-0,3023595**	NA	0,234067
NA	NA	68,0443	**NA**	**NA**	**0,138861**	NA	NA
NA	70,5685	NA	**NA**	**-0,1579175**	**NA**	NA	0,159432
25,8603	NA	NA	**-0,156295**	**NA**	**NA**	0,145037	NA
NA	49,0297	NA	**NA**	**-0,2442903**	**NA**	NA	0,233221
NA	75,9763	NA	**NA**	**0,109734**	**NA**	NA	0,137922
69,9151	81,9256	NA	**0,168513**	**0,128833**	**NA**	0,212933	0,17267
41,2855	59,1602	63,4634	**0,212216**	**0,23612**	**0,1537995**	0,190091	0,210131
NA	131,487	NA	**NA**	**-0,06568285**	**NA**	NA	0,112144
23,3928	20,9857	NA	**-0,25018865**	**-0,19034265**	**NA**	0,222116	0,171875
NA	60,0864	NA	**NA**	**0,262039**	**NA**	NA	0,299019
NA	111,758	NA	**NA**	**-0,0762905**	**NA**	NA	0,11007
89,9128	NA	NA	**0,0707355**	**NA**	**NA**	0,103221	NA
NA	NA	41,1994	**NA**	**NA**	**-0,1449165**	NA	NA
NA	NA	70,4367	**NA**	**NA**	**-0,169059**	NA	NA
27,6943	NA	NA	**0,1120975**	**NA**	**NA**	0,108183	NA
32,8158	30,8039	25,7911	**-0,3788915**	**-0,2024845**	**-0,1893625**	0,33699	0,172909
38,5253	37,8301	25,5113	**-0,30043**	**-0,2945615**	**-0,3075515**	0,276861	0,272019
NA	NA	46,4536	**NA**	**NA**	**-0,098096**	NA	NA
NA	135,104	NA	**NA**	**0,09385665**	**NA**	NA	0,152926
NA	NA	94,8381	**NA**	**NA**	**0,1132755**	NA	NA
88,8102	84,7885	NA	**-0,1035198**	**-0,1159348**	**NA**	0,141764	0,155431
40,1361	50,1209	NA	**0,2209067**	**0,22007675**	**NA**	0,243856	0,242656
23,0669	27,861	NA	**0,1259555**	**0,1969945**	**NA**	0,113467	0,173881
27,8042	30,4333	NA	**0,2087955**	**0,3339385**	**NA**	0,204797	0,309435
164,652	NA	136,768	**-0,1173095**	**NA**	**-0,129458**	0,107546	NA
126,693	120,525	113,985	**-0,106997**	**-0,123441**	**-0,1891455**	0,108692	0,123966
NA	NA	31,1829	**NA**	**NA**	**0,2494725**	NA	NA
NA	85,8131	NA	**NA**	**0,1585945**	**NA**	NA	0,17547
NA	98,3754	76,5686	**NA**	**0,423889**	**0,276697**	NA	0,438771
NA	NA	108,084	**NA**	**NA**	**-0,0982825**	NA	NA
568,638	766,863	NA	**0,102191**	**0,1008085**	**NA**	0,112254	0,110556
84,7398	NA	118,894	**-0,0929295**	**NA**	**-0,0883125**	0,129857	NA
NA	NA	42,627	**NA**	**NA**	**-0,197985**	NA	NA
NA	199,212	182,415	**NA**	**-0,1391615**	**-0,197985**	NA	0,136637
38,7914	40,1274	NA	**-0,125886**	**-0,125203**	**NA**	0,122875	0,122149
NA	429,212	NA	**NA**	**-0,1053115**	**NA**	NA	0,101432
NA	19,7904	NA	**NA**	**0,215232**	**NA**	NA	0,190724
NA	NA	79,6157	**NA**	**NA**	**-0,1323705**	NA	NA
26,9753	40,8087	47,413	**-0,11388055**	**-0,1606442**	**-0,1579601**	0,109163	0,15046
121,298	154,231	97,9899	**0,1010926**	**0,07792945**	**0,10703646**	0,163455	0,116134
NA	66,778	74,9624	**NA**	**-0,171701**	**-0,2178395**	NA	0,14886
90,4396	98,8644	112,892	**-0,131613**	**-0,14234835**	**-0,1224305**	0,144702	0,154975
49,2145	63,6358	NA	**0,1958525**	**0,327875**	**NA**	0,177802	0,290631
NA	166,376	NA	**NA**	**0,09360105**	**NA**	NA	0,128039
NA	NA	122,202	**NA**	**NA**	**-0,1964365**	NA	NA
2775,83	6604,79	2818,02	**-0,15460275**	**-0,15901425**	**-0,22463275**	0,168251	0,172376
183,539	NA	NA	**0,053935**	**NA**	**NA**	0,10354	NA
43,8392	37,5153	NA	**-0,1734245**	**-0,127695**	**NA**	0,18489	0,14184
73,7483	NA	NA	**-0,0989375**	**NA**	**NA**	0,132157	NA
80,8149	139,525	128,432	**-0,1552995**	**-0,1665265**	**-0,1092325**	0,159347	0,172884
NA	NA	37,399	**NA**	**NA**	**-0,178364**	NA	NA
NA	NA	56,5361	**NA**	**NA**	**0,1172**	NA	NA
NA	94,5653	NA	**NA**	**-0,157858**	**NA**	NA	0,142612

23,9116	41,297	NA	**0,164822**	**0,128023**	**NA**	0,147366	0,116412
NA	NA	37,3823	**NA**	**NA**	**-0,1479525**	NA	NA
NA	NA	43,1575	**NA**	**NA**	**-0,308055**	NA	NA
73,8698	NA	NA	**-0,1064705**	**NA**	**NA**	0,102509	NA
38,7383	35,5955	55,8253	**0,1402415**	**0,181612**	**0,184478**	0,137998	0,185049
NA	NA	122,177	**NA**	**NA**	**-0,103541**	NA	NA
105,694	NA	164,525	**-0,118932**	**NA**	**-0,145038**	0,134126	NA
NA	NA	32,9042	**NA**	**NA**	**-0,203153**	NA	NA
NA	NA	22,8188	**NA**	**NA**	**0,210553**	NA	NA
NA	27,0146	NA	**NA**	**-0,122357**	**NA**	NA	0,106657
32,4882	NA	37,9981	**0,12171**	**NA**	**0,141201**	0,104567	NA
37,5843	NA	NA	**0,107598**	**NA**	**NA**	0,103879	NA
63,3213	61,2203	93,9818	**-0,0979075**	**-0,1521195**	**-0,1578095**	0,101684	0,167605
205,674	267,612	290,318	**-0,19570335**	**-0,15566885**	**-0,14621335**	0,226061	0,188114
NA	NA	40,4764	**NA**	**NA**	**0,14806**	NA	NA
NA	NA	183,994	**NA**	**NA**	**0,08233285**	NA	NA
42,2735	20,54	NA	**-0,1830215**	**-0,204721**	**NA**	0,155948	0,174718
NA	82,2913	NA	**NA**	**0,118115**	**NA**	NA	0,100869
NA	NA	48,9445	**NA**	**NA**	**-0,0879055**	NA	NA
NA	187,359	NA	**NA**	**0,0716375**	**NA**	NA	0,103292
41,5356	NA	35,1478	**-0,20243**	**NA**	**-0,2007965**	0,183465	NA
160,92	233,118	78,9138	**0,1549085**	**0,1198915**	**0,2038955**	0,167334	0,133826
NA	103,36	60,0892	**NA**	**-0,096539**	**-0,007882**	NA	0,104126
84,124	102,617	121,687	**-0,07628915**	**-0,07066265**	**-0,09389865**	0,118391	0,111202
NA	30,3078	55,3238	**NA**	**-0,2074745**	**-0,1944555**	NA	0,184496
NA	NA	97,41	**NA**	**NA**	**0,1010895**	NA	NA
341,462	NA	NA	**0,124592**	**NA**	**NA**	0,130614	NA
250,373	387,084	NA	**0,1380275**	**0,1216425**	**NA**	0,125003	0,11083
29,1155	33,6622	34,2298	**-0,0474215**	**-0,040346**	**-0,091039**	0,223596	0,140917
65,0617	NA	63,0002	**0,1903995**	**NA**	**0,1478845**	0,172755	NA
NA	78,7529	NA	**NA**	**0,107349**	**NA**	NA	0,124644
89,4992	119,69	NA	**0,204931**	**0,147161**	**NA**	0,199698	0,148568
89,7842	116,332	NA	**-0,1809831**	**-0,16430515**	**NA**	0,179058	0,164238
101,771	NA	NA	**0,1642355**	**NA**	**NA**	0,143461	NA
NA	34,8956	NA	**NA**	**0,1218545**	**NA**	NA	0,104326
161,708	265,666	NA	**0,164887**	**0,1096945**	**NA**	0,186316	0,13187
NA	36,7869	NA	**NA**	**0,1321495**	**NA**	NA	0,112434
NA	NA	29,3853	**NA**	**NA**	**0,263398**	NA	NA
NA	32,0337	NA	**NA**	**-0,1554175**	**NA**	NA	0,121475
NA	NA	203,481	**NA**	**NA**	**-0,0616171**	NA	NA
NA	28,322	23,8141	**NA**	**-0,204682**	**-0,290823**	NA	0,174529
NA	NA	88,7915	**NA**	**NA**	**0,1663115**	NA	NA
NA	NA	163,809	**NA**	**NA**	**0,1310825**	NA	NA
NA	NA	46,5136	**NA**	**NA**	**-0,1915795**	NA	NA
NA	171,776	165,497	**NA**	**0,1317365**	**0,23532**	NA	0,223831
NA	NA	77,669	**NA**	**NA**	**-0,1777845**	NA	NA
NA	NA	15,892	**NA**	**NA**	**-0,1514225**	NA	NA
NA	30,1622	22,8478	**NA**	**-0,146483**	**-0,1142405**	NA	0,140051
NA	NA	78,7542	**NA**	**NA**	**-0,228168**	NA	NA
38,3609	41,4746	NA	**0,1930505**	**0,1727765**	**NA**	0,167535	0,149258
NA	54,8248	NA	**NA**	**0,1913475**	**NA**	NA	0,177638
NA	51,7587	NA	**NA**	**-0,0903562**	**NA**	NA	0,115994
57,8436	NA	NA	**-0,1104845**	**NA**	**NA**	0,114176	NA
NA	NA	199,213	**NA**	**NA**	**-0,066431**	NA	NA
49,2816	NA	47,8373	**0,1249505**	**NA**	**0,1392165**	0,117499	NA
127,2	NA	NA	**0,0958635**	**NA**	**NA**	0,115865	NA
NA	NA	26,9833	**NA**	**NA**	**-0,1101395**	NA	NA
58,7078	61,7183	62,0593	**-0,3194055**	**-0,41233**	**-0,3650135**	0,281414	0,378611
NA	NA	71,119	**NA**	**NA**	**0,1969885**	NA	NA
49,6892	73,5606	50,2537	**-0,1641165**	**-0,2020055**	**-0,200036**	0,150511	0,189087
24,4891	35,5737	NA	**0,404409**	**0,1083065**	**NA**	0,361969	0,110211
NA	115,071	55,6012	**NA**	**0,1520395**	**0,247872**	NA	0,28379
NA	34,7342	NA	**NA**	**-0,14151**	**NA**	NA	0,121926
NA	NA	38,6857	**NA**	**NA**	**-0,1059255**	NA	NA
NA	61,2846	39,0262	**NA**	**-0,2198645**	**-0,1351705**	NA	0,187504
175,893	NA	NA	**-0,0943315**	**NA**	**NA**	0,118572	NA

85,9243	162,991	NA	**-0,1320815**	**-0,156114**	**NA**	0,123048	0,147459
NA	73,8231	NA	**NA**	**0,1757935**	**NA**	NA	0,154045
22,9489	30,6114	NA	**0,1461345**	**0,1445725**	**NA**	0,128507	0,127186
NA	NA	59,3553	**NA**	**NA**	**-0,1347405**	NA	NA
25,7302	NA	NA	**0,239157**	**NA**	**NA**	0,259636	NA
NA	NA	452,262	**NA**	**NA**	**0,0876335**	NA	NA
106,799	122,089	NA	**0,2995775**	**0,1918585**	**NA**	0,267437	0,165796
NA	110,636	NA	**NA**	**0,09356**	**NA**	NA	0,140185
NA	67,2184	69,2067	**NA**	**0,1710475**	**-0,175283**	NA	0,171686
NA	NA	176,907	**NA**	**NA**	**-0,1434725**	NA	NA
NA	128,954	140,132	**NA**	**0,069459**	**0,107134**	NA	0,10506
57,2392	NA	NA	**-0,083085**	**NA**	**NA**	0,103346	NA
91,1669	NA	NA	**0,1348595**	**NA**	**NA**	0,12228	NA
36,4662	NA	NA	**0,1172995**	**NA**	**NA**	0,104154	NA
NA	46,0939	NA	**NA**	**-0,258739**	**NA**	NA	0,234111
NA	NA	40,3939	**NA**	**NA**	**-0,283382**	NA	NA
NA	NA	66,8797	**NA**	**NA**	**-0,200934**	NA	NA
37,4845	NA	NA	**0,126995**	**NA**	**NA**	0,136687	NA
NA	NA	33,5815	**NA**	**NA**	**-0,282196**	NA	NA
25,772	NA	34,9088	**0,1355135**	**NA**	**0,2082675**	0,133682	NA
NA	266,727	NA	**NA**	**-0,0768405**	**NA**	NA	0,152131
NA	NA	86,646	**NA**	**NA**	**0,212558**	NA	NA
NA	NA	70,6458	**NA**	**NA**	**-0,0971208**	NA	NA
45,4309	NA	NA	**-0,0982**	**NA**	**NA**	0,118438	NA
NA	263,151	NA	**NA**	**-0,1343215**	**NA**	NA	0,163078
NA	264,857	NA	**NA**	**0,14543425**	**NA**	NA	0,157814
69,4336	NA	75,3329	**-0,1786065**	**NA**	**-0,127787**	0,158049	NA
47,5394	52,801	NA	**-0,14436**	**-0,135079**	**NA**	0,156949	0,145286
NA	36,6056	NA	**NA**	**-0,137865**	**NA**	NA	0,118013
NA	148,491	NA	**NA**	**0,1160555**	**NA**	NA	0,246187
27,3137	NA	NA	**0,127673**	**NA**	**NA**	0,126564	NA
NA	NA	40,9754	**NA**	**NA**	**-0,120152**	NA	NA
NA	85,8989	NA	**NA**	**-0,131339**	**NA**	NA	0,111781
46,4639	65,2701	NA	**0,265083**	**0,145134**	**NA**	0,251062	0,146982
NA	70,1867	38,6467	**NA**	**0,146365**	**0,1394595**	NA	0,130223
33,0455	NA	NA	**-0,1934405**	**NA**	**NA**	0,193682	NA
NA	NA	25,71	**NA**	**NA**	**-0,094907**	NA	NA
NA	NA	40,7378	**NA**	**NA**	**-0,205605**	NA	NA
NA	51,0413	NA	**NA**	**0,112183**	**NA**	NA	0,118224
NA	NA	62,2545	**NA**	**NA**	**0,042706**	NA	NA
NA	NA	51,4222	**NA**	**NA**	**-0,2461775**	NA	NA
NA	30,7775	38,0929	**NA**	**-0,1384155**	**-0,119961**	NA	0,122394
21,5222	NA	NA	**0,1792205**	**NA**	**NA**	0,153849	NA
20,0353	25,0777	NA	**0,234326**	**0,1493735**	**NA**	0,207359	0,13551
NA	NA	69,9273	**NA**	**NA**	**-0,12924705**	NA	NA
169,904	NA	NA	**0,094454**	**NA**	**NA**	0,106484	NA
57,8762	48,1294	NA	**-0,0967535**	**-0,1876465**	**NA**	0,103308	0,187265
64,1578	NA	NA	**-0,1045275**	**NA**	**NA**	0,131082	NA
70,3779	91,0314	NA	**0,1481535**	**0,0977515**	**NA**	0,170811	0,119743
34,6872	46,6899	NA	**0,221748**	**0,141993**	**NA**	0,211826	0,129632
198,587	224,61	NA	**0,0792695**	**0,078338**	**NA**	0,137138	0,135912
63,8681	82,0655	NA	**0,2054655**	**0,1108065**	**NA**	0,218549	0,129035
104,962	127,967	138,035	**0,1143665**	**0,122103**	**0,110257**	0,117639	0,124887
128,108	216,309	110,897	**0,2273575**	**0,217374**	**0,1231665**	0,198637	0,189396
22,4797	NA	26,1519	**-0,1343865**	**NA**	**-0,1286385**	0,114735	NA
39,2494	27,0798	NA	**-0,1162535**	**0,0781035**	**NA**	0,171126	0,104455
NA	NA	156,348	**NA**	**NA**	**-0,1308355**	NA	NA
102,663	161,902	147,452	**0,1471613**	**0,1109475**	**0,1011965**	0,17579	0,125524
NA	NA	76,4827	**NA**	**NA**	**-0,112848**	NA	NA
NA	52,9119	NA	**NA**	**0,1614515**	**NA**	NA	0,152974
NA	115,995	NA	**NA**	**-0,172306**	**NA**	NA	0,147221
50,1059	NA	NA	**-0,2298735**	**NA**	**NA**	0,204943	NA
NA	187,325	122,992	**NA**	**0,108418**	**0,1578155**	NA	0,180012
NA	NA	154,374	**NA**	**NA**	**0,0683525**	NA	NA
NA	197,135	185,412	**NA**	**0,097227**	**0,0683525**	NA	0,143094
NA	NA	108,715	**NA**	**NA**	**-0,08340115**	NA	NA

NA	170,691	NA	**NA**	**-0,1652915**	**NA**	NA	0,148695
NA	185,285	NA	**NA**	**-0,085987**	**NA**	NA	0,124399
NA	107,641	NA	**NA**	**0,087328**	**NA**	NA	0,10966
NA	223,301	190,865	**NA**	**0,097725**	**0,1329675**	NA	0,109303
NA	33,5363	28,5785	**NA**	**-0,116178**	**-0,112286**	NA	0,104325
NA	30,7144	NA	**NA**	**0,130852**	**NA**	NA	0,143184
NA	NA	77,7466	**NA**	**NA**	**-0,132919**	NA	NA
NA	34,3312	21,4521	**NA**	**-0,166983**	**-0,199358**	NA	0,142398
NA	NA	79,2515	**NA**	**NA**	**0,1362945**	NA	NA
30,3074	NA	NA	**0,1833125**	**NA**	**NA**	0,202499	NA
NA	NA	31,2434	**NA**	**NA**	**-0,1257845**	NA	NA
NA	NA	158,51	**NA**	**NA**	**0,0824315**	NA	NA
45,0432	NA	NA	**0,141062**	**NA**	**NA**	0,172051	NA
NA	98,2692	85,0072	**NA**	**-0,117589**	**-0,2141625**	NA	0,10071
82,3534	NA	NA	**0,11606**	**NA**	**NA**	0,138036	NA
NA	NA	79,5432	**NA**	**NA**	**-0,1349685**	NA	NA
NA	30,3582	NA	**NA**	**0,233791**	**NA**	NA	0,268303
NA	NA	24,0776	**NA**	**NA**	**-0,1550835**	NA	NA
62,6249	45,337	43,1512	**0,117568**	**0,1513835**	**0,1401195**	0,102768	0,133671
NA	NA	41,0383	**NA**	**NA**	**0,298612**	NA	NA
24,8816	24,9537	19,041	**0,2331505**	**0,3725025**	**0,3593185**	0,248835	0,364459
NA	NA	35,7301	**NA**	**NA**	**0,150759**	NA	NA
42,905	NA	42,0767	**0,233867**	**NA**	**0,2332475**	0,202631	NA
23,5521	NA	NA	**-0,1998365**	**NA**	**NA**	0,171666	NA
NA	NA	45,9597	**NA**	**NA**	**0,1984625**	NA	NA
NA	NA	40,7616	**NA**	**NA**	**-0,249627**	NA	NA
NA	NA	28,5069	**NA**	**NA**	**-0,1237855**	NA	NA
NA	126,642	123,609	**NA**	**0,141019**	**0,2426295**	NA	0,272711
NA	48,4441	54,815	**NA**	**-0,1253295**	**-0,216164**	NA	0,108257
NA	58,1115	NA	**NA**	**-0,1218885**	**NA**	NA	0,105321
43,2487	NA	NA	**-0,1205775**	**NA**	**NA**	0,115392	NA
NA	NA	409,57	**NA**	**NA**	**0,1276955**	NA	NA
78,5103	105,537	NA	**-0,127376**	**-0,143976**	**NA**	0,13537	0,155877
NA	NA	22,9543	**NA**	**NA**	**0,329694**	NA	NA
90,21	115,156	106,124	**-0,154773**	**-0,2009795**	**-0,204676**	0,138322	0,18321
53,8296	55,7024	47,5603	**0,135095**	**0,149609**	**0,121549**	0,121408	0,135265
30,4847	NA	36,8311	**-0,1226575**	**NA**	**-0,2217435**	0,113664	NA
NA	49,3705	NA	**NA**	**-0,15333**	**NA**	NA	0,148159
62,6309	NA	NA	**-0,126672**	**NA**	**NA**	0,112288	NA
147,539	NA	NA	**-0,1813075**	**NA**	**NA**	0,172751	NA
123,553	192,342	159,628	**-0,06892055**	**-0,06352545**	**-0,0628666**	0,116323	0,108972
139,444	199,016	186,809	**0,1575025**	**0,150105**	**0,1720785**	0,192418	0,185119
NA	55,8357	NA	**NA**	**-0,1565765**	**NA**	NA	0,166644
50,4093	NA	NA	**0,106948**	**NA**	**NA**	0,15101	NA
NA	NA	41,4952	**NA**	**NA**	**-0,149616**	NA	NA
NA	NA	37,7841	**NA**	**NA**	**-0,1428865**	NA	NA
59,9041	NA	NA	**0,1354075**	**NA**	**NA**	0,213637	NA
NA	54,2701	NA	**NA**	**-0,278293**	**NA**	NA	0,260147
NA	71,543	NA	**NA**	**-0,1082681**	**NA**	NA	0,182268
NA	55,1219	NA	**NA**	**0,12735**	**NA**	NA	0,133045
NA	62,4007	NA	**NA**	**0,137642**	**NA**	NA	0,117859
NA	153,833	123,855	**NA**	**-0,1274045**	**-0,135593**	NA	0,110046
NA	26,1114	NA	**NA**	**0,215798**	**NA**	NA	0,19874
NA	NA	62,9383	**NA**	**NA**	**-0,1123695**	NA	NA
20,213	NA	13,0158	**-0,136076**	**NA**	**-0,457586**	0,118734	NA
123,991	NA	NA	**-0,124343**	**NA**	**NA**	0,105772	NA
70,8493	112,968	57,6267	**-0,146675**	**-0,122443**	**-0,1577855**	0,126007	0,10552
NA	NA	67,5532	**NA**	**NA**	**-0,175524**	NA	NA
44,9153	50,515	NA	**0,301469**	**0,1918215**	**NA**	0,258105	0,164109
NA	31,031	NA	**NA**	**-0,28031985**	**NA**	NA	0,288185
42,2959	29,7137	56,1618	**0,272335**	**0,2998415**	**0,228137**	0,253233	0,283736
NA	280,897	222,31	**NA**	**-0,126685**	**-0,149209**	NA	0,144161
41,4347	52,1188	NA	**-0,07568885**	**-0,10050055**	**NA**	0,103865	0,13216
35,4843	NA	NA	**0,102483**	**NA**	**NA**	0,102229	NA
NA	NA	96,8496	**NA**	**NA**	**-0,118279**	NA	NA
18,5316	23,1797	NA	**0,2103155**	**0,1463515**	**NA**	0,184285	0,130257

NA	NA	57,8083	**NA**	**NA**	**-0,192517**	NA	NA
NA	NA	38,701	**NA**	**NA**	**-0,2288375**	NA	NA
52,7131	90,0341	69,9766	**-0,2248975**	**-0,154504**	**-0,2288375**	0,193056	0,131593
NA	NA	7523,01	**NA**	**NA**	**0,06503745**	NA	NA
NA	43,408	39,8473	**NA**	**0,26798**	**0,229659**	NA	0,247145
NA	98,7804	90,0123	**NA**	**-0,1154765**	**-0,1267935**	NA	0,104965
NA	20,457	NA	**NA**	**-0,1776305**	**NA**	NA	0,152458
55,0694	NA	NA	**0,1542335**	**NA**	**NA**	0,182431	NA
NA	NA	115,673	**NA**	**NA**	**0,235197**	NA	NA
NA	NA	131,608	**NA**	**NA**	**0,1011995**	NA	NA
NA	95,1773	77,0562	**NA**	**-0,1246495**	**-0,151006**	NA	0,117002
140,434	161,094	163,071	**-0,090602**	**-0,11256965**	**-0,09296965**	0,148147	0,174367
NA	89,8364	77,4794	**NA**	**-0,1472505**	**-0,0925035**	NA	0,152464
NA	138,751	NA	**NA**	**0,061611**	**NA**	NA	0,100651
43,114	55,0687	NA	**0,207544**	**0,209818**	**NA**	0,179043	0,180956
NA	30,7782	32,4528	**NA**	**0,224135**	**0,130343**	NA	0,194101
NA	41,6519	NA	**NA**	**-0,1734265**	**NA**	NA	0,154378
55,5784	71,1165	45,479	**-0,108207**	**-0,1705805**	**-0,1300985**	0,105429	0,174904
38,4795	79,2947	NA	**0,1715965**	**0,117033**	**NA**	0,182439	0,13072
NA	22,1689	25,9113	**NA**	**-0,1464335**	**-0,153399**	NA	0,134532
NA	NA	55,226	**NA**	**NA**	**0,137419**	NA	NA
25,1698	NA	NA	**0,103593**	**NA**	**NA**	0,103559	NA
NA	65,4136	NA	**NA**	**0,22232**	**NA**	NA	0,245107
NA	NA	22,4427	**NA**	**NA**	**0,1218445**	NA	NA
NA	31,0733	NA	**NA**	**0,107601**	**NA**	NA	0,10352
NA	25,3717	21,9336	**NA**	**-0,1222355**	**-0,2178795**	NA	0,104621
41,1463	NA	NA	**0,138808**	**NA**	**NA**	0,136399	NA
NA	271,752	NA	**NA**	**0,060702**	**NA**	NA	0,118816
NA	61,696	NA	**NA**	**0,1965815**	**NA**	NA	0,32563
17,617	NA	NA	**-0,288247**	**NA**	**NA**	0,250239	NA
76,6083	71,2214	83,4466	**-0,1208085**	**-0,1613965**	**-0,130511**	0,102734	0,137453
NA	66,6328	NA	**NA**	**0,238212**	**NA**	NA	0,224294
72,4011	NA	NA	**0,180907**	**NA**	**NA**	0,164934	NA
45,8556	NA	NA	**0,094965**	**NA**	**NA**	0,115328	NA
55,2891	NA	NA	**0,2133235**	**NA**	**NA**	0,340397	NA
27,3268	41,992	NA	**-0,2825385**	**-0,1245095**	**NA**	0,269431	0,130884
NA	21,9096	NA	**NA**	**0,226148**	**NA**	NA	0,193401
636,293	NA	NA	**0,0752265**	**NA**	**NA**	0,102385	NA
NA	19,9989	NA	**NA**	**0,170156**	**NA**	NA	0,146579
NA	483,292	NA	**NA**	**0,1331565**	**NA**	NA	0,113662
NA	NA	27,9231	**NA**	**NA**	**-0,2814123**	NA	NA
NA	NA	37,6549	**NA**	**NA**	**0,1071**	NA	NA
27,0843	43,4908	26,608	**0,140999**	**0,2872595**	**0,3833365**	0,131562	0,255232
NA	48,9444	NA	**NA**	**-0,1268625**	**NA**	NA	0,108292
45,9591	79,3395	NA	**-0,1815155**	**-0,186846**	**NA**	0,190624	0,197385
35,1305	NA	NA	**0,1810805**	**NA**	**NA**	0,154274	NA
NA	NA	52,1887	**NA**	**NA**	**-0,1653585**	NA	NA
NA	681,184	315,115	**NA**	**-0,0808075**	**-0,093117**	NA	0,106025
75,5974	NA	76,7315	**0,1345785**	**NA**	**0,1073435**	0,158427	NA
NA	NA	58,1622	**NA**	**NA**	**-0,1508285**	NA	NA
NA	NA	205,239	**NA**	**NA**	**0,308701**	NA	NA
29,9151	NA	36,8815	**-0,1339295**	**NA**	**-0,108567**	0,126945	NA
27,0756	61,5778	NA	**-0,207053**	**-0,15707**	**NA**	0,179173	0,13466
NA	NA	34,7887	**NA**	**NA**	**-0,229555**	NA	NA

sJSD_PNN	chr	gene_start	gene_stop	gene_id	gene_name	strand
NA	chr11	129939732	130014699	ENSG00000084234	APLP2	+
NA	chr11	35160417	35253949	ENSG00000026508	CD44	+
NA	chr11	35160417	35253949	ENSG00000026508	CD44	+
NA	chr11	57520715	57587018	ENSG00000198561	CTNND1	+
NA	chr12	56551945	56557280	ENSG00000092841	MYL6	+
NA	chr14	21677295	21737653	ENSG00000092199	HNRNPC	-
NA	chr14	23527773	23564823	ENSG00000100813	ACIN1	-
NA	chr17	16245848	16256970	ENSG00000166582	CENPV	-
0,194167	chr17	16342136	16381992	ENSG00000175061	FAM211A-A!	+
0,194167	chr17	16342136	16381992	ENSG00000175061	FAM211A-A!	+
0,143155	chr17	79976578	79981983	ENSG00000169689	STRA13	-
0,231623	chr19	10982189	11033453	ENSG00000142453	CARM1	+
0,187385	chr19	1285890	1378430	ENSG00000160953	MUM1	+
0,208909	chr19	50392911	50432796	ENSG00000104951	IL4I1	-
0,208909	chr19	50392911	50432796	ENSG00000104951	IL4I1	-
0,218191	chr1	207925402	207968858	ENSG00000117335	CD46	+
0,364381	chr1	234527059	234614849	ENSG00000059588	TARBP1	-
0,154413	chr1	46049518	46084566	ENSG00000132780	NASP	+
NA	chr1	54691105	54879152	ENSG00000157216	SSBP3	-
0,404516	chr1	89149905	89301938	ENSG00000065243	PKN2	+
NA	chr20	34291531	34330234	ENSG00000131051	RBM39	-
NA	chr21	27252861	27543446	ENSG00000142192	APP	-
NA	chr21	44299754	44333414	ENSG00000160194	NDUFV3	+
0,178882	chr22	29901868	29951205	ENSG00000100296	THOC5	-
0,294132	chr3	172468472	172539264	ENSG00000114346	ECT2	+
NA	chr3	183960089	183967336	ENSG00000214160	ALG3	-
NA	chr3	186500994	186507689	ENSG00000156976	EIF4A2	+
NA	chr3	186500994	186507689	ENSG00000156976	EIF4A2	+
NA	chr3	186500994	186507689	ENSG00000156976	EIF4A2	+
NA	chr3	33429828	33482863	ENSG00000153560	UBP1	-
NA	chr3	47844399	47891685	ENSG00000132153	DHX30	+
NA	chr5	179041179	179061785	ENSG00000169045	HNRNPH1	-
0,127444	chr5	271736	353971	ENSG00000249915	PDCD6	+
0,127444	chr5	271736	353971	ENSG00000249915	PDCD6	+
0,165533	chr5	70883115	70954531	ENSG00000131844	MCCC2	+
NA	chr6	30667584	30685666	ENSG00000137337	MDC1	-
NA	chr6	31606805	31620482	ENSG00000204463	BAG6	-
0,34783	chr7	134429003	134655479	ENSG00000122786	CALD1	+
NA	chr7	23544399	23571660	ENSG00000164548	TRA2A	-
NA	chr7	23544399	23571660	ENSG00000164548	TRA2A	-
0,123319	chr7	6414154	6443608	ENSG00000136238	RAC1	+
0,112604	chr9	131018108	131038274	ENSG00000167110	GOLGA2	-
0,440407	chr9	131314866	131395941	ENSG00000197694	SPTAN1	+
NA	chr9	34634719	34637806	ENSG00000147955	SIGMAR1	-
NA	chrX	119561682	119603220	ENSG00000005893	LAMP2	-
NA	chr10	104678050	104849978	ENSG00000148842	CNNM2	+
0,135645	chr10	104845940	104953056	ENSG00000076685	NT5C2	-
0,267329	chr10	120900279	120925179	ENSG00000183605	SFXN4	-
0,125034	chr10	126436718	126480439	ENSG00000203791	METTL10	-
0,116033	chr10	129894923	129924649	ENSG00000148773	MKI67	-
0,238193	chr10	27035522	27150016	ENSG00000136754	ABI1	-
0,345496	chr10	27793197	27831143	ENSG00000099246	RAB18	+
0,139145	chr10	32094365	32217742	ENSG00000165322	ARHGAP12	-
NA	chr10	34398488	35104253	ENSG00000148498	PARD3	-
0,151713	chr10	35415719	35501886	ENSG00000095794	CREM	+
NA	chr10	70090931	70102948	ENSG00000096746	HNRNPH3	+
0,161765	chr10	75196186	75255782	ENSG00000107758	PPP3CB	-
NA	chr10	75572259	75634343	ENSG00000148660	CAMK2G	-
NA	chr10	88853918	88951225	ENSG00000122376	FAM35A	+
NA	chr10	95066186	95242074	ENSG00000138119	MYOF	-
0,117973	chr10	95066186	95242074	ENSG00000138119	MYOF	-
0,149432	chr10	97423158	97453900	ENSG00000119977	TCTN3	-
0,138822	chr11	101981192	102104154	ENSG00000137693	YAP1	+

0,148754	chr11	101981192	102104154	ENSG00000137693	YAP1	+
NA	chr11	10818597	10830657	ENSG00000110321	EIF4G2	-
NA	chr11	118124118	118135251	ENSG00000149573	MPZL2	-
NA	chr11	12115543	12285334	ENSG00000133816	MICAL2	+
0,100911	chr11	122928197	122933938	ENSG00000109971	HSPA8	-
NA	chr11	33278218	33378569	ENSG00000110422	HIPK3	+
0,105389	chr11	36295051	36310999	ENSG00000110442	COMMD9	-
NA	chr11	47586888	47606114	ENSG00000213619	NDUFS3	+
NA	chr11	494512	507300	ENSG00000023191	RNH1	-
0,137497	chr11	62443970	62446567	ENSG00000162191	UBXN1	-
NA	chr11	64532078	64546258	ENSG00000168066	SF1	-
0,143325	chr11	66610306	66614017	ENSG00000173653	RCE1	+
0,130578	chr11	71713910	71791739	ENSG00000137497	NUMA1	-
NA	chr11	72975550	73009662	ENSG00000171631	P2RY6	+
0,137401	chr11	82970139	82997450	ENSG00000137500	CCDC90B	-
0,123783	chr11	85668727	85780924	ENSG00000073921	PICALM	-
0,184988	chr11	85668727	85780924	ENSG00000073921	PICALM	-
NA	chr11	8932686	8941631	ENSG00000166452	AKIP1	+
0,126038	chr11	95566046	95658479	ENSG00000087053	MTMR2	-
NA	chr12	104609557	104744061	ENSG00000198431	TXNRD1	+
NA	chr12	10851683	10875911	ENSG00000060138	YBX3	-
0,168442	chr12	109525996	109531436	ENSG00000189046	ALKBH2	-
0,111456	chr12	111157485	111180744	ENSG00000186298	PPP1CC	-
NA	chr12	111890018	112037480	ENSG00000204842	ATXN2	-
0,314433	chr12	112279782	112334343	ENSG00000089022	MAPKAPK5	+
NA	chr12	113658855	113736390	ENSG00000186815	TPCN1	+
0,178032	chr12	121458095	121477045	ENSG00000135114	OASL	-
0,128126	chr12	121675497	121736111	ENSG00000110931	CAMKK2	-
NA	chr12	1099675	1605099	ENSG00000082805	ERC1	+
0,102856	chr12	124808961	125052135	ENSG00000196498	NCOR2	-
0,124883	chr12	2966847	2986206	ENSG00000111206	FOXM1	-
0,174017	chr12	31226779	31257725	ENSG00000013573	DDX11	+
0,193506	chr12	32832134	32898486	ENSG00000087470	DNM1L	+
NA	chr12	389295	498620	ENSG00000073614	KDM5A	-
0,120468	chr12	42632249	42853517	ENSG00000134283	PPHLN1	+
0,104809	chr12	49047184	49076021	ENSG00000139620	KANSL2	-
0,164086	chr12	50478755	50494495	ENSG00000066117	SMARCD1	+
0,16819	chr12	51441745	51454207	ENSG00000050426	LETMD1	+
NA	chr12	53894705	53900215	ENSG00000139546	TARBP2	+
0,204476	chr12	54694986	54745633	ENSG00000111481	COPZ1	+
NA	chr12	56347889	56367101	ENSG00000185664	PMEL	-
0,258652	chr12	57057127	57082159	ENSG00000110958	PTGES3	-
0,228385	chr12	57489191	57525922	ENSG00000166888	STAT6	-
NA	chr12	58087738	58115340	ENSG00000135506	OS9	+
NA	chr12	6484211	6500733	ENSG00000111321	LTBR	+
0,15359	chr12	6679249	6716642	ENSG00000111642	CHD4	-
NA	chr12	6772426	6781235	ENSG00000219410	RP4-761J14	+
0,219534	chr12	6775643	6798738	ENSG00000126746	ZNF384	-
0,121721	chr12	6832907	6841041	ENSG00000111652	COPS7A	+
0,137581	chr12	7072408	7105520	ENSG00000126749	EMG1	+
NA	chr12	7341281	7371170	ENSG00000139197	PEX5	+
NA	chr12	95611522	95696566	ENSG00000028203	VEZT	+
0,139163	chr12	861759	1020618	ENSG00000060237	WNK1	+
NA	chr13	114145310	114204542	ENSG00000150403	TMCO3	+
0,188103	chr13	114239013	114295785	ENSG00000198176	TFDP1	+
0,154574	chr13	115047059	115071283	ENSG00000169062	UPF3A	+
0,122395	chr13	37523912	37574398	ENSG00000120697	ALG5	-
0,127888	chr14	102690837	102771537	ENSG00000080823	MOK	-
NA	chr14	105155943	105185942	ENSG00000203485	INF2	+
NA	chr14	105886159	105937066	ENSG00000182979	MTA1	+
NA	chr14	21463996	21467322	ENSG00000258471	RP11-84C1(	-
NA	chr14	23389720	23398794	ENSG00000100462	PRMT5	-
0,158785	chr14	56025790	56168244	ENSG00000126777	KTN1	+
NA	chr14	65472892	65569413	ENSG00000125952	MAX	-
NA	chr14	89029253	89079853	ENSG00000100722	ZC3H14	+
NA	chr14	93170152	93215047	ENSG00000100600	LGMN	-

0,16411	chr14	96967770	97033448	ENSG00000090060	PAPOLA	+
NA	chr15	101840818	102065405	ENSG00000140479	PCSK6	-
NA	chr15	41099284	41106767	ENSG00000166140	ZFYVE19	+
NA	chr15	66782473	66790151	ENSG00000174446	SNAPC5	-
0,114189	chr15	69591286	69700119	ENSG00000137819	PAQR5	+
0,338053	chr15	72533522	72565340	ENSG00000137817	PARP6	-
0,350509	chr15	76196200	76227609	ENSG00000167196	FBXO22	+
NA	chr15	91509270	91538859	ENSG00000198901	PRC1	-
NA	chr16	15068448	15233196	ENSG00000179889	PDXDC1	+
NA	chr16	2802330	2822539	ENSG00000167978	SRRM2	+
NA	chr16	29831715	29859355	ENSG00000013364	MVP	+
0,237396	chr16	29952206	29984373	ENSG00000149932	TMEM219	+
0,270378	chr16	46830519	46865323	ENSG00000155330	C16orf87	-
0,137303	chr16	47111614	47177908	ENSG00000171208	NETO2	-
NA	chr16	4853204	4897343	ENSG00000140632	GLYR1	-
NA	chr16	53467889	53525561	ENSG00000103479	RBL2	+
NA	chr16	54952775	54963101	ENSG00000245694	CRNDE	-
NA	chr16	57792129	57896957	ENSG00000140859	KIFC3	-
NA	chr16	68344877	68392466	ENSG00000132600	PRMT7	+
0,140463	chr16	69151913	69166487	ENSG00000168802	CHTF8	-
NA	chr16	70557691	70608820	ENSG00000189091	SF3B3	+
NA	chr16	71762913	71843104	ENSG00000166747	AP1G1	-
NA	chr16	718086	724174	ENSG00000140983	RHOT2	+
NA	chr16	87730091	87799598	ENSG00000104731	KLHDC4	-
NA	chr16	87730091	87799598	ENSG00000104731	KLHDC4	-
0,125608	chr16	88636789	88698374	ENSG00000158545	ZC3H18	+
0,145508	chr16	88781751	88851619	ENSG00000103335	PIEZO1	-
NA	chr16	89803957	89883065	ENSG00000187741	FANCA	-
0,161511	chr17	15932471	16121499	ENSG00000141027	NCOR1	-
0,282727	chr17	15932471	16121499	ENSG00000141027	NCOR1	-
0,101741	chr17	16945859	17120993	ENSG00000133030	MPRIP	+
NA	chr17	18148150	18162230	ENSG00000177731	FLII	-
0,243088	chr17	18148150	18162230	ENSG00000177731	FLII	-
NA	chr17	20902910	20947073	ENSG00000124422	USP22	-
NA	chr17	27400528	27507430	ENSG00000196535	MYO18A	-
NA	chr17	2680350	2941033	ENSG00000132359	RAP1GAP2	+
NA	chr17	29421945	29709134	ENSG00000196712	NF1	+
0,118122	chr17	30677136	30708905	ENSG00000010244	ZNF207	+
0,182601	chr17	30677136	30708905	ENSG00000010244	ZNF207	+
0,256256	chr17	35441923	35766909	ENSG00000132142	ACACA	-
NA	chr17	41102543	41116515	ENSG00000266967	AARSD1	-
0,320843	chr17	41102543	41116515	ENSG00000266967	AARSD1	-
0,104386	chr17	46125691	46138849	ENSG00000082641	NFE2L1	+
NA	chr17	47448102	47554350	ENSG00000262039	RP11-81K2.1	+
0,12198	chr17	48133332	48167845	ENSG00000005884	ITGA3	+
0,150993	chr17	5328459	5336196	ENSG00000263272	CTC-524C5.	-
0,190504	chr17	5328459	5336196	ENSG00000263272	CTC-524C5.	-
NA	chr17	5328459	5336196	ENSG00000263272	CTC-524C5.	-
NA	chr17	62495734	62504317	ENSG00000108654	DDX5	-
NA	chr17	65821640	65980494	ENSG00000171634	BPTF	+
0,152007	chr17	66243715	66253297	ENSG00000196704	AMZ2	+
0,148131	chr17	71203492	71232892	ENSG00000133193	FAM104A	-
0,17338	chr17	7120444	7128592	ENSG00000072778	ACADVL	+
0,187685	chr17	74077087	74117657	ENSG00000182473	EXOC7	-
0,135804	chr17	7486847	7496107	ENSG00000129255	MPDU1	+
NA	chr17	74864538	74946475	ENSG00000167889	MGAT5B	+
NA	chr17	75276651	75496678	ENSG00000184640	09-sept	+
0,183	chr17	7788124	7816078	ENSG00000170004	CHD3	+
0,231325	chr17	79476997	79490873	ENSG00000184009	ACTG1	-
NA	chr17	79890260	79900288	ENSG00000183010	PYCR1	-
NA	chr17	8377523	8534079	ENSG00000133026	MYH10	-
NA	chr18	670324	712676	ENSG00000132199	ENOSF1	-
0,107889	chr19	10362944	10395784	ENSG00000266978	CTD-2369P2	-
0,179326	chr19	1248552	1259142	ENSG00000167470	MIDN	+
0,128408	chr19	13215716	13228381	ENSG00000104907	TRMT1	-
NA	chr19	14491313	14519537	ENSG00000123146	CD97	+

NA	chr19	1609291	1652604	ENSG00000071564	TCF3	-
0,13616	chr19	17337013	17340028	ENSG00000099330	OCEL1	+
0,267257	chr19	18529674	18545372	ENSG00000130511	SSBP4	+
NA	chr19	19303008	19312678	ENSG00000064490	RFXANK	+
0,188483	chr19	30094924	30108144	ENSG00000105171	POP4	+
0,122419	chr19	34663409	34720420	ENSG00000257103	LSM14A	+
0,169871	chr19	3538259	3574288	ENSG00000161091	MFSD12	-
0,183555	chr19	36119932	36128588	ENSG00000126254	RBM42	+
0,200591	chr19	41098789	41135725	ENSG00000090006	LTBP4	+
NA	chr19	41098789	41135725	ENSG00000090006	LTBP4	+
0,121744	chr19	41937223	41946622	ENSG00000105341	ATP5SL	-
NA	chr19	41937223	41946622	ENSG00000105341	ATP5SL	-
0,175157	chr19	45135500	45222031	ENSG00000266903	CTB-171A8.	-
0,178783	chr19	47104331	47114050	ENSG00000160014	CALM3	+
0,182115	chr19	50168823	50177173	ENSG00000126453	BCL2L12	+
0,110206	chr19	51848423	51869672	ENSG00000105379	ETFB	-
NA	chr19	5158506	5340814	ENSG00000105426	PTPRS	-
NA	chr19	55964352	55973710	ENSG00000063241	ISOC2	-
0,109023	chr19	6212966	6279959	ENSG00000130382	MLLT1	-
NA	chr19	797075	812327	ENSG00000011304	PTBP1	+
0,182084	chr1	117117031	117210375	ENSG00000143061	IGSF3	-
0,21181	chr1	11994262	12035595	ENSG00000083444	PLOD1	+
0,125539	chr1	148555979	148596267	ENSG00000243452	NBPF15	+
0,139944	chr1	150190717	150208504	ENSG00000143401	ANP32E	-
0,172115	chr1	150459887	150480078	ENSG00000143374	TARS2	+
0,107035	chr1	151732119	151736040	ENSG00000143436	MRPL9	-
NA	chr1	154179182	154193104	ENSG00000143612	C1orf43	-
NA	chr1	154192655	154243986	ENSG00000143569	UBAP2L	+
0,161936	chr1	154192655	154243986	ENSG00000143569	UBAP2L	+
0,13633	chr1	155107820	155111329	ENSG00000169241	SLC50A1	+
NA	chr1	155225770	155232221	ENSG00000116521	SCAMP3	-
NA	chr1	15736391	15756839	ENSG00000142634	EFHD2	+
NA	chr1	160185505	160254920	ENSG00000132716	DCAF8	-
NA	chr1	162531323	162569627	ENSG00000117143	UAP1	+
NA	chr1	164524821	164868533	ENSG00000185630	PBX1	+
NA	chr1	167690429	167761156	ENSG00000197965	MPZL1	+
NA	chr1	175913967	176176629	ENSG00000143207	RFWD2	-
0,231083	chr1	181003067	181031074	ENSG00000153029	MR1	+
NA	chr1	184020811	184043346	ENSG00000198860	TSEN15	+
0,104331	chr1	201452658	201478584	ENSG00000159176	CSRP1	-
0,250558	chr1	211431719	211489727	ENSG00000117625	RCOR3	+
0,297664	chr1	225589204	225616627	ENSG00000143815	LBR	-
0,121684	chr1	225674537	225840844	ENSG00000154380	ENAH	-
0,169171	chr1	228294380	228297013	ENSG00000162910	MRPL55	-
0,32435	chr1	2323267	2336883	ENSG00000157916	RER1	+
0,152508	chr1	23345941	23410182	ENSG00000004487	KDM1A	+
0,13439	chr1	241753404	241840678	ENSG00000054277	OPN3	-
0,107117	chr1	242011269	242058450	ENSG00000174371	EXO1	+
0,19465	chr1	24291294	24307417	ENSG00000188529	SRSF10	-
NA	chr1	24958207	24999758	ENSG00000133226	SRRM1	+
NA	chr1	36621180	36646450	ENSG00000116871	MAP7D1	+
NA	chr1	37958176	37980375	ENSG00000163875	MEAF6	-
NA	chr1	37958176	37980375	ENSG00000163875	MEAF6	-
0,114046	chr1	38422647	38456593	ENSG00000183431	SF3A3	-
0,132029	chr1	39546988	39952849	ENSG00000127603	MACF1	+
NA	chr1	41445007	41478235	ENSG00000171793	CTPS1	+
0,100329	chr1	42642210	42801548	ENSG00000198815	FOXJ3	-
0,32727	chr1	47124366	47184824	ENSG00000159658	EFCAB14	-
0,177681	chr1	52042851	52254889	ENSG00000117859	OSBPL9	+
0,187027	chr1	53711217	53793742	ENSG00000157193	LRP8	-
NA	chr1	53711217	53793742	ENSG00000157193	LRP8	-
0,369629	chr1	54231133	54304533	ENSG00000058804	NDC1	-
NA	chr1	54411750	54433841	ENSG00000116212	LRRC42	+
0,108702	chr1	61330931	61928465	ENSG00000162599	NFIA	+
0,115359	chr1	62920399	63153969	ENSG00000116641	DOCK7	-
NA	chr1	70671365	70718735	ENSG00000116754	SRSF11	+

NA	chr1	75171170	75199092	ENSG00000116791	CRYZ	-
NA	chr1	75171170	75199092	ENSG00000116791	CRYZ	-
NA	chr1	85015289	85040163	ENSG00000117151	CTBS	-
0,118649	chr1	85715639	85725355	ENSG00000162642	C1orf52	-
NA	chr1	8412457	8877702	ENSG00000142599	RERE	-
0,141356	chr1	87328132	87380107	ENSG00000183291	15-sept	-
NA	chr1	9789084	9884584	ENSG00000171603	CLSTN1	-
NA	chr20	11871371	11907257	ENSG00000132640	BTBD3	+
0,151652	chr20	1422807	1454487	ENSG00000088833	NSFL1C	-
0,197203	chr20	30155510	30161066	ENSG00000230613	HM13-AS1	-
0,149889	chr20	32581452	32696114	ENSG00000125970	RALY	+
NA	chr20	34213953	34252878	ENSG00000214078	CPNE1	-
NA	chr20	34291531	34330234	ENSG00000131051	RBM39	-
NA	chr20	34679426	34820721	ENSG00000088367	EPB41L1	+
NA	chr20	34679426	34820721	ENSG00000088367	EPB41L1	+
0,272851	chr20	37049235	37063996	ENSG00000196756	SNHG17	-
0,212125	chr20	43160426	43252888	ENSG00000168734	PKIG	+
NA	chr20	43160426	43252888	ENSG00000168734	PKIG	+
0,241498	chr20	45837859	45985567	ENSG00000101040	ZMYND8	-
0,197518	chr20	5525085	5591672	ENSG00000125772	GPCPD1	-
NA	chr20	61273797	61317137	ENSG00000101187	SLCO4A1	+
0,339731	chr21	34876238	34915797	ENSG00000159131	GART	-
0,121144	chr22	24376133	24384680	ENSG00000184674	GSTT1	-
NA	chr22	24936406	24951903	ENSG00000138867	GUCD1	-
NA	chr22	29663998	29696515	ENSG00000182944	EWSR1	+
NA	chr22	29663998	29696515	ENSG00000182944	EWSR1	+
0,114757	chr22	29723669	29819168	ENSG00000100280	AP1B1	-
NA	chr22	46726772	46753237	ENSG00000100416	TRMU	+
NA	chr22	50883429	50913454	ENSG00000100241	SBF1	-
NA	chr2	99953816	100017789	ENSG00000158417	EIF5B	+
NA	chr2	101436614	101613291	ENSG00000170485	NPAS2	+
0,107291	chr2	102313312	102511149	ENSG00000071054	MAP4K4	+
NA	chr2	102313312	102511149	ENSG00000071054	MAP4K4	+
NA	chr2	127805603	127864931	ENSG00000136717	BIN1	-
0,124329	chr2	1635659	1748624	ENSG00000130508	PXDN	-
NA	chr2	187867947	188419390	ENSG00000224063	AC007319.1	+
0,123657	chr2	192109911	192290115	ENSG00000128641	MYO1B	+
0,206077	chr2	201170604	201346986	ENSG00000196141	SPATS2L	+
NA	chr2	201717732	201729422	ENSG00000013441	CLK1	-
0,119426	chr2	228189867	228222550	ENSG00000168958	MFF	+
0,36823	chr2	230628554	230787955	ENSG00000153827	TRIP12	-
0,105459	chr2	230628554	230787955	ENSG00000153827	TRIP12	-
NA	chr2	234118697	234204320	ENSG00000085978	ATG16L1	+
NA	chr2	236402733	237040444	ENSG00000157985	AGAP1	+
0,219522	chr2	27548716	27579868	ENSG00000115207	GTF3C2	-
NA	chr2	27994584	28210954	ENSG00000243147	MRPL33	+
NA	chr2	28112808	28561768	ENSG00000158019	BRE	+
NA	chr2	36583069	36778278	ENSG00000150938	CRIM1	+
NA	chr2	36758948	36779411	ENSG00000217075	AC007401.2	-
NA	chr2	61404553	61418338	ENSG00000173209	AHSA2	+
NA	chr2	70508494	70520903	ENSG00000143977	SNRPG	-
NA	chr2	74759541	74782817	ENSG00000115318	LOXL3	-
0,113767	chr2	9543604	9563676	ENSG00000119185	ITGB1BP1	-
0,105349	chr3	100211463	100296288	ENSG00000181458	TMEM45A	+
0,109867	chr3	107241783	107530171	ENSG00000114439	BBX	+
NA	chr3	107762145	107809872	ENSG00000196776	CD47	-
0,115647	chr3	111451344	111695364	ENSG00000144824	PHLDB2	+
0,113058	chr3	113435307	113465147	ENSG00000121579	NAA50	-
0,103651	chr3	124684554	124774802	ENSG00000173706	HEG1	-
NA	chr3	127291912	127317094	ENSG00000163870	TPRA1	-
NA	chr3	128888327	128902765	ENSG00000169714	CNBP	-
NA	chr3	130569439	130735556	ENSG00000017260	ATP2C1	+
0,234099	chr3	131181056	131221827	ENSG00000114686	MRPL3	-
0,101752	chr3	133292574	133309105	ENSG00000091527	CDV3	+
0,107837	chr3	133292574	133309105	ENSG00000091527	CDV3	+
0,142792	chr3	135969148	136056738	ENSG00000114054	PCCB	+

NA	chr3	145787227	145881440	ENSG00000152952	PLOD2	-
NA	chr3	154741913	154901497	ENSG00000196549	MME	+
NA	chr3	183547173	183602721	ENSG00000175193	PARL	-
0,143783	chr3	183852826	184402546	ENSG00000145191	EIF2B5	+
0,100961	chr3	196769431	197026171	ENSG00000075711	DLG1	-
NA	chr3	27414214	27525911	ENSG00000033867	SLC4A7	-
0,113856	chr3	33429828	33482863	ENSG00000153560	UBP1	-
0,169945	chr3	37094117	37225180	ENSG00000093167	LRRFIP2	-
0,20386	chr3	38144620	38178733	ENSG00000060971	ACAA1	-
NA	chr3	38144620	38178733	ENSG00000060971	ACAA1	-
0,123865	chr3	44956749	45017677	ENSG00000163812	ZDHHC3	-
0,102996	chr3	47892182	48130769	ENSG00000047849	MAP4	-
NA	chr3	49977440	50137478	ENSG00000004534	RBM6	+
0,182647	chr3	50192478	50226508	ENSG00000001617	SEMA3F	+
NA	chr3	50304990	50314977	ENSG00000012171	SEMA3B	+
0,176205	chr3	50337320	50349812	ENSG00000114378	HYAL1	-
NA	chr3	50388126	50395891	ENSG00000114395	CYB561D2	+
0,140949	chr3	52109269	52188706	ENSG00000164087	POC1A	-
0,123284	chr3	52435029	52444366	ENSG00000163930	BAP1	-
0,255651	chr3	57994127	58157982	ENSG00000136068	FLNB	+
0,353873	chr3	78646390	79816965	ENSG00000169855	ROBO1	-
0,128853	chr3	9439299	9520924	ENSG00000168137	SETD5	+
0,202112	chr4	122722472	122738176	ENSG00000123737	EXOSC9	+
NA	chr4	152023903	152246784	ENSG00000109686	SH3D19	-
0,266948	chr4	39289076	39367995	ENSG00000035928	RFC1	-
0,213488	chr4	41361624	41702061	ENSG00000064042	LIMCH1	+
0,114762	chr4	48343339	48428229	ENSG00000109171	SLAIN2	+
0,365288	chr4	48807229	48863834	ENSG00000109180	OCIAD1	+
0,184985	chr4	54243810	55161439	ENSG00000145216	FIP1L1	+
NA	chr4	54243810	55161439	ENSG00000145216	FIP1L1	+
NA	chr4	54243810	55161439	ENSG00000145216	FIP1L1	+
0,142242	chr4	83343717	83351294	ENSG00000152795	HNRNPDL	-
NA	chr4	84377085	84390888	ENSG00000163319	MRPS18C	+
0,303371	chr5	10226442	10250009	ENSG00000150756	FAM173B	-
0,186913	chr5	102089685	102366809	ENSG00000145730	PAM	+
0,108658	chr5	137475455	137514675	ENSG00000112983	BRD8	-
0,219428	chr5	139781399	139929163	ENSG00000131503	ANKHD1	+
NA	chr5	139781399	139929163	ENSG00000131503	ANKHD1	+
NA	chr5	149737202	149779871	ENSG00000070814	TCOF1	+
NA	chr5	150480273	150537443	ENSG00000197043	ANXA6	-
0,108061	chr5	150480273	150537443	ENSG00000197043	ANXA6	-
0,206526	chr5	150480273	150537443	ENSG00000197043	ANXA6	-
NA	chr5	173034517	173043663	ENSG00000145919	BOD1	-
NA	chr5	179289066	179334859	ENSG00000197226	TBC1D9B	-
0,133249	chr5	31400604	31532303	ENSG00000113360	DROSHA	-
0,132817	chr5	36606457	36688436	ENSG00000079215	SLC1A3	+
NA	chr5	40759481	40798476	ENSG00000132356	PRKAA1	-
NA	chr5	43289497	43313614	ENSG00000112972	HMGCS1	-
NA	chr5	54603588	54721409	ENSG00000039123	SKIV2L2	+
NA	chr5	56469775	56560506	ENSG00000062194	GPBP1	+
NA	chr5	56469775	56560506	ENSG00000062194	GPBP1	+
0,116974	chr5	58264865	59817947	ENSG00000113448	PDE4D	-
NA	chr5	64920543	64962060	ENSG00000113597	TRAPPC13	+
0,10098	chr5	7851299	7906138	ENSG00000124275	MTRR	+
0,527731	chr5	95297705	95966789	ENSG00000251314	CTD-2337A1	+
NA	chr6	125541108	125623282	ENSG00000111906	HDDC2	-
0,135389	chr6	125541108	125623282	ENSG00000111906	HDDC2	-
0,160488	chr6	125541108	125623282	ENSG00000111906	HDDC2	-
NA	chr6	131160487	131384462	ENSG00000079819	EPB41L2	-
NA	chr6	168227602	168372703	ENSG00000130396	MLLT4	+
0,207239	chr6	24403153	24425810	ENSG00000124532	MRS2	+
0,175622	chr6	2988221	3019996	ENSG00000124588	NQO2	+
NA	chr6	30844198	30867933	ENSG00000204580	DDR1	+
NA	chr6	31847536	31865464	ENSG00000204371	EHMT2	-
0,114688	chr6	32936437	32949282	ENSG00000204256	BRD2	+
NA	chr6	41001366	41065526	ENSG00000124596	OARD1	-

0,166297	chr6	42896938	42907025	ENSG00000137161	CNPY3	+
0,257174	chr6	43737921	43754224	ENSG00000112715	VEGFA	+
0,196825	chr6	43737921	43754224	ENSG00000112715	VEGFA	+
0,101279	chr6	74225473	74233520	ENSG00000156508	EEF1A1	-
0,215033	chr6	76311225	76427997	ENSG00000112701	SENP6	+
0,115846	chr7	141438121	141487722	ENSG00000106028	SSBP1	+
NA	chr7	142952357	142984473	ENSG00000231840	AC073342.1	-
NA	chr7	150773711	150777953	ENSG00000164896	FASTK	-
0,207364	chr7	158523686	158622944	ENSG00000117868	ESYT2	-
0,156861	chr7	26229547	26241149	ENSG00000122566	HNRNPA2B1	-
0,144031	chr7	30536237	30591095	ENSG00000006625	GGCT	-
0,151075	chr7	35840542	35944917	ENSG00000122545	07-sept	+
0,100275	chr7	36429415	36493400	ENSG00000011426	ANLN	+
NA	chr7	45139699	45151646	ENSG00000136270	TBRG4	-
NA	chr7	48128225	48148330	ENSG00000183696	UPP1	+
0,111182	chr7	65425671	65447301	ENSG00000169919	GUSB	-
NA	chr7	855528	936072	ENSG00000164828	SUN1	+
0,128825	chr7	99933727	99965454	ENSG00000272752	STAG3L5P-I	+
NA	chr8	124191200	124222314	ENSG00000147689	FAM83A	+
0,141424	chr8	128806779	129113499	ENSG00000249859	PVT1	+
0,210701	chr8	130851839	131029375	ENSG00000153310	FAM49B	-
NA	chr8	134249414	134314265	ENSG00000104419	NDRG1	-
NA	chr8	144873090	144897549	ENSG00000180900	SCRIB	-
0,10596	chr8	17780349	17885478	ENSG00000078674	PCM1	+
NA	chr8	23101150	23119512	ENSG00000147457	CHMP7	+
0,185823	chr8	42195972	42229326	ENSG00000070501	POLB	+
NA	chr8	87480486	87526586	ENSG00000176623	RMDN1	-
NA	chr9	101866320	101916474	ENSG00000106799	TGFBR1	+
NA	chr9	111777432	111882225	ENSG00000106771	TMEM245	-
NA	chr9	112137746	112260590	ENSG00000070159	PTPN3	-
0,111004	chr9	128199672	128469513	ENSG00000119487	MAPKAP1	-
NA	chr9	132815705	132902448	ENSG00000148358	GPR107	+
NA	chr9	134269480	134375584	ENSG00000130723	PRRC2B	+
NA	chr9	139933922	139940655	ENSG00000107281	NPDC1	-
NA	chr9	5765076	5833117	ENSG00000099219	ERMP1	-
NA	chr9	6413151	6507054	ENSG00000147854	UHRF2	+
NA	chr9	80850978	80894606	ENSG00000148019	CEP78	+
NA	chr9	86582998	86595569	ENSG00000165119	HNRNPK	-
NA	chr9	99401859	99417585	ENSG00000158122	AAED1	-
NA	chrX	102930424	102943086	ENSG00000123562	MORF4L2	-
0,279348	chrX	106307650	106362057	ENSG00000089682	RBM41	-
0,108577	chrX	123094062	123556514	ENSG00000101972	STAG2	+
0,335022	chrX	128673826	128726538	ENSG00000122126	OCRL	+
NA	chrX	134021656	134049297	ENSG00000101928	MOSPD1	-
NA	chrX	13707244	13728625	ENSG00000123595	RAB9A	+
NA	chrX	13752832	13787480	ENSG00000046651	OFD1	+
0,17163	chrX	146993469	147032645	ENSG00000102081	FMR1	+
0,125506	chrX	153126969	153174677	ENSG00000198910	L1CAM	-
0,130673	chrX	23720370	23784592	ENSG00000123130	ACOT9	-
0,131946	chrX	23720370	23784592	ENSG00000123130	ACOT9	-
0,276693	chrX	31115794	33357558	ENSG00000198947	DMD	-
0,101411	chrX	39909068	40036582	ENSG00000183337	BCOR	-
NA	chrX	48929385	48937546	ENSG00000250232	AF196779.1	-
0,197871	chrX	9431335	9687780	ENSG00000101849	TBL1X	+

biotype	trans_id	length_trans	length_CDS	length_5UTR	length_3UTR	ExonNb
protein_coding	ENST00000263574	3720	2289	72	1356	18
protein_coding	ENST00000428726	3046	2226	123	694	18
protein_coding	ENST00000428726	3046	2226	123	694	18
protein_coding	ENST00000399050	6313	2904	536	2870	21
protein_coding	ENST00000551954	1193	0	0	0	2
protein_coding	ENST00000420743	1559	918	521	117	11
protein_coding	ENST00000262710	4935	4023	328	581	19
protein_coding	ENST00000299736	1165	816	63	283	5
processed_transcrip	ENST00000484836	821	0	0	0	5
processed_transcrip	ENST00000484836	821	0	0	0	5
protein_coding	ENST00000583767	894	0	0	0	5
protein_coding	ENST00000327064	3032	1824	190	1015	16
protein_coding	ENST00000415183	2660	2196	26	435	14
protein_coding	ENST00000595948	2407	1767	621	16	10
protein_coding	ENST00000595948	2407	1767	621	16	10
protein_coding	ENST00000480003	1258	1110	90	55	11
protein_coding	ENST00000040877	5130	4863	0	264	30
protein_coding	ENST00000531612	938	866	0	72	9
protein_coding	ENST00000371320	2077	1164	411	499	18
protein_coding	ENST00000370521	6121	2952	359	2807	22
protein_coding	ENST00000407261	2147	1119	872	153	18
protein_coding	ENST00000358918	3578	2256	200	1119	17
protein_coding	ENST00000340344	4676	324	66	4283	3
protein_coding	ENST00000397873	2374	2049	223	99	21
protein_coding	ENST00000540509	2745	2742	0	0	24
protein_coding	ENST00000455059	1697	1194	455	45	9
protein_coding	ENST00000356531	1726	936	162	625	10
protein_coding	ENST00000356531	1726	936	162	625	10
protein_coding	ENST00000356531	1726	936	162	625	10
protein_coding	ENST00000283628	2074	1620	254	197	17
protein_coding	ENST00000457607	3748	3666	12	67	19
protein_coding	ENST00000329433	2114	1416	35	660	12
protein_coding	ENST00000512466	3763	0	0	0	2
protein_coding	ENST00000512466	3763	0	0	0	2
protein_coding	ENST00000340941	3665	1689	129	1844	17
protein_coding	ENST00000376406	7576	6267	648	658	15
protein_coding	ENST00000404765	3881	3486	290	102	26
protein_coding	ENST00000393118	4281	1674	221	2383	13
protein_coding	ENST00000448549	582	189	199	191	4
protein_coding	ENST00000448549	582	189	199	191	4
protein_coding	ENST00000356142	907	633	197	74	7
protein_coding	ENST00000421699	4260	3006	13	1238	26
protein_coding	ENST00000372739	7872	7431	110	328	57
protein_coding	ENST00000277010	1656	669	74	910	4
protein_coding	ENST00000371335	4030	1230	137	2660	9
protein_coding	ENST00000369878	15857	2625	188	13041	8
protein_coding	ENST00000423468	2009	1596	323	87	17
protein_coding	ENST00000369131	1133	534	599	0	13
protein_coding	ENST00000368836	1462	874	37	549	7
protein_coding	ENST00000368654	12678	9768	376	2531	15
protein_coding	ENST00000355394	3728	1527	224	1974	12
protein_coding	ENST00000423465	873	873	0	0	9
protein_coding	ENST00000344936	4128	2538	235	1352	20
protein_coding	ENST00000374789	6005	4068	326	1608	25
protein_coding	ENST00000333809	2658	1044	54	1557	8
protein_coding	ENST00000491200	2308	0	0	0	8
protein_coding	ENST00000394829	3149	1575	135	1436	14
protein_coding	ENST00000423381	3908	1764	124	2017	23
protein_coding	ENST00000298786	3614	2712	114	785	10
protein_coding	ENST00000371501	6842	6183	123	533	54
protein_coding	ENST00000371501	6842	6183	123	533	54
protein_coding	ENST00000265993	2734	1875	190	666	14
protein_coding	ENST00000282441	5386	1512	388	3483	9

protein_coding	ENST00000282441	5386	1512	388	3483	9
protein_coding	ENST00000339995	3978	2721	492	762	22
protein_coding	ENST00000278937	2620	645	129	1843	6
protein_coding	ENST00000530691	6307	0	0	0	16
protein_coding	ENST00000534624	2463	1938	277	245	9
protein_coding	ENST00000303296	7408	3645	305	3455	17
protein_coding	ENST00000263401	1745	594	17	1131	6
protein_coding	ENST00000525378	779	0	0	0	3
protein_coding	ENST00000526295	488	0	0	0	3
protein_coding	ENST00000528907	861	0	0	0	4
protein_coding	ENST00000334944	3094	1914	394	783	14
protein_coding	ENST00000309657	1485	987	44	451	8
protein_coding	ENST00000393695	7343	6345	332	663	27
protein_coding	ENST00000538328	1425	984	173	265	2
protein_coding	ENST00000529689	4014	762	435	2814	9
protein_coding	ENST00000393346	2340	1956	149	232	20
protein_coding	ENST00000393346	2340	1956	149	232	20
protein_coding	ENST00000534147	1022	630	7	382	5
protein_coding	ENST00000393223	4787	1713	663	2408	17
protein_coding	ENST00000427956	3701	1836	0	1862	14
protein_coding	ENST00000541351	717	511	127	79	6
protein_coding	ENST00000429722	1159	783	364	9	4
protein_coding	ENST00000546933	2154	996	1000	155	6
protein_coding	ENST00000542287	3443	3219	163	58	26
protein_coding	ENST00000551404	1630	1419	108	100	14
protein_coding	ENST00000550785	5345	2664	169	2509	29
protein_coding	ENST00000257570	2085	1542	271	269	6
protein_coding	ENST00000324774	5598	1764	829	3002	17
protein_coding	ENST00000355446	5796	3258	132	2403	19
protein_coding	ENST00000356219	8708	7563	156	986	48
protein_coding	ENST00000342628	3475	2403	114	955	10
protein_coding	ENST00000545668	3751	2910	80	758	27
protein_coding	ENST00000381000	4397	2214	41	2139	20
protein_coding	ENST00000399788	10763	5070	363	5327	28
protein_coding	ENST00000432191	3377	1302	72	2000	12
protein_coding	ENST00000420613	2155	1476	48	628	10
protein_coding	ENST00000394963	3658	1545	398	1712	13
protein_coding	ENST00000418425	2123	1119	19	982	9
protein_coding	ENST00000266987	1867	1098	483	283	9
protein_coding	ENST00000550713	548	415	133	0	5
protein_coding	ENST00000536427	2077	1878	88	108	13
protein_coding	ENST00000448157	1017	417	285	312	7
protein_coding	ENST00000300134	4034	2541	326	1164	22
protein_coding	ENST00000549307	3870	0	0	0	10
protein_coding	ENST00000441074	2323	0	0	0	2
protein_coding	ENST00000357008	6496	5736	164	593	40
antisense	ENST00000586338	723	0	0	0	2
protein_coding	ENST00000396801	3008	1731	208	1066	11
protein_coding	ENST00000543155	2127	825	482	817	8
protein_coding	ENST00000564245	1086	0	0	0	8
protein_coding	ENST00000266564	3170	1893	17	1257	15
protein_coding	ENST00000356859	3942	0	0	0	12
protein_coding	ENST00000530271	8643	8640	0	0	30
protein_coding	ENST00000434316	3050	2031	359	657	13
protein_coding	ENST00000375370	2648	1230	212	1203	12
protein_coding	ENST00000375299	2378	1428	56	891	10
protein_coding	ENST00000413537	1064	690	36	335	11
protein_coding	ENST00000361847	1940	1257	232	448	12
protein_coding	ENST00000392634	4672	3747	112	810	23
protein_coding	ENST00000331320	2876	2145	214	514	21
antisense	ENST00000557335	1398	0	0	0	4
protein_coding	ENST00000324366	2531	1911	224	393	17
protein_coding	ENST00000553710	255	49	0	206	3
protein_coding	ENST00000246163	897	288	170	436	4
protein_coding	ENST00000251038	4194	2208	225	1758	17
protein_coding	ENST00000393218	2115	1299	338	475	15

protein_coding	ENST00000216277	4519	2235	220	2061	22
protein_coding	ENST00000398181	3205	1866	314	1022	15
protein_coding	ENST00000566767	617	171	0	446	4
protein_coding	ENST00000307979	794	204	31	556	2
protein_coding	ENST00000340965	5480	990	668	3819	9
protein_coding	ENST00000419739	1718	1659	59	0	20
protein_coding	ENST00000308275	3483	1209	105	2166	7
protein_coding	ENST00000361188	4087	1818	1212	1054	14
protein_coding	ENST00000569715	3775	2283	77	1412	22
protein_coding	ENST00000301740	9353	8256	549	545	15
protein_coding	ENST00000357402	2865	2679	138	45	15
protein_coding	ENST00000570255	926	695	0	231	6
protein_coding	ENST00000565112	879	485	41	353	5
protein_coding	ENST00000562435	7481	1575	385	5518	9
protein_coding	ENST00000321919	3772	1659	77	2033	16
protein_coding	ENST00000262133	4906	3417	137	1349	22
lincRNA	ENST00000560208	735	0	0	0	2
protein_coding	ENST00000539578	2729	2304	313	109	20
protein_coding	ENST00000339507	4238	2076	830	1329	19
protein_coding	ENST00000522091	863	297	130	433	3
protein_coding	ENST00000302516	6969	3651	211	3104	26
protein_coding	ENST00000433195	4513	2535	800	1175	26
protein_coding	ENST00000569197	530	467	0	63	4
protein_coding	ENST00000567298	4572	1560	82	2927	17
protein_coding	ENST00000567298	4572	1560	82	2927	17
protein_coding	ENST00000565583	1068	591	0	477	5
protein_coding	ENST00000301015	8072	7563	247	259	51
protein_coding	ENST00000389301	5451	4365	31	1052	43
protein_coding	ENST00000268712	10720	7320	258	3139	46
protein_coding	ENST00000268712	10720	7320	258	3139	46
protein_coding	ENST00000313485	6050	5384	0	666	11
protein_coding	ENST00000327031	4338	3807	226	302	30
protein_coding	ENST00000327031	4338	3807	226	302	30
protein_coding	ENST00000261497	5216	1575	204	3434	13
protein_coding	ENST00000527372	7597	6162	181	1251	42
protein_coding	ENST00000574709	658	0	0	0	2
protein_coding	ENST00000358273	12425	8517	383	3522	58
protein_coding	ENST00000577908	4672	1479	106	3084	14
protein_coding	ENST00000577908	4672	1479	106	3084	14
protein_coding	ENST00000353139	9962	7149	482	2328	56
protein_coding	ENST00000427569	1322	1236	36	47	12
protein_coding	ENST00000427569	1322	1236	36	47	12
protein_coding	ENST00000362042	4774	2316	616	1839	6
protein_coding	ENST00000576461	382	234	92	53	3
protein_coding	ENST00000007722	3665	3198	0	464	25
antisense	ENST00000575890	1938	0	0	0	2
antisense	ENST00000575890	1938	0	0	0	2
antisense	ENST00000575890	1938	0	0	0	2
protein_coding	ENST00000225792	2554	1842	402	307	13
protein_coding	ENST00000321892	11292	9138	61	2090	30
protein_coding	ENST00000577985	2257	1080	988	186	6
protein_coding	ENST00000405159	2865	621	65	2176	4
protein_coding	ENST00000350303	2191	1899	122	167	19
protein_coding	ENST00000335146	3519	2205	54	1257	20
protein_coding	ENST00000571877	1195	0	0	0	2
protein_coding	ENST00000428789	4053	2403	103	1544	16
protein_coding	ENST00000423034	3984	1737	308	1936	11
protein_coding	ENST00000380358	7356	6177	1	1175	40
protein_coding	ENST00000576544	1720	1125	64	528	7
protein_coding	ENST00000402252	1346	1038	26	279	8
protein_coding	ENST00000360416	7762	6021	139	1599	43
protein_coding	ENST00000539164	886	426	23	434	7
antisense	ENST00000592893	918	0	0	0	3
protein_coding	ENST00000300952	3790	1404	515	1868	8
protein_coding	ENST00000592062	2579	1977	571	28	18
protein_coding	ENST00000242786	3188	2505	80	600	20

protein_coding	ENST00000588136	2577	1953	497	124	19
protein_coding	ENST00000598068	1341	739	0	602	5
protein_coding	ENST00000270061	1768	1155	294	316	18
protein_coding	ENST00000303088	1407	780	474	150	10
protein_coding	ENST00000585603	3385	660	2302	420	7
protein_coding	ENST00000588582	3601	0	0	0	7
protein_coding	ENST00000355415	2124	1440	170	511	10
protein_coding	ENST00000262633	1680	1440	105	132	10
protein_coding	ENST00000308370	5142	4869	0	270	34
protein_coding	ENST00000308370	5142	4869	0	270	34
protein_coding	ENST00000597608	1727	0	0	0	1
protein_coding	ENST00000597608	1727	0	0	0	1
antisense	ENST00000590796	829	0	0	0	4
protein_coding	ENST00000595072	721	0	0	0	4
protein_coding	ENST00000246785	1431	1002	258	168	7
protein_coding	ENST00000593992	603	0	0	0	4
protein_coding	ENST00000372412	7350	5847	234	1266	38
protein_coding	ENST00000085068	1122	663	137	319	6
protein_coding	ENST00000252674	1931	1677	164	87	12
protein_coding	ENST00000394601	3193	1650	46	1494	15
protein_coding	ENST00000369483	7253	3642	705	2903	12
protein_coding	ENST00000196061	2940	2181	27	729	19
protein_coding	ENST00000442702	4707	2010	1067	1627	21
protein_coding	ENST00000314136	3453	804	370	2276	7
protein_coding	ENST00000369064	2729	2154	34	538	18
protein_coding	ENST00000368830	1296	801	85	407	7
protein_coding	ENST00000368521	1708	759	199	747	7
protein_coding	ENST00000428931	3988	3261	167	557	27
protein_coding	ENST00000428931	3988	3261	167	557	27
protein_coding	ENST00000368404	1300	663	62	572	6
protein_coding	ENST00000355379	1537	963	279	292	8
protein_coding	ENST00000445566	899	538	0	361	5
protein_coding	ENST00000608310	2635	2253	251	128	18
protein_coding	ENST00000271469	2344	1566	302	473	11
protein_coding	ENST00000465089	11321	0	0	0	3
protein_coding	ENST00000359523	5010	807	202	3998	6
protein_coding	ENST00000367669	3033	2193	515	322	20
protein_coding	ENST00000367580	7704	1023	5	6673	6
protein_coding	ENST00000462677	1628	410	0	1218	6
protein_coding	ENST00000340006	1952	579	191	1179	6
protein_coding	ENST00000367005	4246	1485	141	2617	11
protein_coding	ENST00000338179	3774	1845	126	1800	14
protein_coding	ENST00000284563	2856	2451	26	376	15
protein_coding	ENST00000366731	1361	492	794	72	6
protein_coding	ENST00000488353	2469	588	1786	92	6
protein_coding	ENST00000400181	3059	2628	104	324	21
protein_coding	ENST00000478849	880	0	0	0	5
protein_coding	ENST00000366548	3473	2538	593	339	16
protein_coding	ENST00000495785	1390	207	16	1164	5
protein_coding	ENST00000447431	3175	2748	43	381	18
protein_coding	ENST00000373151	3324	2523	216	582	17
protein_coding	ENST00000485039	814	0	0	0	4
protein_coding	ENST00000485039	814	0	0	0	4
protein_coding	ENST00000373019	3673	1503	956	1211	17
protein_coding	ENST00000564288	24828	22665	777	1383	101
protein_coding	ENST00000372621	3199	1773	508	915	19
protein_coding	ENST00000372572	5352	1866	312	3171	15
protein_coding	ENST00000371933	5767	1485	977	3302	11
protein_coding	ENST00000531819	4520	0	0	0	8
protein_coding	ENST00000475501	721	721	0	0	6
protein_coding	ENST00000475501	721	721	0	0	6
protein_coding	ENST00000371429	5134	2022	599	2510	18
protein_coding	ENST00000371370	2037	1284	521	229	9
protein_coding	ENST00000403491	9487	1527	484	7473	11
protein_coding	ENST00000251157	7110	6387	34	686	49
protein_coding	ENST00000489188	784	0	0	0	3

protein_coding	ENST00000417775	2301	987	505	806	10
protein_coding	ENST00000417775	2301	987	505	806	10
protein_coding	ENST00000370630	6600	1155	49	5393	7
protein_coding	ENST00000344356	504	501	0	0	3
protein_coding	ENST00000337907	8026	4698	635	2690	24
protein_coding	ENST00000401030	727	423	24	277	6
protein_coding	ENST00000377298	5221	2943	793	1482	19
protein_coding	ENST00000405977	5137	1566	625	2943	5
protein_coding	ENST00000381653	1446	822	0	621	9
antisense	ENST00000412178	489	0	0	0	3
protein_coding	ENST00000246194	1789	918	502	366	10
protein_coding	ENST00000317619	2204	1611	395	195	18
protein_coding	ENST00000407261	2147	1119	872	153	18
protein_coding	ENST00000338074	6266	2643	161	3459	22
protein_coding	ENST00000338074	6266	2643	161	3459	22
processed_transcrip	ENST00000414142	2102	0	0	0	8
protein_coding	ENST00000372889	1489	228	585	673	6
protein_coding	ENST00000372889	1489	228	585	673	6
protein_coding	ENST00000435836	670	525	145	0	7
protein_coding	ENST00000379019	5454	2016	213	3222	20
protein_coding	ENST00000370507	2665	2166	96	400	11
protein_coding	ENST00000361093	2149	1299	91	756	11
protein_coding	ENST00000486544	586	0	0	0	2
protein_coding	ENST00000404664	1606	885	110	608	6
protein_coding	ENST00000490315	2301	0	0	0	4
protein_coding	ENST00000490315	2301	0	0	0	4
protein_coding	ENST00000357586	4176	2847	187	1139	23
protein_coding	ENST00000485559	2653	0	0	0	3
protein_coding	ENST00000380817	8008	5679	184	2142	41
protein_coding	ENST00000289371	5777	3660	202	1912	24
protein_coding	ENST00000495559	3834	0	0	0	4
protein_coding	ENST00000350878	3919	3837	0	79	29
protein_coding	ENST00000350878	3919	3837	0	79	29
protein_coding	ENST00000409400	2143	1317	335	488	14
protein_coding	ENST00000252804	6808	4437	51	2317	23
antisense	ENST00000412276	829	0	0	0	7
protein_coding	ENST00000392318	5082	3408	247	1424	31
protein_coding	ENST00000409151	1979	1698	100	178	12
protein_coding	ENST00000434813	2026	1578	335	110	13
protein_coding	ENST00000392059	2005	1026	266	710	11
protein_coding	ENST00000389044	6405	6120	169	113	42
protein_coding	ENST00000389044	6405	6120	169	113	42
protein_coding	ENST00000392018	3313	1872	114	1324	19
protein_coding	ENST00000409538	6138	3207	496	2432	17
protein_coding	ENST00000359541	3992	2733	430	826	19
protein_coding	ENST00000476552	587	0	0	0	4
protein_coding	ENST00000379632	1752	1128	140	481	14
protein_coding	ENST00000280527	5912	3108	367	2434	17
protein_coding	ENST00000406220	430	225	149	53	2
protein_coding	ENST00000410073	479	438	14	24	5
protein_coding	ENST00000454893	527	288	62	174	5
protein_coding	ENST00000264094	3502	2259	72	1168	14
protein_coding	ENST00000360635	4859	600	897	3359	8
protein_coding	ENST00000489060	4615	0	0	0	3
protein_coding	ENST00000406780	3674	2733	537	401	16
protein_coding	ENST00000361309	1285	969	106	207	11
protein_coding	ENST00000431670	6127	3759	411	1954	18
protein_coding	ENST00000240922	6135	507	325	5300	5
protein_coding	ENST00000311127	9156	4143	68	4942	17
protein_coding	ENST00000489960	1903	1119	406	375	12
protein_coding	ENST00000441626	1040	537	149	351	5
protein_coding	ENST00000508660	943	943	0	0	9
protein_coding	ENST00000425847	1457	1125	162	167	11
protein_coding	ENST00000420115	3621	330	202	3086	5
protein_coding	ENST00000420115	3621	330	202	3086	5
protein_coding	ENST00000473073	1964	0	0	0	11

protein_coding	ENST00000469350	723	688	35	0	7
protein_coding	ENST00000460393	5622	2250	120	3249	23
protein_coding	ENST00000317096	1414	1137	61	213	10
protein_coding	ENST00000465218	1310	0	0	0	6
protein_coding	ENST00000346964	5034	2778	190	2063	26
protein_coding	ENST00000454389	4159	3777	221	158	26
protein_coding	ENST00000283628	2074	1620	254	197	17
protein_coding	ENST00000421307	3814	2163	423	1225	29
protein_coding	ENST00000450296	1570	1149	90	328	10
protein_coding	ENST00000450296	1570	1149	90	328	10
protein_coding	ENST00000342790	1336	999	258	76	8
protein_coding	ENST00000426837	8920	6891	88	1938	21
protein_coding	ENST00000434592	2342	597	319	1423	21
protein_coding	ENST00000002829	3802	2355	484	960	19
processed_transcrip	ENST00000416295	1088	0	0	0	5
protein_coding	ENST00000320295	2324	1305	427	589	6
protein_coding	ENST00000418577	1533	666	576	288	3
protein_coding	ENST00000474012	1760	1107	217	433	11
protein_coding	ENST00000460680	3937	2187	472	1275	17
protein_coding	ENST00000419752	8762	7266	0	1493	45
protein_coding	ENST00000464233	6742	4953	114	1672	31
protein_coding	ENST00000421188	1323	1323	0	0	7
protein_coding	ENST00000512454	1543	1269	216	55	11
protein_coding	ENST00000304527	5273	2370	1090	1810	21
protein_coding	ENST00000381897	4886	3444	134	1305	25
protein_coding	ENST00000508466	3051	2753	0	298	21
protein_coding	ENST00000264313	6299	1743	418	4135	8
protein_coding	ENST00000502972	1916	0	0	0	2
protein_coding	ENST00000507166	2550	2547	0	0	24
protein_coding	ENST00000507166	2550	2547	0	0	24
protein_coding	ENST00000507166	2550	2547	0	0	24
protein_coding	ENST00000502762	2096	1260	451	382	9
protein_coding	ENST00000295491	1643	426	113	1101	6
protein_coding	ENST00000508553	789	0	0	0	3
protein_coding	ENST00000455264	2898	2715	80	100	24
protein_coding	ENST00000254900	4388	3705	372	308	27
protein_coding	ENST00000297183	8319	7851	124	341	36
protein_coding	ENST00000297183	8319	7851	124	341	36
protein_coding	ENST00000506630	897	0	0	0	3
protein_coding	ENST00000354546	2593	2019	228	343	26
protein_coding	ENST00000354546	2593	2019	228	343	26
protein_coding	ENST00000354546	2593	2019	228	343	26
protein_coding	ENST00000311086	1562	555	224	780	4
protein_coding	ENST00000356834	5173	3750	38	1382	22
protein_coding	ENST00000511367	5305	4122	245	935	35
protein_coding	ENST00000265113	4170	1626	476	2065	10
protein_coding	ENST00000354209	1918	1722	185	8	10
protein_coding	ENST00000325110	3506	1560	207	1736	11
protein_coding	ENST00000230640	4192	3126	254	809	27
protein_coding	ENST00000424459	4683	1479	1274	1927	13
protein_coding	ENST00000424459	4683	1479	1274	1927	13
protein_coding	ENST00000515835	590	87	334	166	5
protein_coding	ENST00000545191	2730	1257	16	1454	13
protein_coding	ENST00000264668	3274	2175	30	1066	15
antisense	ENST00000511775	581	0	0	0	2
protein_coding	ENST00000398153	918	612	43	260	6
protein_coding	ENST00000398153	918	612	43	260	6
protein_coding	ENST00000398153	918	612	43	260	6
protein_coding	ENST00000337057	4457	3015	182	1257	20
protein_coding	ENST00000400822	7593	5502	0	2088	34
protein_coding	ENST00000443868	1574	1338	88	145	12
protein_coding	ENST00000338130	1515	693	712	107	10
protein_coding	ENST00000376575	3949	2757	333	856	20
protein_coding	ENST00000375537	3965	3630	7	325	28
protein_coding	ENST00000395289	4898	2508	1601	786	14
protein_coding	ENST00000469104	704	264	213	224	3

protein_coding	ENST00000372836	1704	834	371	496	6
protein_coding	ENST00000493786	551	0	0	0	4
protein_coding	ENST00000493786	551	0	0	0	4
protein_coding	ENST00000309268	2303	1386	619	295	8
protein_coding	ENST00000370014	6645	3336	619	2687	24
protein_coding	ENST00000481508	989	444	435	107	7
antisense	ENST00000427392	592	0	0	0	4
protein_coding	ENST00000466855	1894	0	0	0	4
protein_coding	ENST00000275418	3251	2655	0	596	23
protein_coding	ENST00000490912	5328	0	0	0	10
protein_coding	ENST00000409436	954	498	107	346	4
protein_coding	ENST00000494488	6976	1143	0	5830	12
protein_coding	ENST00000265748	4787	3372	221	1191	24
protein_coding	ENST00000494076	2190	1893	70	224	11
protein_coding	ENST00000495446	2782	0	0	0	3
protein_coding	ENST00000345660	2027	1800	28	196	11
protein_coding	ENST00000456758	4514	2922	0	1589	25
processed_transcrip	ENST00000310771	3634	0	0	0	18
protein_coding	ENST00000276699	1858	1101	176	578	5
processed_transcrip	ENST00000513868	1699	0	0	0	8
protein_coding	ENST00000519540	2021	972	401	645	13
protein_coding	ENST00000414097	3755	1182	868	1702	16
protein_coding	ENST00000356994	5218	4965	7	243	37
protein_coding	ENST00000325083	6820	6072	439	306	39
protein_coding	ENST00000397677	3403	1359	648	1393	11
protein_coding	ENST00000521492	786	162	470	151	3
protein_coding	ENST00000406452	3063	942	160	1958	10
protein_coding	ENST00000552516	5933	1521	56	4353	9
protein_coding	ENST00000374586	7980	2637	32	5308	18
protein_coding	ENST00000374541	6703	2739	105	3856	26
protein_coding	ENST00000265960	3395	1566	333	1493	12
protein_coding	ENST00000372406	7353	1800	507	5043	20
protein_coding	ENST00000357304	11042	6687	55	4297	31
protein_coding	ENST00000371601	1494	975	214	302	9
protein_coding	ENST00000489219	3293	2523	28	739	16
protein_coding	ENST00000485617	3460	0	0	0	10
protein_coding	ENST00000376598	2404	2115	0	286	17
protein_coding	ENST00000376263	2942	1392	224	1323	17
protein_coding	ENST00000375234	2863	678	0	2182	6
protein_coding	ENST00000434230	899	533	366	0	5
protein_coding	ENST00000372479	1662	1239	31	389	7
protein_coding	ENST00000371145	4393	3804	281	305	35
protein_coding	ENST00000371113	5162	2703	165	2291	24
protein_coding	ENST00000370777	784	642	101	38	5
protein_coding	ENST00000464506	2039	603	279	1154	3
protein_coding	ENST00000490265	4259	0	0	0	23
protein_coding	ENST00000370475	4308	1896	128	2281	17
protein_coding	ENST00000543994	5033	3777	108	1145	28
protein_coding	ENST00000379295	2768	1137	1418	210	17
protein_coding	ENST00000379295	2768	1137	1418	210	17
protein_coding	ENST00000357033	13956	11055	207	2691	79
protein_coding	ENST00000342274	6390	5163	363	861	15
protein_coding	ENST00000376358	1176	876	154	143	8
protein_coding	ENST00000217964	5866	1731	640	3492	18

Max_intron
39348
37206
37206
26919
525
28816
8791
4089
822
822
2995
33030
3360
23617
23617
15470
9701
867
120508
56361
5138
58843
11818
5096
16043
3808
1244
1244
1244
14336
11744
2038
3016
3016
22173
3939
2267
37082
9670
9670
12443
5553
14043
2368
12656
129607
32891
7403
14483
8166
46241
16899
47532
179441
27363
1620
16708
12254
54362
25436
25436
17209
48063

48063
2060
3911
17201
1216
41000
8718
1847
2783
684
3250
506
34010
3566
7837
37461
37461
2358
26157
9081
6064
2929
6100
43791
22482
33405
5782
24253
81822
40457
3730
5277
21951
18220
42322
7424
3848
2908
844
2253
4441
15333
3271
19388
321
4844
3321
8772
3109
11335
7312
7425
59319
13313
25148
7049
13764
21723
11619
18610
1484
1730
3359
10072
18595
14069

17456
45920
395
3883
22548
6695
12491
10937
22534
3489
10006
4437
14880
23486
12380
8296
4291
13444
8286
11143
6005
17450
438
18258
18258
1632
35828
6683
21088
21088
4736
2008
2008
14498
44856
295
60615
6592
6592
70108
4214
4214
4759
103792
7433
7738
7738
7738
1845
27604
3227
14587
481
3605
156
20258
79442
4582
955
1555
18452
9339
24897
2267
2660
6906

14096
642
7645
2831
2333
6074
6590
2786
4859
4859
0
0
66948
259
3211
12200
54754
5297
31741
4119
50877
13122
5861
4850
5043
1257
5673
5867
5867
611
1894
15593
34232
10430
24959
43342
39909
14979
15754
11068
11069
4907
85468
1182
2196
13794
24357
6472
3742
5597
14509
7909
7909
8015
33717
4073
37307
11501
8442
4681
4681
14930
5678
188841
32362
319

5710
5710
13813
1280
136616
16293
51255
26825
6965
5217
40463
22550
5138
18869
18869
5044
50608
50608
37871
13667
3760
4326
1339
7019
1460
1460
21377
771
6649
22648
2476
26546
26546
30653
51444
307576
33710
37934
2860
7705
42018
42018
7102
76015
13185
4904
107823
44472
20464
703
5865
12875
4555
2267
64366
11024
27908
22316
26764
10571
12277
12273
16570
8888
8888
21773

39516
29901
18571
535
88421
31931
14336
25712
4720
4720
17040
90204
72854
14060
459
5990
1196
25909
1662
13061
464506
10558
2782
38664
15028
19493
36342
3361
848961
848961
848961
1864
1389
10822
33964
7118
33832
33832
1522
9999
9999
9999
4018
8685
12811
41443
20930
9307
14181
37675
37675
299768
10196
4834
356438
15808
15808
15808
107329
37315
4360
3467
3009
3691
1536
895

4755
3039
3039
1706
18957
4728
30575
1789
30442
4215
4281
30653
12177
3803
712
4160
15833
7813
13010
80870
35374
15721
8152
12645
5867
1576
18561
23554
18350
35010
72245
21564
7273
3277
11113
5379
7944
2248
6448
6231
27121
59512
8725
5556
14527
5384
9704
3058
21191
21191
248725
99486
3033
138425

Supplementary Table 3. Primers used for qPCR and AS PCR.

Gene Name	Products	Forward Prime	Reverse Primer
AARSD1	307(IN)/190(EX)	CCCCTCTATGACTGCAGAGC	CCGGTTCCCAGACAGAAATA
APLP2	159(IN)/123(EX)	TCTCAGAGACCCCTGTGGAC	CCTGCTCTCCCACTCCAGAT
BAG6	213(IN)/108(EX)	ACACAGCCTGGTGGTGTTC	GGTTGGAGCTGTTGGGAAG
BCAR1	375(in)/97(ex)	GATGGGGCATGACATCTACC	CGATGGAGGAACGTCGTAG
C20orf7	271(in)/173(ex)	AATGACCTGGGACATCTGCT	AGCATTGTGTCTCGATGCAG
DDX27	252(in)/78(ex)	ATTGGGGAAGGACATCTGTG	CGAAGAGCTGCTTCCTGAGA
EFCAB14	333(IN)/141(EX)	CCTGGAAGCAACCAGATCAT	TGTTCTGTTGGTGACTTGATCC
GLRX3	182(in)/107(ex)	CAGTGTGCACAGATGAACGA	GCATGTGCACCATCTAATCG
HERC4	241(in)/138(ex)	CAAGCAATGGTCATTGGAAA	ACTCCTCACCACCTCCTGTG
HNRNPDL	231(in)/126(ex)	ACTTATGGCAAGGCATCTCG	TCTTCAATGTCGTCCTGCAA
KPNA1	306(in)/180(ex)	AGTGGTTTCTCCTGCTTTGC	GCTGATCCTCCAGAAGTTGC
MRPL3	239(IN)/140(EX)	CCAAGGATGGTCAAAAGCAT	GCCTGGTTTAATTGCAGCAT
MUM1	260(IN)/147(EX)	AAGCCATTGCTTCCTCGTTA	TACCTGTCCCTGCAGAGCTT
MYL6	240(IN)/195(EX)	GCATGAGGACAGCAATGGTT	CTGACGGCAAACATCATCC
MYO18A	233(IN)/188(EX)	GCAGGCTGACCTAAAGTTGG	CCTTGGAAGGTCCCTTGTTT
NCOR1	136(IN)/109(EX)	CCAGCCATCAGATACCAAGG	CCTTTGTTTTCTTGCATGATTTC
POLDIP3	235(in)/184(short in)/97(	AGCTCACCAAAACCATCCAG	CCTGAGGCTGCAAACTTCAT
PSMD2	308(in)/94(ex)	CGTGAGTGCCTCAAGTATCG	GCTCAGGCACGTAATTCACA
PTPN3	255(IN)/90(EX)	GACTATGGGCTCTCGGAACA	TCCGTTTCCGTGATGTAGGT
ROBO1	175(IN)/148(EX)	CCTACGCGGGTATCAGAAAA	TCATTGTGGTTGTTGCCAGT
SACM1L	296(in)/168(ex)	TCCTCCTTCAGCTGTCACAA	ATAGCCGCTGCAAAGTATGG
SMARCD1	301(IN)/178(EX)	GGCTTTTGAAAGGAAACTGG	TGGCCCATACAGGTCTTTGT
SPATA5L1	239(in)/209(ex)	CTCAGCCAGCAAGACAGGAT	TTCCAGGTCGTAACAAAGCA
SPTAN1	337(IN)/277(EX)	GTGAACGATCGTCAGGGTTT	CTCCGACCTCCTCACTTGTC
SRSF2	243(in)/139(ex)	GTGTCCAAGAGGGAATCCAA	GTGGTCCTTTTTCCCCAAGT
TARBP1	322(IN)/226(EX)	ACGCATTCAGGAGCATTTCT	TTGCTGAGACCAGTCACCTG
TPCN1	128(in)/83(ex)	TTTGATGAGCTTCCCAGGAC	GTCTCCACGAGGATCCAGAC
HNRNPM	165(IN)/48(EX)	GAACATGCCAGGAGAGCAAT	TGTGCTTCCAAGTCTTCCAG
KIF18B	445(IN)/218(EX)	CCAGCAGCACTTTGAAGAGG	CAAGAGGGGTATCCAGCAGA
SNHG1	240(IN)/56(EX)	CCATTGAAAAGCCTTCAGAGC	GAGCCAAGCAGGTTATTGGT
SOD2	327(IN)/180(EX)	ACTGCTGGGGATTGATGTGT	ttttcaatcacttgcccaat; TCTTGCTGGGATCATTAGGG
TP53BP1	162(IN)/202(EX)	TGGAAATTCCTCCTTTCAACA	aggcctcttctcaacacagc; TCATGGACCCAGACATGAGA
TSSC4	254(IN)/62(EX)	ATGGCTGAGGCAGGAACA	ATGCCTCTCAGATGGAATGG
